# Identification and Validation of a Metabolism-Related Prognostic Signature Associated with M2 Macrophage Infiltration in Gastric Cancer

**DOI:** 10.3390/ijms241310625

**Published:** 2023-06-25

**Authors:** Yunze Liu, Haocheng Zheng, Anna Meilin Gu, Yuan Li, Tieshan Wang, Chengze Li, Yixiao Gu, Jie Lin, Xia Ding

**Affiliations:** 1 The First Clinical Medical College, Beijing University of Chinese Medicine, Beijing 100029, China; 2 School of Traditional Chinese Medicine, Beijing University of Chinese Medicine, Beijing 100029, China; 3 Biology Department: Physiology, University of Washington, Seattle, WA 98105, USA; 4 National Institute of TCM Constitution and Preventive Medicine, Beijing University of Chinese Medicine, Beijing 100029, China; 5 Beijing Research Institute of Chinese Medicine, Beijing University of Chinese Medicine, Beijing 100029, China

**Keywords:** M2 macrophage, gastric cancer, prognosis, metabolism, tumor microenvironment

## Abstract

High levels of M2 macrophage infiltration invariably contribute to poor cancer prognosis and can be manipulated by metabolic reprogramming in the tumor microenvironment. However, the metabolism-related genes (MRGs) affecting M2 macrophage infiltration and their clinical implications are not fully understood. In this study, we identified 173 MRGs associated with M2 macrophage infiltration in cases of gastric cancer (GC) using the TCGA and GEO databases. Twelve MRGs were eventually adopted as the prognostic signature to develop a risk model. In the high-risk group, the patients showed poorer survival outcomes than patients in the low-risk group. Additionally, the patients in the high-risk group were less sensitive to certain drugs, such as 5-Fluorouracil, Oxaliplatin, and Cisplatin. Risk scores were positively correlated with the infiltration of multiple immune cells, including CD8+ T cells and M2 macrophages. Furthermore, a difference was observed in the expression and distribution between the 12 signature genes in the tumor microenvironment through single-cell sequencing analysis. In vitro experiments proved that the M2 polarization of macrophages was suppressed by Sorcin-knockdown GC cells, thereby hindering the proliferation and migration of GC cells. These findings provide a valuable prognostic signature for evaluating clinical outcomes and corresponding treatment options and identifying potential targets for GC treatment.

## 1. Introduction

Gastric cancer (GC) is the fifth most common malignancy and the fourth leading cause of cancer-associated death worldwide [1]. The therapeutic strategies for GC include surgery, chemotherapy, targeted therapy, and radiotherapy [2]. However, the efficacy of these current treatments has not yet met expectations. Effective prognostic and treatment evaluation is necessary in order to guide treatment decisions in clinical settings. Classically, pathological tumor node metastasis (TNM) staging is used as a common prediction method applied to cancer. However, even patients with the same TNM pathological stage usually exhibit different survival outcomes [3]. Tumor heterogeneity makes it difficult to accurately predict disease development via pathological staging, and differences in genetic characteristics can explain such heterogeneity. Exploring biological gene signatures is an exciting strategy for identifying robust prognostic biomarkers and may contribute to clinical decision making with respect to cancer.

In the tumor microenvironment (TME) of solid tumors, infiltrating macrophages are particularly abundant [4]. High plasticity and heterogeneity are the hallmarks of macrophages. In response to different signals from the TME, tumor-associated macrophages (TAMs) may be phenotypically polarized to the M1 (pro-inflammatory) or M2 (anti-inflammatory) states [5]. M2 macrophages secrete various cancer-inducing molecules (such as CHI3L1 and TGF-β), which stimulate the proliferation and metastasis of tumor cells [6,7]. Additionally, abundant M2 macrophages can remodel an immunosuppressive TME by inhibiting the immune response of T cells, thereby promoting the immune escape of tumor cells and resistance to immunotherapy [8,9]. Clinical and experimental evidence support the notion that the abundance of infiltrating M2 macrophages is unfavorable with regard to the prognosis of patients with various cancers [10]. Recently, the mechanisms underlying M2 macrophage infiltration have garnered increasing attention and have become the focus of anti-cancer research. 

Previous studies suggest that metabolic alterations in the TME create pro-tumorigenic and immunosuppressive microenvironments by manipulating the differentiation and activity of immune cells [11,12,13,14]. The tumor-specific metabolic reprogramming driven by the alteration of metabolism-related gene (MRG) expression results in metabolic changes in the TME and considerably regulates M2 macrophage infiltration in the TME. For example, SREBP overexpression in tumor cells leads to the deregulation of lipid metabolism, which promotes the formation of a lipid-enriched TME [15]. Changes in the TME polarize macrophages to the M2 phenotype by stimulating unconventional endoplasmic reticulum stress responses in TAMs [16]. In addition, PINK1 deficiency causes the Warburg effect in GC cells. This metabolic alteration subsequently induces the M2 polarization of TAMs [17]. Thus, the consideration of changes in MRG expression can help researchers explore valuable biomarkers for evaluating M2 macrophage infiltration and patient prognosis. 

In this study, we identified MRGs that affected the abundance of infiltrating M2 macrophages and constructed a 12-MRG prognostic signature for a risk model of GC. We confirmed the effectiveness of this prognostic signature in validation cohorts and used it to assess the efficacy of anti-cancer drugs. We further investigated the mechanism of action of the prognostic signature, especially regarding its relationship with the immune microenvironment in GC. We validated the expression of Sorcin (SRI) in GC cells and its role in the M2 polarization of macrophages in vitro. These findings may help predict GC prognosis and provide guidance for clinical decision making and potential treatment targets. 

## 2. Results

### 2.1. Metabolic Programming Involved in M2 Macrophage Infiltration Influencing GC Prognosis

**Figure 1:**
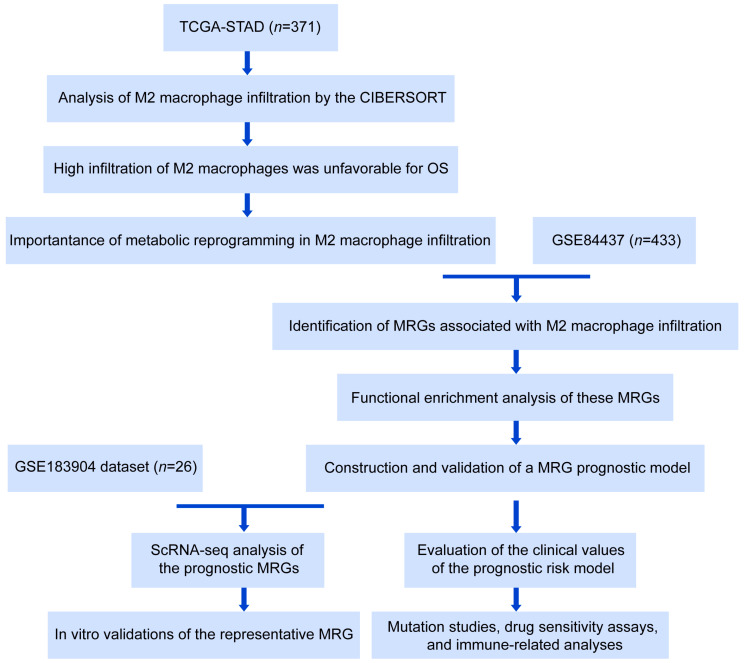
The study flowchart. STAD: stomach adenocarcinoma; TCGA: The Cancer Genome Atlas; OS: overall survival; MRGs: metabolism-related genes; scRNA-seq: single-cell sequencing.

**Figure 2:**
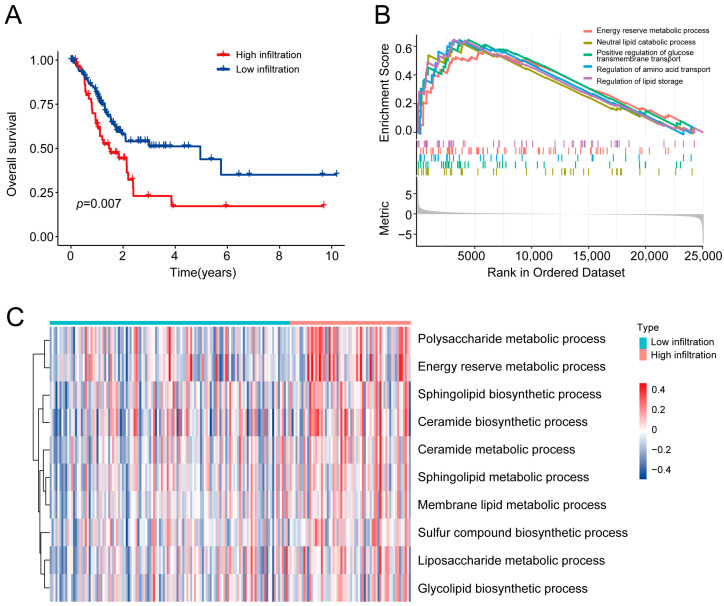
Identification of metabolic programming significantly associated with M2 macrophage infiltration. (**A**) Kaplan–Meier curves showing the difference in overall survival between the high- and low-M2-macrophage-infiltration groups. (**B**) GSEA showing the enrichment of metabolism-related pathways in patients with a high infiltration level of M2 macrophages. (**C**) GSVA between the high- and low-M2-macrophage-infiltration groups. GSEA: Gene Set Enrichment Analysis; GSVA: Gene Set Variation Analysis.

A flow chart describing this study is shown in Figure 1. It has been reported that high levels of M2 macrophage infiltration accelerate cancer development [18,19]. We evaluated the effect of the M2 macrophage infiltration degree on GC prognosis using the CIBERSORT algorithm based on the TCGA-STAD dataset. Kaplan–Meier survival analysis showed that patients with GC samples presenting high infiltration of M2 macrophages had significantly shorter overall survival (OS) compared to patients with low M2 macrophage infiltration (Figure 2A). Gene Set Enrichment Analysis (GSEA) was conducted to explore potential biological mechanisms associated with M2 macrophage infiltration. Multiple metabolic pathways were significantly enriched in the high-M2-macrophage-infiltration group, such as neutral lipid catabolic processes, energy reserve metabolic processes, and the regulation of amino acid transport (Figure 2B). Furthermore, Gene Set Variation Analysis (GSVA) was conducted; the heatmap of the GSVA scores also indicated that various metabolic pathways were activated in the high-infiltration group (Figure 2C). These findings suggest that metabolic reprogramming may contribute significantly to the infiltration of M2 macrophages in GC. This provides an essential basis for us to evaluate the role of M2 macrophage-associated MRGs in GC prognosis.

### 2.2. Identification of MRGs Associated with M2 Macrophage Infiltration

**Figure 3:**
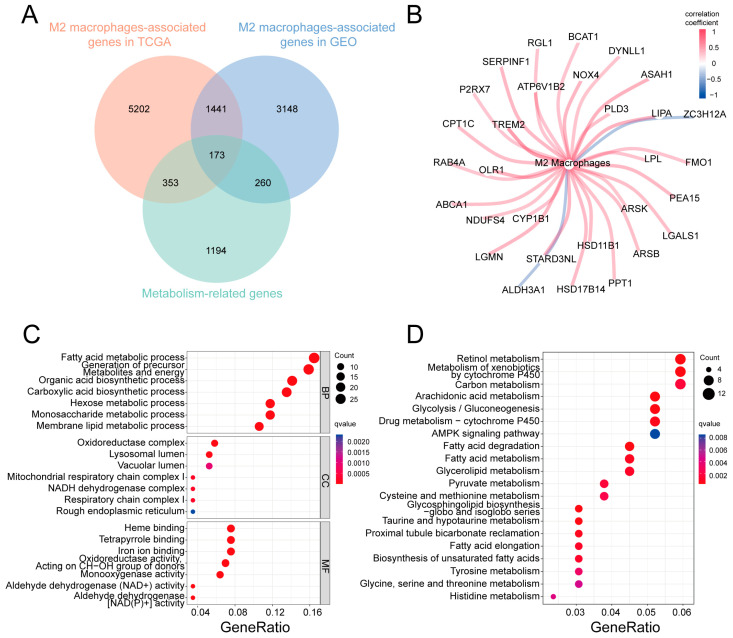
Identification of MRGs associated with M2 macrophage infiltration. (**A**) Venn diagram of MRGs associated with M2 macrophage infiltration. (**B**) Co-expression network diagram of the top 30 MRGs associated with M2 macrophage infiltration. (**C**) Bubble plots displaying the GO analysis results of all 173 MRGs associated with M2 macrophage infiltration. (**D**) Bubble plots displaying KEGG analysis results of the 173 MRGs associated with M2 macrophage infiltration. MRGs: metabolism-related genes; GO: Gene Ontology; KEGG: Kyoto Encyclopedia of Genes and Genomes.

A total of 1980 MRGs were identified from the Molecular Signature Database (MSigDB; http://www.broad.mit.edu/gsea/msigdb/, accessed on 20 November 2022; Supplementary File S1). We initially analyzed the correlation between MRG expression and the abundance of infiltrating M2 macrophages via the Spearman correlation analysis using data from the TCGA-STAD and GSE84437 datasets. As shown in the Venn diagram (Figure 3A), 173 MRGs were found to be associated with M2 macrophage infiltration (cor > 0.1, *p* < 0.05; Supplementary File S3). The co-expression network diagram shows the top 30 related MRGs, including TREM2, OLR1, ASAH1, LIPA, and NOX4 (Figure 3B). 

Next, we explored the molecular characteristics of the 173 M2-macrophage-related MRGs using Gene Ontology (GO) enrichment and Kyoto Encyclopedia of Genes and Genomes (KEGG) analyses. As shown in Figure 3C, the GO analysis revealed that these MRGs are primarily involved in fatty acid metabolism, the generation of precursor metabolites and energy, and organic acid biosynthesis, among others, with regard to the category of biological processes (BPs). The significant enrichment terms with regard to cellular components (CCs) were the oxidoreductase complex, the lysosomal lumen, the vacuolar lumen, etc. Significant enrichment with regard to molecular functions (MFs) included heme binding, tetrapyrrole binding, and iron ion binding, among others. KEGG analysis showed that the pathways primarily enriched included retinol metabolism, glycolysis/gluconeogenesis, and carbon metabolism, among others (Figure 3D). 

### 2.3. Construction and Validation of a Novel Prognostic Model

**Figure 4:**
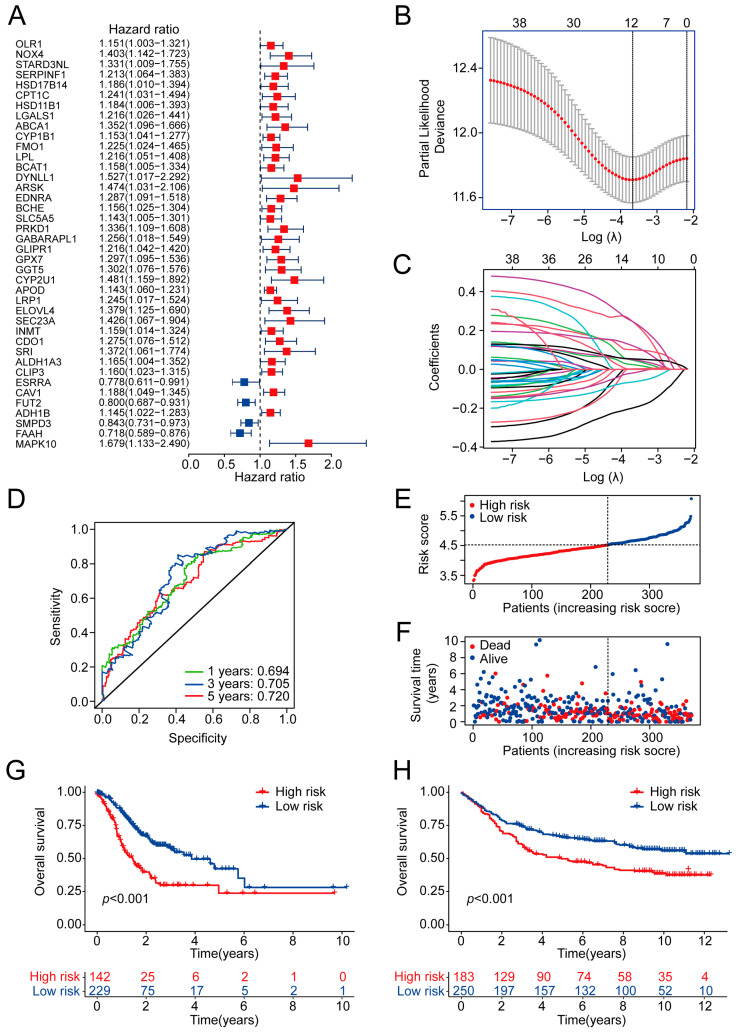
Construction and validation of an MRG prognostic model. (**A**) Forest plot of 40 prognostic M2-macrophage-related MRGs determined via univariate Cox regression analysis. (**B**,**C**) Cvfit and lambda curves showing the process through which we calculated the minimum cross-validation error point (**B**), and the corresponding nonzero coefficients for prognostic risk model construction (**C**). (**D**) 1-, 3-, and 5-year ROC curves of risk model for predicting OS status in the training group. (**E**) Risk plot showing the distribution of the risk score in the training group. (**F**) Risk plot showing the distribution of OS outcome in the training group. (**G**) Kaplan–Meier curves of OS status in the training group. (**H**) Kaplan–Meier curves of OS status in the validation group. MRGs: metabolism-related genes; OS: overall survival; ROC: receiver operator characteristic.

To determine the prognostic value of the MRGs associated with M2 macrophage infiltration, we constructed and validated a prognostic model based on the 173 MRGs. The TCGA-STAD cohort (consisting of 371 samples) was used as the training set, and the GSE84437 cohort (consisting of 433 samples) was used as the validation set. For the training set, we assessed the associations between the MRGs and OS through univariate Cox proportional hazards regression analysis and identified 40 OS-related MRGs (OS-MRGs, *p* < 0.05) (Figure 4A). Lasso regression was then applied to exclude overfitting genes (Figure 4B,C). As a result, 12 OS-MRGs were identified: NOX4, STARD3NL, ABCA1, LPL, DYNLL1, SLC5A5, APOD, ELOVL4, SRI, FUT2, SMPD3, and FAAH. The risk scores of each sample were calculated based on the 12 identified OS-MRGs (the details are shown in Supplementary File S4). The receiver operator characteristic (ROC) curves and corresponding AUC values were also determined to assess the discriminatory ability of the constructed model. The AUC values for the one-year, three-year, and five-year OS were 0.694, 0.705, and 0.72, respectively, suggesting that the sensitivities and specificities of the risk scores were reasonable (Figure 4D). A cutoff point of 4.52 for the model was selected based on the minimum Akaike information criterion (AIC) (Figure S1A). Accordingly, 229 samples (risk score < 4.52) and 142 samples (risk score ≥ 4.52) were classified into the low-risk group and high-risk group, respectively (Figure 4E). Higher risk scores corresponded with more death events (Figure 4F). Kaplan–Meier survival analysis revealed that the patients in the low-risk group had significantly better prognostic outcomes than the patients in the high-risk group (*p* < 0.001) (Figure 4G).

To further evaluate the predictive efficacy of the risk model, the validation set was used to generate a distribution plot of the risk score and the Kaplan–Meier survival curves. As expected, the patients in the validation set could be reasonably divided into low- or high-risk groups according to the cutoff point defined in the TCGA-STAD cohort (Figure S1B,C). Patients with a high risk score had higher mortality rates than patients with a low risk score in the validation set. Notably, the prognostic model has an extremely significant *p*-value (*p* < 0.001) in the validation set, thus confirming that the model has good predictive ability and a wide range of applications (Figure 4H). 

### 2.4. Evaluation of the Clinical Values of the Prognostic Risk Model

**Figure 5:**
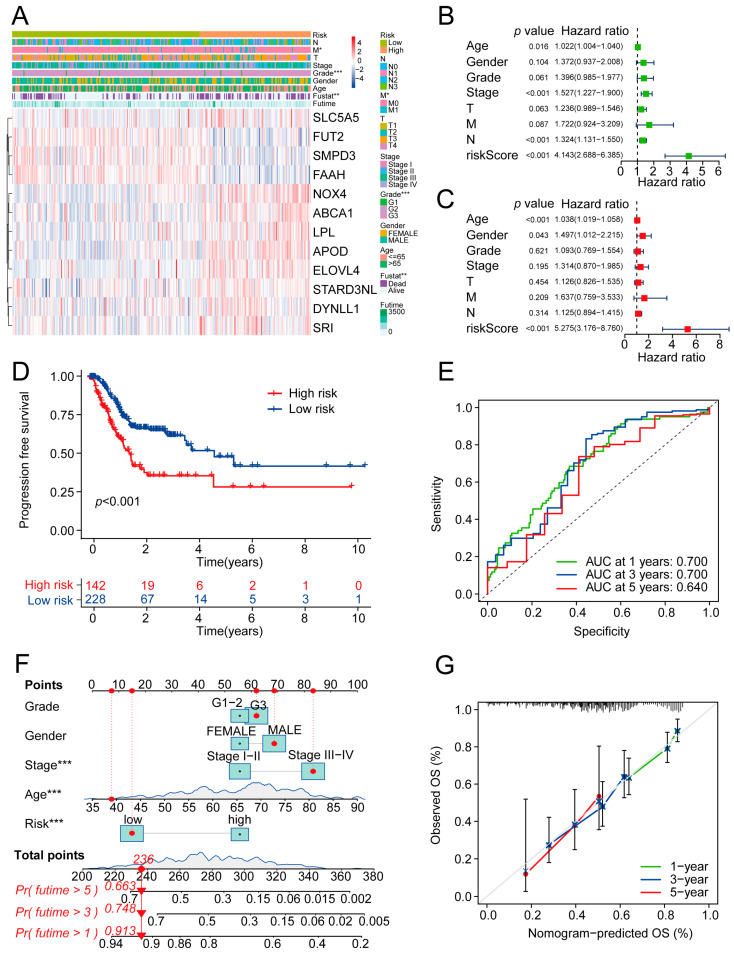
Evaluation of the clinical values of the prognostic risk model. (**A**) The upper heatmap depicts the difference in the distribution of clinical characteristics between the two groups. The bottom heatmap shows the distribution of prognostic MRG expression. * *p* < 0.05, ** *p* < 0.01, and *** *p* < 0.001. (**B**,**C**) Forest plots showing the univariate (**B**) and multivariate (**C**) Cox regression analyses of clinical parameters and the risk score. (**D**) Kaplan–Meier curves for PFS outcome of samples from the TCGA-STAD cohort. (**E**) ROC curves of risk model for predicting 1-year, 3-year, and 5-year PFS status of samples from the TCGA-STAD cohort. (**F**) Nomogram for predicting 1-year, 3-year, and 5-year probabilities of OS outcome. (**G**) Calibration curves for assessing accuracy of the nomogram. The dashed diagonal line in grey represents the ideal model. MRGs: metabolism-related genes; PFS: Progression-Free Survival; OS: overall survival; ROC: receiver operator characteristic.

In the prognostic gene signature, nine risk MRGs (NOX4, STARD3NL, ABCA1, LPL, DYNLL1, SLC5A5, APOD, ELOVL4, and SRI) were upregulated in the high-risk group, whereas three MRGs (FUT2, SMPD3, and FAAH) were downregulated in the high-risk group (Figure 5A). The univariate and multivariate Cox proportional hazard regression analyses confirmed that the risk score based on the 12 MRGs was an independent prognostic factor compared to various clinical characteristics (Figure 5B,C).

We further analyzed the differences in the clinical characteristics between the two risk groups. Significant differences were observed regarding the M stage (*p* < 0.05) and grade (*p* < 0.001) between the two groups (Figure 5A). Kaplan–Meier analysis for PFS (Progression-Free Survival) showed that patients in the high-risk group had higher recurrence rates than patients in the low-risk group (*p* < 0.001) (Figure 5D), with reasonable sensitivities and specificities (Figure 5E). 

Using the clinical parameters and risk score, a nomogram model was established for predicting OS. As depicted in Figure 5F, the one-year, three-year, and five-year predicted OS values of patients were calculated according to the sum of each point. A higher point total corresponded with a worse survival outcome. The calibration curve was used to evaluate this nomogram. The calibration curves based on the prediction of one-year, three-year, and five-year OS were closely consistent with an ideal diagonal curve, which suggested that the nomogram was highly precise (Figure 5G). In addition, a nomogram was also developed for predicting PFS with reasonable accuracy (Figure S1D,E).

### 2.5. Gene Mutation and Drug Sensitivity in the Risk Model

**Figure 6:**
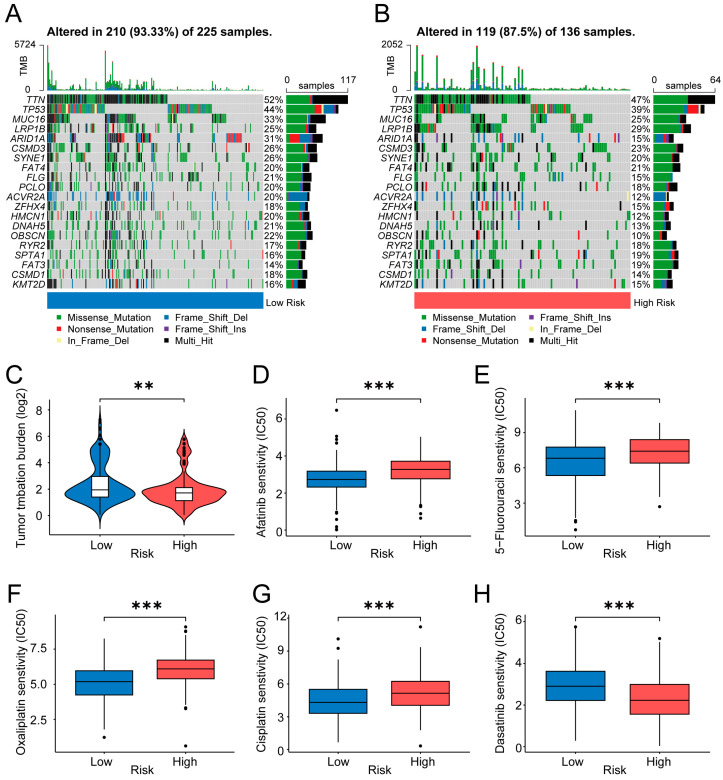
Cancer-related gene mutation and drug sensitivity in the risk model. (**A**,**B**) Waterfall plots showing the mutation information in the low-risk group (**A**) and high-risk group (**B**). (**C**) Violin plot showing the difference in TMB between the two risk groups. (**D**–**H**) Box plots depicting the mean differences in estimated IC50 values of 5 representative drugs (Afatinib, 5-Fluorouracil, Oxaliplatin, Cisplatin, and Dasatinib) between the two risk groups. (**, *p* < 0.01; ***, *p* < 0.001). TMB: tumor mutational burden; IC50: half-maximal inhibitory concentration.

We counted the frequencies and distributions of gene mutations in each group (Figure 6A,B). The waterfall plots show that *TTN* (52%), *TP53* (44%), *MUC16* (33%), *ARID1A* (31%), and *CSMD3* (26%) were the top five most frequent mutational genes in the low-risk group. Moreover, *TTN* (47%), *TP53* (39%), *LRP1B* (29%), *MUC16* (25%), and *CSMD3* (23%) were the top five frequent mutational genes in the high-risk group. In the high-risk group, *ARID1A* had a lower mutational frequency (15% vs. 31%), whereas *FAT3* presented a higher mutational frequency (19% vs. 14%). Analysis of the difference in tumor mutational burden (TMB) between the two groups showed that the TMB values in the low-risk samples were higher than those in the high-risk samples (*p* < 0.01) (Figure 6C).

The above results revealed that risk grouping can help distinguish the mutation of certain genes and determine TMB levels. Mutational signatures are considered markers of drug sensitivity in cancer [20]. Next, we calculated and compared the drug sensitivity (IC50) between the two risk groups. As shown in Figure 6D–G, the commonly used chemotherapeutic and targeted therapeutic drugs analyzed herein, including Afatinib, 5-Fluorouracil, Oxaliplatin, and Cisplatin, may be candidate drugs for patients with low risk scores. Furthermore, Dasatinib may be appropriate for patients with high risk scores (Figure 6H). Significantly, in the 71-drug screening, the low-risk group was more sensitive to 65 types of drugs. The analyses of other drugs are shown in Supplementary File S5.

### 2.6. Estimation of Immune Cell Infiltration and the Immune Response Using the Prognostic Risk Signature

**Figure 7:**
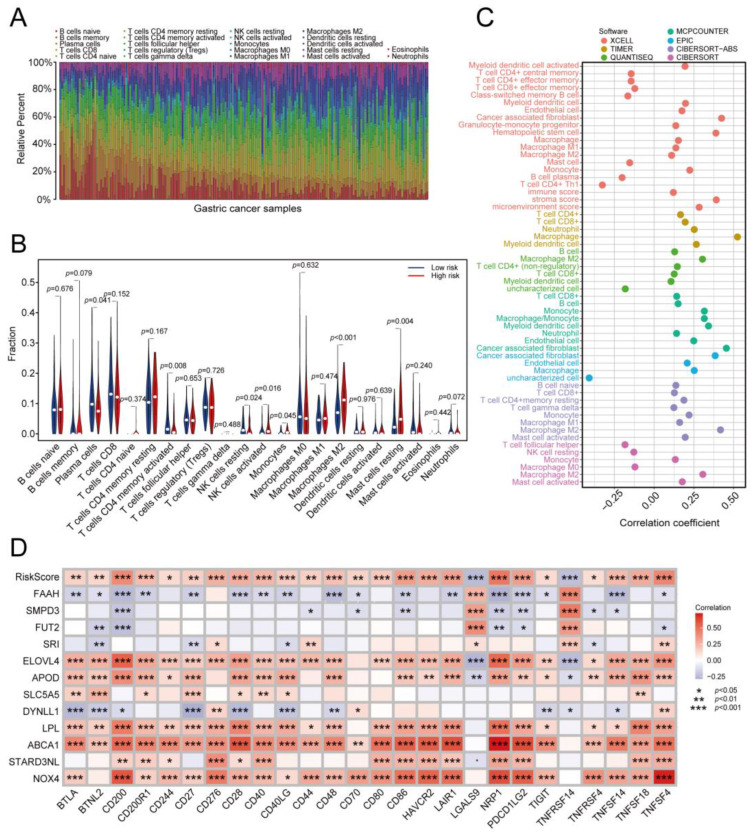
Estimation of immune cell infiltration and immune responses using the prognostic risk signature. (**A**) Bar plot showing the proportion of tumor-infiltrating immune cells in the GC samples from TCGA. (**B**) Violin plots concerning the differences in abundance of immune cells between the two risk groups. (**C**) Correlation coefficients between immune cell proportions and risk scores determined using seven methods. (**D**) Correlation between expression of checkpoint genes and risk scores in the TCGA-STAD cohort. The color of each unit represents the correlation degree. *, *p* < 0.05; **, *p* < 0.01; ***, *p* < 0.001. GC: gastric cancer; TCGA: The Cancer Genome Atlas.

Because the prognostic MRG signature was identified based on M2 macrophage infiltration, we subsequently explored relationships between the prognostic signature and characteristics of the immune microenvironment in GC. First, we calculated the proportions of infiltrating immune cells in the samples using the CIBERSORT method (Figure 7A). We then compared the proportions of each immune cell between the two risk groups. As shown in Figure 7B, some immune cells, including monocytes, M2 macrophages, and resting mast cells, were more enriched in the high-risk group, whereas activated CD4+ memory T cells and resting NK cells were more enriched in the low-risk group. We further calculated the correlation coefficients between the risk score and tumor-infiltrating immune cells using seven methods. Risk scores showed a significant positive association with most immune cells in the TME, such as M2 macrophages, cancer-associated fibroblasts, CD4+ T cells, and CD8+ T cells (Figure 7C). However, risk scores were negatively correlated with some recognized anti-tumor immune cells, such as activated CD4+ and CD8+ effector memory T cells.

High expression levels of immune checkpoint genes can directly inhibit the activation of specific immune cells and contribute to immune escape in cancer cases. Immune checkpoint expression provides clues for evaluating the efficacy of immunotherapies targeting immune checkpoints [21]. We explored the correlation between immune checkpoint gene expression and risk scores. The expression levels of twenty-six checkpoint genes were significantly correlated with the patients’ risk scores. Among these, most genes exhibited positive correlations in terms of expression, such as CD200, CD48, CD80, CD86, and CD276 (*p* < 0.05, Figure 7D). 

Human leukocyte antigen (HLA) genes encode proteins primarily involved in antigen presentation and launch subsequent immune responses [22]. We also found that risk scores were positively correlated with the expression of a series of HLA genes, such as HLA-DOA, HLA-DRA, and HLA-DPA1 (*p* < 0.05) (Figure S2A–L). Overall, risk score severity can be used to evaluate the abundance of infiltrating immune cells and the immune responses of patients.

### 2.7. Single-Cell-Sequencing (scRNA-seq) Analysis of Prognostic Genes

**Figure 8:**
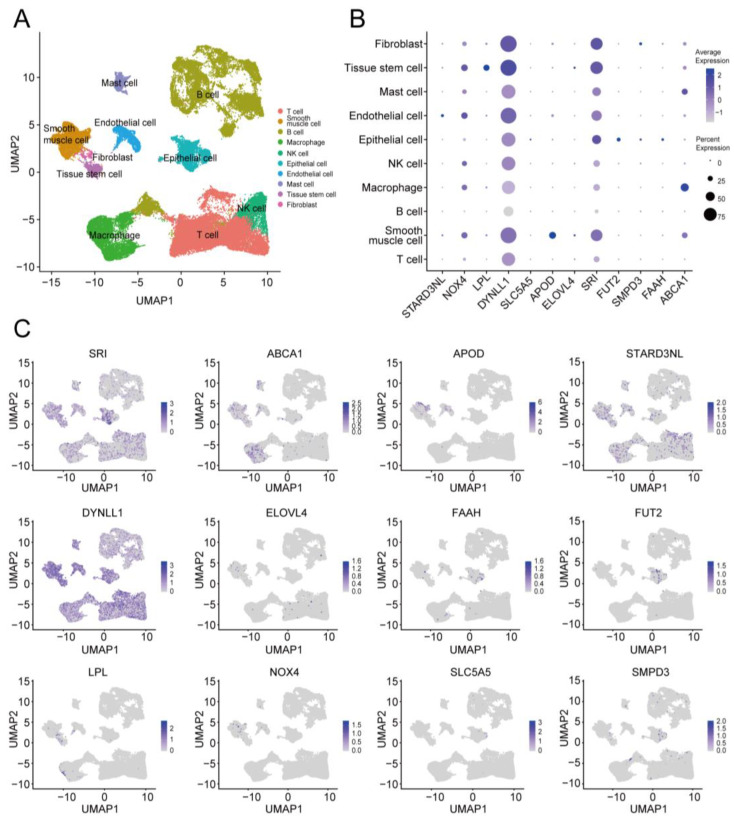
ScRNA-seq analysis of prognostic genes. (**A**) UMAP plot showing annotated cell categories after dimension reduction (GSE183904). (**B**) Dot plot representing relative expression of 12 prognostic genes in the different cell categories. (**C**) UMAP plots showing the distribution of 12 prognostic genes in the single-cell tumor microenvironment atlas. UMAP: Uniform Manifold Approximation and Projection.

We reconstructed a single-cell atlas (GSE183904) comprising 26 GC samples to explore the expression and distribution of the aforementioned MRGs in the TME. After quality control and data merging, 24,907 cells were eventually processed using the Seurat algorithm. We then performed Principal Component Analysis (PCA) dimension reduction followed by clustering and Uniform Manifold Approximation and Projection (UMAP) analysis. All cells were clustered into thirty subgroups (Figure S3A). Cell type annotation was then performed based on previously published studies and using the “Single R” method. The thirty subgroups were further divided into ten categories of cell types, including fibroblasts, tissue stem cells, mast cells, endothelial cells, epithelial cells, macrophages, T cells, B cells, smooth muscle cells, and NK cells (Figure 8A). The expression and distribution of the 12 prognostic genes are depicted in this single-cell atlas (Figure 8B,C). The expression of the gene DYNLL1 was ubiquitous in the TME of GC, whereas SRI, FUT2, and SMPD3 were moderately expressed in GC epithelial cells. Moreover, ABCA1 was primarily expressed in macrophages. 

### 2.8. Experimental Validation of SRI Function in the GC Immune Microenvironment

Based on the aforementioned analysis, the prognostic MRGs associated with M2 macrophage infiltration may be critical targets for ameliorating the outcomes of patients with GC. Bulk RNA sequence (RNA-seq) and scRNA-seq profiling revealed that among the MRGs, SRI was significantly overexpressed in GC cells (Figure 8B,C and Figure S3B). High SRI expression indicated a poor prognosis for GC in both The Cancer Genome Atlas (TCGA) and Gene Expression Omnibus (GEO) datasets (Figure S3C,D). Subsequently, we examined whether the SRI in GC cells regulated the M2 polarization of macrophages in the TME, which accelerates cancer progression. 

**Figure 9:**
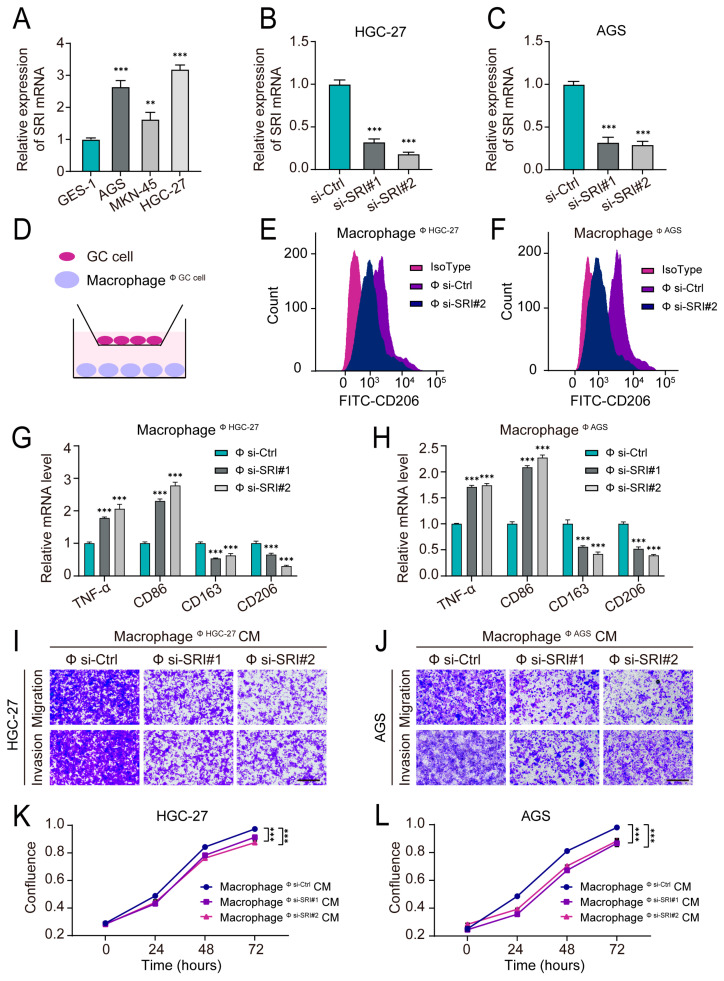
Experimental validation of the role of SRI in the GC immune microenvironment. (**A**) qRT-PCR analysis of SRI mRNA expression in GC cell lines (AGS, HGC-27, and MKN-25) and GES-1 cell lines. (**B**,**C**) qRT-PCR analysis of relative SRI mRNA expression levels after transfection with siRNA in HGC-27 (**B**) and AGS (**C**) cell lines. (**D**) Schematic diagram illustrating the design of a co-culture system for THP-1-derived macrophages and GC cells. Φ symbol represents co-culturing conditions. (**E**,**F**) Flow cytometry analysis of M2 macrophages (CD206 as a marker) after co-culturing with control or SRI-knockdown GC cells for 48 h. (Φ: co-culturing conditions; Φ si-Ctrl: Control; Φ si-SRI#2: co-cultured with SRI-siRNA#2-infected GC cells). (**G**,**H**) Relative mRNA expression of TNF-α, CD86, CD206, and CD163 in THP-1-derived macrophages detected via qRT-PCR after being co-cultured with control or SRI-knockdown GC cells. (Φ: co-culturing conditions; Φ si-Ctrl: Control; Φ si-SRI#1: co-cultured with SRI-siRNA#1-infected GC cells; Φ si-SRI#2: co-cultured with SRI-siRNA#2-infected GC cells). (**I**,**J**) Transwell assays showed the migratory and invasive potential of GC cells cultured in corresponding CM from treated macrophages. Scale bar: 100 μm. (**K**,**L**) Incucyte live-cell-imaging analysis showed the cell proliferation potential of GC cells cultured in corresponding CM from treated macrophages. Data represent findings from three independent experiments and are shown as the mean ± SD of three replicates (**, *p* < 0.01; ***, *p* < 0.001). GC: gastric cancer; CM: conditioned medium.

We first evaluated the expression level of SRI in normal gastric epithelial cells and GC cell lines. It was determined that SRI expression was relatively high in the GC cells, especially in AGS and HGC-27 (Figure 9A). Then, we silenced SRI expression using specific siRNAs in these two cell lines and verified the high silencing efficiency (Figure 9B,C). Control or SRI-knockdown GC cells were co-cultured with PMA-induced THP-1 macrophages in a transwell system (Figure 9D). Flow cytometry analysis revealed that the proportion of M2 macrophages (CD206 as a marker) was significantly reduced after co-culturing with SRI-knockdown GC cells (Figure 9E,F). The qRT-PCR results also showed that SRI-depleted GC cells enhanced the expression levels of M1 biomarkers (TNF-α and CD86) and downregulated M2 biomarker expression (CD163 and CD206) in macrophages (Figure 9G,H). This indicated an important role of SRI in promoting macrophage polarization to the M2 phenotype.

Furthermore, we investigated whether SRI-mediated macrophage polarization to the M2 phenotype contributed to cancer progression. After the co-culturing of macrophages and SRI- knockdown/control GC cells, the conditioned medium (CM) was collected from the treated macrophages and then cultured with GC cells. The CM from the macrophages cultured with the SRI-knockdown groups inhibited the proliferation, migration, and invasion of GC cells (Figure 9I–L). These findings indicate that the knockdown of SRI in GC cells inhibited the M2 polarization of macrophages in the TME, thereby suppressing malignant GC progression.

## 3. Discussion

An increasing number of studies have recognized that metabolic reprogramming in the TME drives the M2 polarization of TAMs, thereby accelerating cancer development [23,24,25]. However, the MRGs that influence M2 macrophage infiltration and their clinical value remain unknown. Large-scale transcriptome data and recently developed computational tools can help to explore these questions. To the best of our knowledge, this study is the first to identify MRGs associated with M2 macrophages using in-depth transcriptome analysis. Notably, based on these MRGs, we developed a novel prognostic signature with promising clinical applications.

Our results indicate that the infiltration level of M2 macrophages is a negative prognostic factor with respect to GC [26]. Interestingly, differences in multiple metabolic pathways were observed between the high- and low-infiltration groups. Previous studies have reported the construction of prognostic models using metabolism-related characteristics. For example, after analyzing RNA-seq data and corresponding clinical data from a GC cohort, Luo et al. identified a group of survival-related MRGs, based on which an effective prognostic model was constructed for risk stratification in relation to GC [27]. Chen et al. divided patients with hepatocellular carcinoma into three energy metabolism-related subtypes with different prognoses. Among the subtypes, further analysis indicated differently expressed energy MRGs, which were subsequently used to establish an effective prognostic model [28]. Although recent studies have identified some prognostic MRGs of cancer, most of these studies have not considered the TME factor in their models and have thus barely revealed the important mechanistic links between an immunosuppressive TME and prognosis. In our study, the prognostic MRG model not only showed good reliability with respect to predicting patient outcomes but also provided novel insights into the evolution of the TME from the perspective of M2 macrophage infiltration. The MRGs associated with M2 macrophage infiltration were identified using both TCGA and GEO datasets. As expected, the constructed risk score showed a significant positive correlation with the infiltration of M2 macrophages. Risk scores were also positively correlated with the infiltration of CD4+ T cells and CD8+ T cells, whereas they were negatively correlated with the infiltration of anti-tumor CD4+ effector memory T cells and CD8+ effector memory T cells in the TME. As M2 macrophages exert strong immunosuppressive effects [29], we can reasonably speculate that the increasing abundance of immune cells does not overrule the effect of the immunosuppressive phenotypes of immune cells [30]. The prognostic MRGs manipulated M2 macrophage infiltration, thereby leading to the development of an immunosuppressive TME and increasing prognostic risk. Further analysis revealed that the patients’ risk scores were positively associated with the expression of certain immune checkpoint proteins. This also suggests that a higher prognostic risk indicates a significantly immunosuppressive TME. 

Commonly used anti-cancer drugs, including platinum-based agents, often target DNA synthesis or DNA damage repair. Thus, mutational signatures in cancer are considered potential markers of drug sensitivity. Our results indicated significant gene mutation frequency and TMB value differences between the two risk groups. In addition, the risk grouping effectively predicted the patients’ sensitivity to multiple targeted drugs and chemotherapeutic agents. The patients in the low-risk group were more sensitive to most therapeutic drugs (such as Afatinib, 5-Fluorouracil, Oxaliplatin, and Cisplatin). This finding also explained why patients with advanced GC exhibit limited responses to conventional drug therapy. The drugs for which the high-risk group exhibited good sensitivity may be candidates for the treatment of patients with advanced GC. In addition, emerging evidence shows that GC patients with high TMB values tend to respond better to immune checkpoint blockade therapy such as anti-PD1 therapy [31,32]. A greater number of mutations in tumor cells provide a greater number of new antigens that can be recognized and targeted by the immune system [33]. Our risk model effectively distinguished between TMB levels and may also help predict the efficacy of immunotherapy with respect to GC. 

The study used bulk RNA-sea and scRNA-sea analyses to determine the molecular characteristics of the crucial prognostic MRGs, and in vitro experiments were conducted to verify them further. We focused on the SRI gene as an example. SRI is highly expressed in certain cancer cells. The TCGA data analyses demonstrated that SRI was highly expressed in GC tissues, and patients with high SRI expression had a poor prognosis. The ScRNA-seq results further indicated that SRI is expressed at high levels in GC-derived epithelial cells. Currently, only a limited number of studies have reported that SRI inhibits cancer cell apoptosis, thereby contributing to the development of GC [34]. Additionally, the role of SRI in the TME has not been revealed. Analysis based on RNA-seq data showed that M2 macrophage infiltration is associated with SRI expression in GC tissue. TAMs are highly plastic and can be induced into the M2 phenotype by tumor cells [4]. We speculated whether the high expression of SRI in GC cells induces the M2 polarization of macrophages in the TME of GC. Subsequent cell experiments confirmed this speculation. In terms of metabolic function, SRI protein can bind to PPP1R3G to form a complex, thus contributing to the regulation of glucose homeostasis and lipid metabolism [35]. The interaction between SRI expression and the M2 polarization of macrophages supports the notion that there is close dependence between GC cell metabolism and immune response in the TME. The prognostic MRGs identified in this study may represent promising targets for future investigations on immunotherapy, leading to novel insights that are yet to be fully elucidated.

Our findings were based on large-scale bulk RNA-seq and scRNA-seq cohorts. Bulk RNA-seq is a powerful and extensively used tool with regard to tumor biomarker discoveries, disease diagnosis, and optimizing treatment [36,37]. Significant infiltration of the stroma and immune cell populations in a tumor results in a highly heterogeneous TME, whereas heterogeneity is only limitedly revealed by bulk RNA-seq. This problem catalyzed the recent development of scRNA-seq. ScRNA-seq has unprecedented utility in revealing the intra-tumor heterogeneity at a single-cell resolution in multiple cancers, including GC [38]. However, scRNA-seq data can only be used to obtain a few thousand types of transcripts from a single cell, which is far fewer than a bulk RNA-seq profile. ScRNA-seq and bulk RNA-seq data cannot provide spatial information, thereby limiting the understanding of pathological changes. Due to these limitations, the intra-tumor immune microenvironment could not be completely assessed. Spatial RNA sequencing (spRNA-seq) is emerging as a transformative technology. SpRNA-seq combines bulk transcriptome analysis and in situ hybridization, thus providing RNA-seq data with spatial locations. Understanding such complex spatial information helps us tremendously with respect to clarifying how cells communicate with each other and how differently expressed genes relate to immune and pathological features [39]. spRNA-seq is still in its infancy, and the future goal is to develop an applicable spatial multiomics technology at single-cell resolution [40]. Owing to the capacity of spRNA-seq to dissect intercellular subpopulations of the TME sensitively and spatially, future studies should be able to elucidate a more detailed mechanism by which metabolic reprogramming closely interacts with the macrophage population and macrophage plasticity in the TME.

## 4. Materials and Methods

### 4.1. Data Acquisition

RNA-seq data and corresponding clinical information were obtained from the STAD cohort in The Cancer Genome Atlas (TCGA) database (https://portal.gdc.cancer.gov/; accessed on 13 November 2022) (*n* = 371) and the GSE84437 dataset (*n* = 433) in the Gene Expression Omnibus (GEO) public database (https://www.ncbi.nlm.nih.gov/geo; accessed on 13 November 2022). The scRNA-seq data were downloaded from the GSE183904 dataset (*n* = 26; accessed on 28 November 2022) in the GEO. The abovementioned datasets complied with the access rules of TCGA and GEO data.

### 4.2. Identification of M2 Macrophage Infiltration

The CIBERSORT method was adopted to calculate the proportions of M2 macrophage infiltration in each sample using the “CIBERSORT” package [41]. CIBERSORT is a deconvolution algorithm that can transform gene expression matrix data into a corresponding immune cell infiltration matrix. The standard *p*-value of <0.05 was used to filter samples.

### 4.3. Gene Set Enrichment Analysis (GSEA) and Gene Set Variation Analysis (GSVA)

GSEA and GSVA were applied to evaluate pathways associated with M2 macrophage infiltration based on the KEGG and GO gene sets from the MSigDB (*p* < 0.05). All analyses were performed using R software (ver 4.2.1) (University of Auckland, Auckland, New Zealand), and the R packages utilized in these operations included the org.Hs.eg.db, clusterProfiler, enrichplot, GSEABase, GSVA, and limma packages. 

### 4.4. Identification of MRGs Associated with M2 Macrophage Infiltration

A total of 1980 MRGs (including glucose-, lipid-, ATP-, and amino-acid-metabolism-related genes) were obtained from the MSigDB [42,43]. The detailed genes are listed in Supplementary File S1. Then, the Spearman method was used to calculate correlation coefficients between MRG expression and the abundance of infiltrating M2 macrophages in the TCGA-STAD and GSE84437 datasets, and all the MRGs associated with M2 macrophage infiltration were identified (|cor| > 0.1 and *p* < 0.05).

The R packages used in these operations were the tidyverse, ggplot2, ggpubr, and ggExtra packages.

### 4.5. Functional Enrichment Analysis

GO and KEGG analyses were performed to explore the functional categories of the identified MRGs (*p* < 0.05). The R packages used in these operations were ggplot2, org.Hs.eg.db, circlize, RColorBrewer, dplyr, ComplexHeatmap, clusterProfiler, and enrichplot.

### 4.6. Construction and Validation of Prognostic Models

Univariate Cox regression analysis was performed to determine OS-MRGs. OS-MRGs were screened using Lasso regression to minimize prediction errors. The best log lambda value (corresponding to the minimum cross-validation error point) was calculated for model fitting. The final OS-MRGs were identified as the prognostic signature with β values using multivariate Cox regression, and the risk score of each patient was calculated using following the formula:$\;\sum_{i=1}^{n}\beta$I × PSI. The one-, three-, and five-year ROC curves were generated to evaluate the accuracy of the prognostic model. The AIC value (the point on the one-year ROC curve that was closest to the upper-left corner) was considered the cut-off point for dividing patients into high- or low-risk groups. Kaplan–Meier curves of survival and progression-free survival (PFS) were constructed to visualize differences between the two risk groups. Furthermore, a nomogram was constructed to predict the OS and PFS of patients. All analyses were performed using R (University of Auckland, Auckland, New Zealand; ver 4.2.1), for which the following packages were employed: the survival, glmnet, survival, timeROC, survminer, rmda, and rms packages.

Univariate and multivariate Cox regression analyses were used to evaluate the independence of the risk scores, and the results were shown in forest plots. The Chi-square test and Wilcoxon signed-rank test were conducted to analyze the relationship between the risk score and clinicopathological characteristics, and a band diagram was used for visualization. The R packages used in this respect were survival, forestplot, and pheatmap.

### 4.7. Identification of Mutated Genes and Drug Sensitivity Prediction

The Chi-square test and waterfall plots were performed and developed to analyze and visualize the frequency and distribution of gene mutations in each group, respectively (*p* < 0.05). The R package maftools was used to generate the waterfall plots. 

We calculated the half inhibitory concentration (IC50) of common chemotherapies in the two risk groups and showed differences between groups in the form of box plots by performing the Wilcoxon signed-rank test. The R packages used in this procedure include oncoPredict, parallel, limma, ggplot2, and ggpubr.

### 4.8. Assessment of Immune Cell Infiltration and Immune Microenvironment

We used seven methods (TIMER, CIBERSORT, XCELL, QUANTISEQ, MCP-counter, EPIC, and CIBERSORT-ABS) to evaluate the abundance of immune cell infiltration among the samples in the TCGA–STAD dataset. The correlation between the risk score and the proportion of each immune cell was evaluated using the Spearman correlation analysis. A lollipop diagram was constructed to present the correlation coefficients (*p* < 0.05). Furthermore, linear correlation plots were developed to indicate the correlation between HLA gene expression and risk scores via the Spearman correlation analysis. The R packages involved in this regard were corrplot, scales, limma, ggplot2, and ggtext.

### 4.9. ScRNA-seq Analysis of Prognostic Genes

The “Seurat” R Package (https://satijalab.org/seurat/; accessed on 28 November 2022; version 4.2.1) was used to analyze the scRNA-seq data. ScRNA-seq data were filtered using the following criteria: 1000–6000 unique gene counts, <5% mitochondrial counts, and >300 genes. The “scTransform” and “harmony” methods were used for batch correction and subsequent data integration, respectively. Then, principal component analysis (PCA), cluster analysis, and uniform manifold approximation and projection (UMAP) for dimensionality reduction and visualization purposes were used for the integrated dataset. The top 30 principal components were selected, and a resolution of 0.8 was adopted for clusters. Clusters were annotated according to known marker genes from previously published studies and the “SingleR” method [44]. The R packages used in this regard included Seurat, dplyr, tidyverse, patchwork, Matrix, scales, cowplot, harmony, data.table, stringr, and SingleR.

### 4.10. Cell Culture and Transfection

THP-1 cells were provided by the American Type Culture Collection (ATCC, Manassas, VA, USA). The Cell Resource Center of the Chinese Academy of Medical Sciences (Beijing, China) provided AGS, MKN-45, and HGC-27 cells. For in vitro investigations, RPMI-1640 (Gibco, Carlsbad, CA, USA) with 10% fetal bovine serum (Gibco) and 1% penicillin–streptomycin solution were utilized to culture cells at 37 °C with 5% CO_2_. Two distinct small interfering RNAs (siRNA) against SRI were designed and synthesized by Genepharma (Suzhou, China). The target sequences of siRNA for SRI were 5′-GAUCCGCUGUAUGGUUACUTT-3′ (si-SRI#1) and 5′-GCCGGCUUAUGGUUUCAAUTT-3′ (si-SRI#2). All transfections were performed according to the manufacturer’s protocols using jetPRIME (Polyplus Transfection, New York, NY, USA).

### 4.11. RNA Extraction and Quantitative Real-Time Polymerase Chain Reaction (qRT–PCR)

Total cellular RNA was extracted from cell lines using Total RNA Extraction Reagent (R0027, Beyotime, Shanghai, China) following the standard protocol. The obtained RNA was subsequently processed for cDNA using a cDNA synthesis kit (R233-01, Vazyme, Nanjing, China). The cDNA was then subjected to real-time quantitative reverse transcription–polymerase chain reaction (qRT–PCR) assay on a CFX96 (Bio-Rad, Hercules, CA, USA) using qPCR SYBR Green Master Mix (Q111-03, Vazyme, China). Relative mRNA expression was calculated using the 2−ddCt method and normalized to GAPDH. All primers were synthesized by Sangon Biotech (Shanghai, China) and are listed in Supplementary File S2.

### 4.12. Macrophage Polarization Experiments

THP-1 cells were treated with PMA (Sigma-Aldrich, St. Louis, MO, USA) for 24 h to induce macrophage-like differentiation. The THP-1-derived macrophages were co-cultured with GC (HGC-27 or AGS cells) in a co-culture transwell system (Corning, New York, NY, USA). GC cells were placed in the lower chamber, and macrophages were placed in the upper chamber. Co-cultured macrophages were harvested after 48 h.

### 4.13. Flow Cytometry

Macrophages were collected and washed twice using washing buffer (PBS with 1% FBS). Macrophages were fixed with 4% paraformaldehyde at room temperature for 10 min and blocked with 1% BSA in PBS for 1 h at room temperature. Macrophages were then incubated with FITC anti-human CD206 Antibody (#321103; Biolegend, San Diego, CA, USA) for 30 min at room temperature. Then, macrophages were analyzed using BD LSRFortessa (BD Biosciences, Franklin Lakes, NJ, USA) and Flowjo software (Tree Star, Inc., Ashland, OR, USA).

### 4.14. Cell Migration and Invasion Assay

GC cells were collected and resuspended in a serum-free medium. Cells were then plated on the upper chamber of the transwell system (Corning, New York, NY, USA). For invasion assays, the upper chamber was pre-coated with Matrigel (#350234; Corning, New York, NY, USA; 1:8 diluted with RPMI-1640). Cell growth medium (20% FBS) was added in the chambers of 24-well plates. After incubating for 24 h (migration assay) or 48 h (invasion assay), cells were fixed with 4% paraformaldehyde and stained with 0.1% crystal violet solution (G1063, Solarbio, Beijing, China). Stained cells were visualized and recorded under a microscope.

### 4.15. Incucyte Live-Cell-Imaging Analysis

Cell proliferation was measured by performing live-cell-imaging analysis (IncuCyte ZOOM™; Essen Bioscience, Ann Arbor, MI, USA). Briefly, 5000 cancer cells were plated onto a 96-well clear bottom plate. Nine images were captured in every well at the specified time point. Cell proliferation was determined by calculating the total area occupied by cells (% confluence).

## 5. Conclusions

In summary, this study is the first to evaluate the prognostic and applicational value of MRGs with regard to cancer from the perspective of M2 macrophage infiltration. We constructed and validated a convincing prognostic model based on independent training and validation datasets. The prognostic signature identified herein can reflect the immune microenvironment of patients to some extent and can be applied to evaluate patients who are more likely to benefit from cancer therapeutics. Our results highlight the existence of a significant correlation between metabolic reprogramming and M2 macrophage infiltration, thus providing a novel prognostic signature and promising therapeutic targets for GC. 

## Data Availability

The original data presented in the study can be found in the online repositories (listed in the article). The data are also available from the corresponding author.

## Supplementary Materials

The following supporting information can be downloaded at: https://www.mdpi.com/article/10.3390/ijms241310625/s1.

## Abbreviations

MRGs: metabolism-related genes; GC: gastric cancer; TNM: tumor node metastasis; TME: tumor microenvironment; TAMs: tumor-associated macrophages; SRI: Sorcin; STAD: stomach adenocarcinoma; TCGA: The Cancer Genome Atlas; GEO: Gene Expression Omnibus; RNA-seq: RNA-sequence; scRNA-seq: single-cell sequencing; GSEA: Gene Set Enrichment Analysis; GSVA: Gene Set Variation Analysis; KEGG: Kyoto Encyclopedia of Genes and Genomes; GO: Gene Ontology; MSigDB: Molecular Signature Database; OS-MRGs: OS-related MRGs; ROC: receiver operator characteristic; AIC: Akaike information criterion; OS: overall survival; PFS: Progression-Free Survival; PCA: Principal Component Analysis; UMAP: Uniform Manifold Approximation and Projection; qRT–PCR: Quantitative Real-Time Polymerase Chain Reaction; BPs: biological processes; CC: cellular component; MFs: molecular functions; AUC: area under the curve; TMB: tumor mutational burden; HLA: Human leukocyte antigen; IC50: half-maximal inhibitory concentration; CM: conditioned medium.

## References

[1] Sung H., Ferlay J., Siegel R.L., Laversanne M., Soerjomataram I., Jemal A., Bray F. (2021). Global Cancer Statistics 2020: GLOBOCAN Estimates of Incidence and Mortality Worldwide for 36 Cancers in 185 Countries. CA Cancer J. Clin..

[2] Joshi S.S., Badgwell B.D. (2021). Current treatment and recent progress in gastric cancer. CA Cancer J. Clin..

[3] Chen D., Fu M., Chi L., Lin L., Cheng J., Xue W., Long C., Jiang W., Dong X., Sui J. (2022). Prognostic and predictive value of a pathomics signature in gastric cancer. Nat. Commun..

[4] Cassetta L., Pollard J.W. (2018). Targeting macrophages: Therapeutic approaches in cancer. Nat. Rev. Drug Discov..

[5] Orecchioni M., Ghosheh Y., Pramod A.B., Ley K. (2019). Macrophage Polarization: Different Gene Signatures in M1(LPS+) vs. Classically and M2(LPS-) vs. Alternatively Activated Macrophages. Front. Immunol..

[6] Goswami K.K., Bose A., Baral R. (2021). Macrophages in tumor: An inflammatory perspective. Clin. Immunol..

[7] Chen Y., Zhang S., Wang Q., Zhang X. (2017). Tumor-recruited M2 macrophages promote gastric and breast cancer metastasis via M2 macrophage-secreted CHI3L1 protein. J. Hematol. Oncol..

[8] Vitale I., Manic G., Coussens L.M., Kroemer G., Galluzzi L. (2019). Macrophages and Metabolism in the Tumor Microenvironment. Cell Metab..

[9] Li W., Wu F., Zhao S., Shi P., Wang S., Cui D. (2022). Correlation between PD-1/PD-L1 expression and polarization in tumor-associated macrophages: A key player in tumor immunotherapy. Cytokine Growth Factor Rev..

[10] Pittet M.J., Michielin O., Migliorini D. (2022). Clinical relevance of tumour-associated macrophages. Nat. Rev. Clin. Oncol..

[11] Yang P., Qin H., Li Y., Xiao A., Zheng E., Zeng H., Su C., Luo X., Lu Q., Liao M. (2022). CD36-mediated metabolic crosstalk between tumor cells and macrophages affects liver metastasis. Nat. Commun..

[12] Saravia J., Chapman N.M., Chi H. (2019). Helper T cell differentiation. Cell. Mol. Immunol..

[13] Martínez-Reyes I., Chandel N.S. (2021). Cancer metabolism: Looking forward. Nat. Rev. Cancer.

[14] Xia L., Oyang L., Lin J., Tan S., Han Y., Wu N., Yi P., Tang L., Pan Q., Rao S. (2021). The cancer metabolic reprogramming and immune response. Mol. Cancer.

[15] Chen M., Zhang J., Sampieri K., Clohessy J.G., Mendez L., Gonzalez-Billalabeitia E., Liu X.-S., Lee Y.-R., Fung J., Katon J.M. (2018). An aberrant SREBP-dependent lipogenic program promotes metastatic prostate cancer. Nat. Genet..

[16] Di Conza G., Tsai C.-H., Gallart-Ayala H., Yu Y.-R., Franco F., Zaffalon L., Xie X., Li X., Xiao Z., Raines L.N. (2021). Tumor-induced reshuffling of lipid composition on the endoplasmic reticulum membrane sustains macrophage survival and pro-tumorigenic activity. Nat. Immunol..

[17] Xu Y., Lu J., Tang Y., Xie W., Zhang H., Wang B., Zhang S., Hou W., Zou C., Jiang P. (2022). PINK1 deficiency in gastric cancer compromises mitophagy, promotes the Warburg effect, and facilitates M2 polarization of macrophages. Cancer Lett..

[18] Gambardella V., Castillo J., Tarazona N., Gimeno-Valiente F., Martínez-Ciarpaglini C., Cabeza-Segura M., Roselló S., Roda D., Huerta M., Cervantes A. (2020). The role of tumor-associated macrophages in gastric cancer development and their potential as a therapeutic target. Cancer Treat. Rev..

[19] Weber M., Wehrhan F., Baran C., Agaimy A., Büttner-Herold M., Öztürk H., Neubauer K., Wickenhauser C., Kesting M., Ries J. (2020). Malignant transformation of oral leukoplakia is associated with macrophage polarization. J. Transl. Med..

[20] Levatić J., Salvadores M., Fuster-Tormo F., Supek F. (2022). Mutational signatures are markers of drug sensitivity of cancer cells. Nat. Commun..

[21] Huang A.C., Zappasodi R. (2022). A decade of checkpoint blockade immunotherapy in melanoma: Understanding the molecular basis for immune sensitivity and resistance. Nat. Immunol..

[22] Pujadas E., Cordon-Cardo C. (2021). The human leukocyte antigen as a candidate tumor suppressor. Cancer Cell..

[23] Xia Y., Rao L., Yao H., Wang Z., Ning P., Chen X. (2020). Engineering Macrophages for Cancer Immunotherapy and Drug Delivery. Adv. Mater..

[24] Mehla K., Singh P.K. (2019). Metabolic Regulation of Macrophage Polarization in Cancer. Trends Cancer.

[25] Boutilier A.J., Elsawa S.F. (2021). Macrophage Polarization States in the Tumor Microenvironment. Int. J. Mol. Sci..

[26] Hu J., Ma Y., Ma J., Yang Y., Ning Y., Zhu J., Wang P., Chen G., Liu Y. (2021). M2 Macrophage-Based Prognostic Nomogram for Gastric Cancer After Surgical Resection. Front. Oncol..

[27] Luo T., Li Y., Nie R., Liang C., Liu Z., Xue Z., Chen G., Jiang K., Liu Z.-X., Lin H. (2020). Development and validation of metabolism-related gene signature in prognostic prediction of gastric cancer. Comput. Struct. Biotechnol. J..

[28] Chen Q., Li F., Gao Y., Xu G., Liang L., Xu J. (2020). Identification of Energy Metabolism Genes for the Prediction of Survival in Hepatocellular Carcinoma. Front. Oncol..

[29] Noy R., Pollard J.W. (2014). Tumor-associated macrophages: From mechanisms to therapy. Immunity.

[30] Riera-Domingo C., Audigé A., Granja S., Cheng W.C., Ho P.C., Baltazar F., Stockmann C., Mazzone M. (2020). Immunity, Hypoxia, and Metabolism-the Ménage à Trois of Cancer: Implications for Immunotherapy. Physiol. Rev..

[31] Bai Y., Xie T., Wang Z., Tong S., Zhao X., Zhao F., Cai J., Wei X., Peng Z., Shen L. (2022). Efficacy and predictive biomarkers of immunotherapy in Epstein-Barr virus-associated gastric cancer. J. Immunother. Cancer.

[32] Wahida A., Buschhorn L., Fröhling S., Jost P.J., Schneeweiss A., Lichter P., Kurzrock R. (2023). The coming decade in precision oncology: Six riddles. Nat. Rev. Cancer.

[33] Hegde P.S., Chen D.S. (2020). Top 10 Challenges in Cancer Immunotherapy. Immunity.

[34] Deng L., Tan T., Zhang T., Xiao X., Gu H. (2019). miR-1 reverses multidrug resistance in gastric cancer cells via downregulation of sorcin through promoting the accumulation of intracellular drugs and apoptosis of cells. Int. J. Oncol..

[35] Genovese I., Carotti A., Ilari A., Fiorillo A., Battista T., Colotti G., Ivarsson Y. (2020). Profiling calcium-dependent interactions between Sorcin and intrinsically disordered regions of human proteome. Biochim. Biophys. Acta Gen. Subj..

[36] Stark R., Grzelak M., Hadfield J. (2019). RNA sequencing: The teenage years. Nat. Rev. Genet..

[37] Wang J., Dean D.C., Hornicek F.J., Shi H., Duan Z. (2019). RNA sequencing (RNA-Seq) and its application in ovarian cancer. Gynecol. Oncol..

[38] Jia Q., Chu H., Jin Z., Long H., Zhu B. (2022). High-throughput single-cell sequencing in cancer research. Signal Transduct. Target. Ther..

[39] Li X., Wang C.-Y. (2021). From bulk, single-cell to spatial RNA sequencing. Int. J. Oral. Sci..

[40] Vandereyken K., Sifrim A., Thienpont B., Voet T. (2023). Methods and applications for single-cell and spatial multi-omics. Nat. Rev. Genet..

[41] Newman A.M., Liu C.L., Green M.R., Gentles A.J., Feng W., Xu Y., Hoang C.D., Diehn M., Alizadeh A.A. (2015). Robust enumeration of cell subsets from tissue expression profiles. Nat. Methods.

[42] He Z., Wang C., Xue H., Zhao R., Li G. (2020). Identification of a Metabolism-Related Risk Signature Associated With Clinical Prognosis in Glioblastoma Using Integrated Bioinformatic Analysis. Front. Oncol..

[43] Subramanian A., Tamayo P., Mootha V.K., Mukherjee S., Ebert B.L., Gillette M.A., Paulovich A., Pomeroy S.L., Golub T.R., Lander E.S. (2005). Gene set enrichment analysis: A knowledge-based approach for interpreting genome-wide expression profiles. Proc. Natl. Acad. Sci. USA.

[44] Kumar V., Ramnarayanan K., Sundar R., Padmanabhan N., Srivastava S., Koiwa M., Yasuda T., Koh V., Huang K.K., Tay S.T. (2022). Single-Cell Atlas of Lineage States, Tumor Microenvironment, and Subtype-Specific Expression Programs in Gastric Cancer. Cancer Discov..

