# Supplementary material for: Identification and Validation of a Metabolism-Related Prognostic Signature Associated with M2 Macrophage Infiltration in Gastric Cancer
Source: Int J Mol Sci. 2023 Jun 25;24(13):10625. doi: 10.3390/ijms241310625 (PMC10342140; doi:10.3390/ijms241310625)
Supplement: Supplementary file 1 [file ijms-24-10625-s001.zip › Supplemental figures and figure legends.pdf]

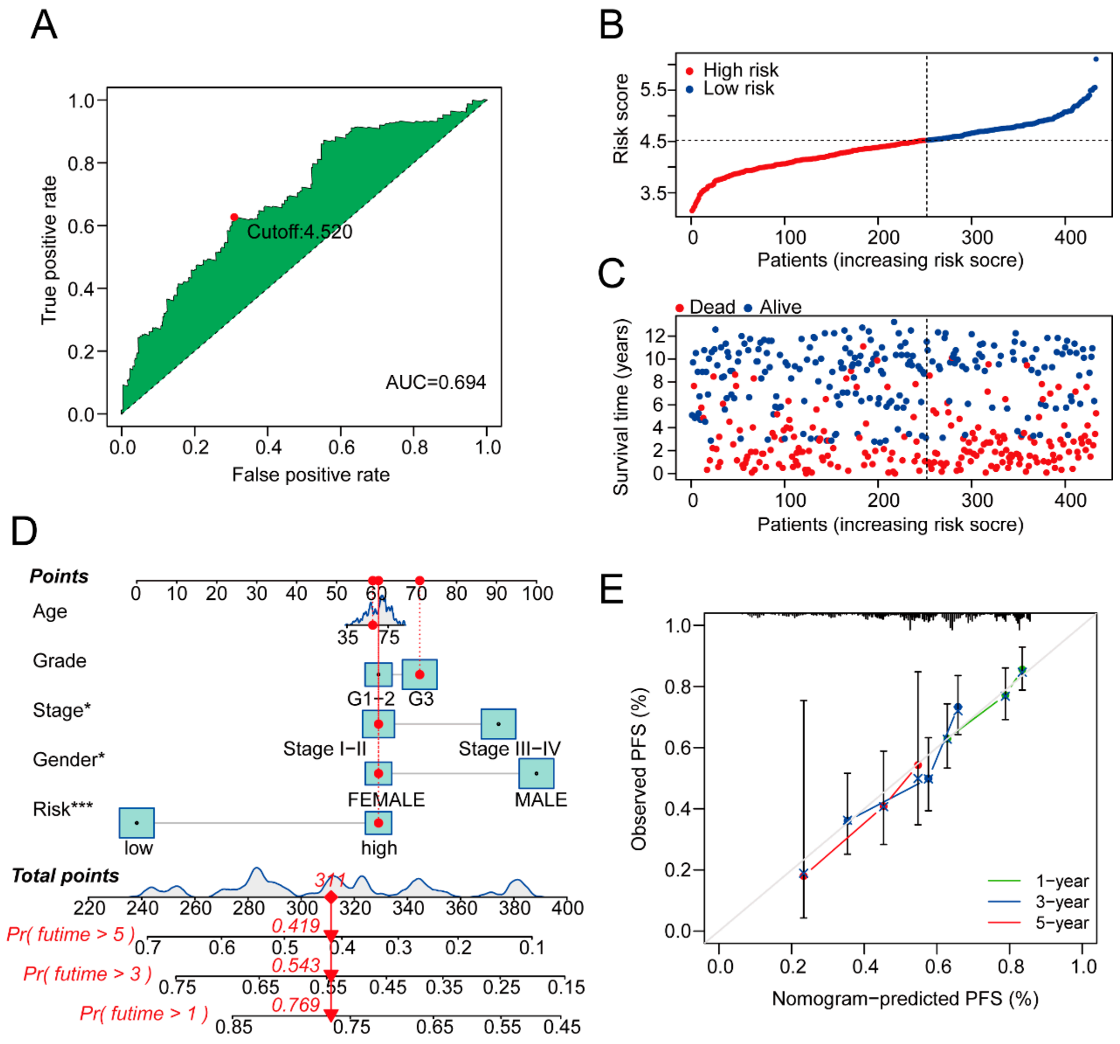

Figure S1. (A) The 1-year ROC curve with the lowest AIC value for the optimal cut-off point of prognostic model. (B) Risk plot showing the distribution of the risk score in the validation group (C) Risk plot showing the distribution of OS outcome in the validation group. (D) Nomogram for predicting 1-year, 3-year, and 5-year probabilities of PFS outcome. (E) Calibration curves for assessing accuracy of the nomogram. The dashed diagonal line in grey colour represents the ideal model. ROC: receiver operator characteristic; AIC: Akaike information criterion; OS: overall survival; PFS: Progression-Free-Survival.

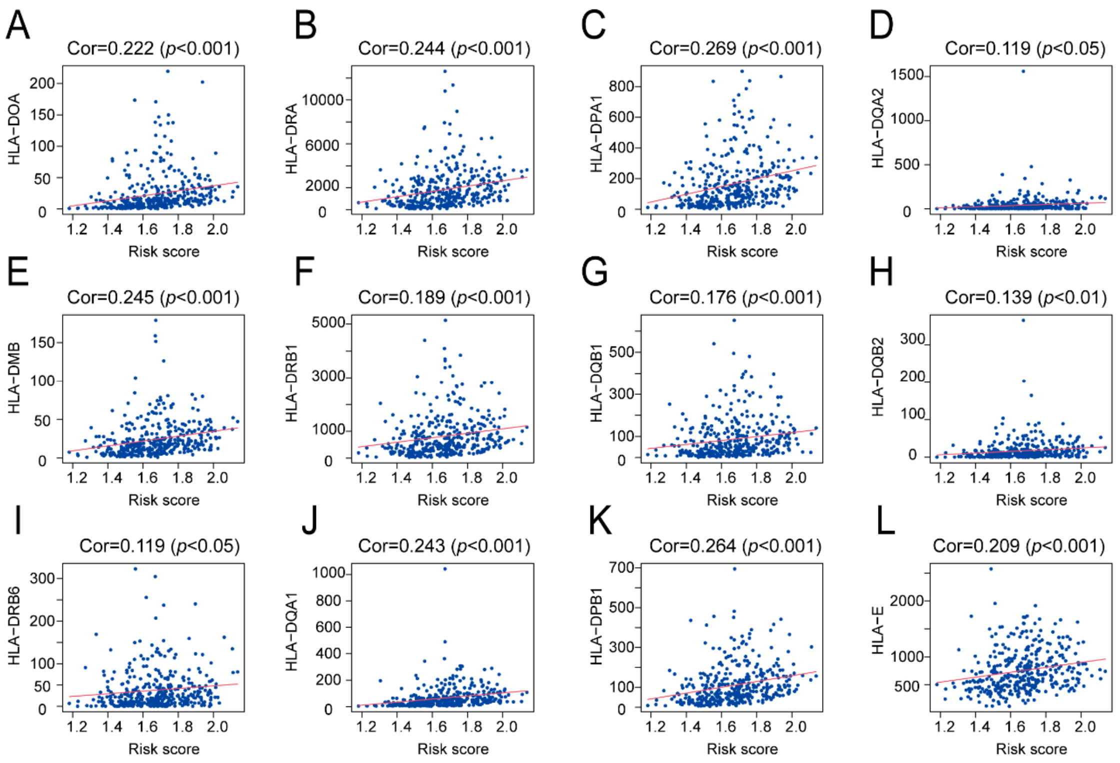

Figure S2. Spearman correlation diagram showing the correlation between the risk score and HLA gene expression. HLA: Human leukocyte antigen.

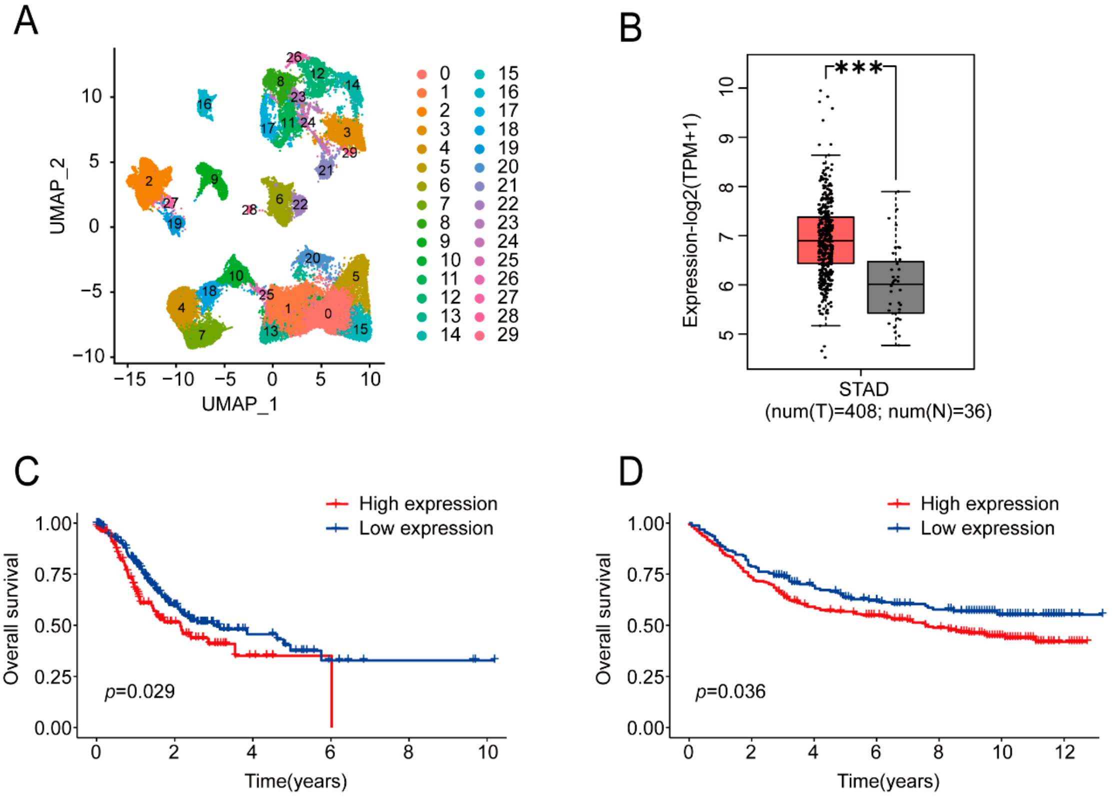

Figure S3. (A) UMAP plot showing cell categories after dimension reduction. (B) Box plot of SRI expression in GC using the GEPIA2 tool (http://gepia2.cancer-pku.cn/#index) (***, p<0.001). (C) Kaplan–Meier curves showed the difference in OS between the high- and low-SRI expression groups in the TCGA-STAD dataset. (D) Kaplan–Meier curves showed the difference in OS between the high- and low-SRI expression groups in the GSE84437 dataset. UMAP: Uniform Manifold Approximation and Projection; GC: gastric cancer; OS: overall survival.
