# Supplementary material for: Identification and Validation of a Metabolism-Related Prognostic Signature Associated with M2 Macrophage Infiltration in Gastric Cancer
Source: Int J Mol Sci. 2023 Jun 25;24(13):10625. doi: 10.3390/ijms241310625 (PMC10342140; doi:10.3390/ijms241310625)
Supplement: Supplementary file 1 [file ijms-24-10625-s001.zip › Supplementary file S1.pdf]

Supplementary file 1
A4GALT
AACS
AADAT
ABCA1
ABCB11
ABCB4
ABCC1
ABCC3
ABCD1
ABHD3
ABHD4
ABHD5
ABO
AC003665.1
AC092724.1
ACAA1
ACAA2
ACACA
ACACB
ACAD10
ACAD11
ACADL
ACADM
ACADS
ACADSB
ACADVL
ACAT1
ACAT2
ACBD4
ACBD5
ACBD6
ACBD7
ACER1
ACER2
ACER3
ACHE
ACLY
ACO2
ACOT1
ACOT11
ACOT12
ACOT13
ACOT2

ACOT4
ACOT6
ACOT7
ACOT8
ACOT9
ACOX1
ACOX2
ACOX3
ACOXL
ACP6
ACSBG1
ACSBG2
ACSF2
ACSF3
ACSL1
ACSL3
ACSL4
ACSL5
ACSL6
ACSM3
ACSM6
ACSS1
ACSS3
ADH1A
ADH1B
ADH1C
ADH4
ADH5
ADH6
ADH7
ADIPOQ
ADIPOR1
ADIPOR2
ADPRM
ADSL
AGK
AGMO
AGPAT1
AGPAT2
AGPAT3
AGPAT4
AGPAT5
AGPS
AGRP

AGT
AHR
AHRR
AKR1A1
AKR1B1
AKR1B15
AKR1C1
AKR1C2
AKR1C3
AKR1C4
AKR1D1
AKT1
AKT2
AKT3
ALAD
ALAS1
ALB
ALDH1A1
ALDH1B1
ALDH2
ALDH3A1
ALDH3A2
ALDH3B1
ALDH3B2
ALDH7A1
ALDH9A1
ALDOA
ALOX12
ALOX12B
ALOX15
ALOX15B
ALOX5
ALOX5AP
ALOXE3
ALPI
AMACR
ANGPTL4
ANKRD1
AOC3
APEX1
APOA1
APOA2
APOA5
APOC3

APOD
AQP7
ARF1
ARF3
ARNT
ARNT2
ARNTL
ARSA
ARSB
ARSD
ARSE
ARSF
ARSG
ARSH
ARSI
ARSJ
ARSK
ARV1
ASAH1
ASAH2
AUH
AWAT1
AWAT2
B3GALNT1
B3GALT1
B3GALT2
B3GALT4
B3GALT5
B3GNT2
B3GNT3
B3GNT4
B3GNT5
B4GALNT1
B4GALT1
B4GALT2
B4GALT3
B4GALT4
B4GALT6
B4GAT1
BAAT
BCHE
BCKDHB
BDH1
BDH2

BLVRA
BMPR1B
BMX
BPHL
CA2
CA4
CA6
CAMKK1
CAMKK2
CARM1
CAV1
CBR1
CBR3
CBR4
CCDC58
CCNC
CD1D
CD36
CDIPT
CDK19
CDK8
CDS1
CDS2
CEL
CEPT1
CERK
CERS1
CERS2
CERS3
CERS4
CERS5
CERS6
CGA
CH25H
CHAT
CHD9
CHKA
CHKB
CHPT1
CHUK
CIDEA
CIDEC
CLOCK
COL4A3BP

COMT
CPNE1
CPNE3
CPNE6
CPNE7
CPOX
CPT1A
CPT1B
CPT1C
CPT2
CPTP
CRAT
CREBBP
CRLS1
CROT
CRYZ
CSNK1G2
CSNK2A1
CSNK2A2
CSNK2B
CTSA
CUBN
CYP11A1
CYP11B1
CYP11B2
CYP17A1
CYP19A1
CYP1A1
CYP1A2
CYP1B1
CYP21A2
CYP24A1
CYP27A1
CYP27B1
CYP2A13
CYP2A7
CYP2B6
CYP2C18
CYP2C19
CYP2C8
CYP2C9
CYP2D6
CYP2E1
CYP2F1

CYP2J2
CYP2R1
CYP2U1
CYP39A1
CYP3A4
CYP3A43
CYP3A5
CYP3A7
CYP46A1
CYP4A11
CYP4A22
CYP4B1
CYP4F11
CYP4F12
CYP4F2
CYP4F22
CYP4F3
CYP4F8
CYP51A1
CYP7A1
CYP7B1
CYP8B1
D2HGDH
DBI
DDHD1
DDHD2
DECR1
DECR2
DEGS1
DEGS2
DGAT1
DGAT2
DGAT2L6
DGKA
DGKB
DGKD
DGKE
DGKG
DGKH
DGKI
DGKQ
DGKZ
DHCR24
DHCR7

DHRS7B
DLD
DLST
DPEP1
DPEP2
DPEP3
EBP
ECH1
ECHS1
ECI1
ECI2
EHHADH
ELOVL1
ELOVL2
ELOVL3
ELOVL4
ELOVL5
ELOVL6
ELOVL7
ENO2
ENO3
ENPP6
ENPP7
EP300
EPHX1
EPHX2
ERP29
ESRRA
ESYT1
ESYT2
ESYT3
ETFDH
ETNK1
ETNK2
ETNPPL
FA2H
FAAH
FAAH2
FABP1
FABP12
FABP2
FABP3
FABP4
FABP5

FABP6
FABP7
FABP9
FADS1
FADS2
FAM120B
FAR1
FAR2
FASN
FDFT1
FDPS
FDX1
FDX2
FDXR
FH
FHL2
FIG4
FITM1
FITM2
FMO1
FUT1
FUT2
FUT3
FUT4
FUT5
FUT6
FUT7
FUT9
G0S2
G6PC
G6PC2
GABARAPL1
GAD2
GAL3ST1
GALC
GAPDHS
GBA
GBA2
GBGT1
GC
GCDH
GCNT2
GDE1
GDPD1

GDPD3
GDPD5
GGPS1
GGT1
GGT5
GGT6
GGT7
GK
GK2
GK3P
GLA
GLB1
GLB1L
GLIPR1
GLTP
GLUL
GLYCTK
GM2A
GNPAT
GPAM
GPAT2
GPAT3
GPAT4
GPCPD1
GPD1
GPD1L
GPD2
GPS2
GPX1
GPX2
GPX3
GPX4
GPX5
GPX6
GPX7
GRHL1
GRHPR
GSTM4
GSTZ1
H2AFZ
HACD1
HACD2
HACD3
HACD4

HACL1
HADH
HADHA
HADHB
HAO2
HCCS
HDAC3
HELZ2
HEXA
HEXB
HIBCH
HILPDA
HMGCL
HMGCLL1
HMGCR
HMGCS1
HMGCS2
HPGD
HPGDS
HSD11B1
HSD11B2
HSD17B1
HSD17B10
HSD17B11
HSD17B12
HSD17B13
HSD17B14
HSD17B2
HSD17B3
HSD17B4
HSD17B6
HSD17B7
HSD17B8
HSD3B1
HSD3B2
HSD3B7
HSDL2
HSP90AA1
HSPH1
HTD2
IDH1
IDH3B
IDH3G
IDI1

IDI2
IKBKB
IKBKG
IL4I1
ILK
INMT
INPP4A
INPP4B
INPP5D
INPP5E
INPP5F
INPP5J
INPP5K
INPPL1
INSIG1
INSIG2
IRS1
IRS2
IRS4
JAK2
JMJD7-PLA2G4B
KDSR
KMT5A
KPNB1
LBR
LCAT
LCLAT1
LCN1
LCN12
LCN15
LCN9
LDHA
LEP
LEPR
LGALS1
LGMN
LHB
LIPA
LIPC
LIPE
LIPF
LIPG
LIPH
LIPI

LPAR1
LPAR2
LPAR3
LPAR5
LPCAT1
LPCAT2
LPCAT3
LPCAT4
LPGAT1
LPIN1
LPIN2
LPIN3
LPL
LRP2
LSS
LTA4H
LTC4S
LYPLA1
LYPLA2
MAOA
MAPK10
MAPK8
MAPK9
MAPKAPK2
MBOAT1
MBOAT2
MBOAT7
MBTPS1
MBTPS2
MCAT
MCEE
MDH1
MDH2
ME1
MECR
MED1
MED10
MED11
MED12
MED13
MED13L
MED14
MED15
MED16

MED17
MED18
MED19
MED20
MED21
MED22
MED23
MED24
MED25
MED26
MED27
MED28
MED29
MED30
MED31
MED4
MED6
MED7
MED8
MED9
METAP1
MFSD2A
MGLL
MID1IP1
MIF
MIGA1
MIGA2
MLYCD
MMAA
MMP1
MMUT
MOGAT1
MOGAT2
MOGAT3
MORC2
MSMO1
MTF1
MTM1
MTMR1
MTMR10
MTMR12
MTMR14
MTMR2
MTMR3

MTMR4
MTMR6
MTMR7
MTMR8
MTMR9
MTOR
MVD
MVK
NAGA
NBN
NCAPH2
NCOA1
NCOA2
NCOA3
NCOA6
NCOR1
NCOR2
NDUFAB1
NEU1
NEU2
NEU3
NEU4
NFKB1
NFKBIA
NFKBIB
NFKBIE
NFYA
NFYB
NFYC
NPAS2
NPY
NR1D1
NR1H2
NR1H3
NR1H4
NRF1
NSDHL
NTHL1
NUDT19
OCRL
ODC1
OLAH
OLR1
ORMDL1

ORMDL2
ORMDL3
OSBP
OSBPL10
OSBPL1A
OSBPL2
OSBPL3
OSBPL5
OSBPL6
OSBPL8
OSBPL9
OSTC
OXCT1
OXCT2
PCBD1
PCCA
PCCB
PCK1
PCK2
PCTP
PCYT1A
PCYT1B
PCYT2
PDHA1
PDHB
PDPK1
PECR
PEMT
PEX11A
PGS1
PHOSPHO1
PHYH
PI4K2A
PI4K2B
PI4KA
PI4KB
PIAS4
PIK3C2A
PIK3C2B
PIK3C2G
PIK3C3
PIK3CA
PIK3CB
PIK3CD

PIK3CG
PIK3R1
PIK3R2
PIK3R3
PIK3R4
PIK3R5
PIK3R6
PIKFYVE
PIP4K2A
PIP4K2B
PIP4K2C
PIP4P1
PIP5K1A
PIP5K1B
PIP5K1C
PISD
PITPNB
PITPNM1
PITPNM2
PITPNM3
PLA1A
PLA2G10
PLA2G12A
PLA2G12B
PLA2G15
PLA2G1B
PLA2G2A
PLA2G2C
PLA2G2D
PLA2G2E
PLA2G2F
PLA2G3
PLA2G4A
PLA2G4B
PLA2G4C
PLA2G4D
PLA2G4E
PLA2G4F
PLA2G5
PLA2G6
PLA2R1
PLAAT1
PLAAT2
PLAAT3

PLAAT4
PLAAT5
PLB1
PLBD1
PLD1
PLD2
PLD3
PLD4
PLD6
PLEKHA1
PLEKHA2
PLEKHA3
PLEKHA4
PLEKHA5
PLEKHA6
PLEKHA8
PLIN1
PLIN2
PLIN3
PLPP1
PLPP2
PLPP3
PLPP6
PLPPR1
PLPPR2
PLPPR3
PLPPR4
PLPPR5
PLTP
PMVK
PNLIP
PNLIPRP1
PNLIPRP2
PNPLA2
PNPLA3
PNPLA4
PNPLA5
PNPLA6
PNPLA7
PNPLA8
POMC
PON1
PON2
PON3

PPARA
PPARD
PPARG
PPARGC1A
PPARGC1B
PPM1L
PPP1CA
PPP1CB
PPP1CC
PPT1
PPT2
PRDX6
PRKAA1
PRKAA2
PRKAB1
PRKAB2
PRKACA
PRKACB
PRKACG
PRKAG1
PRKAG2
PRKAG3
PRKCQ
PRKD1
PRKD2
PRKD3
PRXL2B
PSAP
PSME1
PTDSS1
PTDSS2
PTEN
PTGDS
PTGES
PTGES2
PTGES3
PTGIS
PTGR1
PTGR2
PTGS1
PTGS2
PTPMT1
PTPN11
PTPN13

PTPRG
PTS
RAB14
RAB4A
RAB5A
RAN
RAP1GDS1
RDH11
RDH16
REEP6
RELA
RETSAT
RGL1
RORA
RUFY1
RXRA
RXRB
RXRG
S100A10
S1PR1
S1PR2
S1PR3
S1PR4
S1PR5
SACM1L
SAMD8
SAR1B
SBF1
SBF2
SC5D
SCAP
SCD
SCD5
SCP2
SDHA
SDHC
SDHD
SEC23A
SEC24A
SEC24B
SEC24C
SEC24D
SELENOI
SERINC1

SGMS1
SGMS2
SGPL1
SGPP1
SGPP2
SIN3A
SIN3B
SLC10A1
SLC10A2
SLC22A5
SLC25A1
SLC25A17
SLC25A20
SLC27A1
SLC27A2
SLC27A3
SLC27A4
SLC27A5
SLC27A6
SLC2A1
SLC2A4
SLC33A1
SLC44A1
SLC44A2
SLC44A3
SLC44A4
SLC44A5
SLCO1A2
SLCO1B1
SLCO1B3
SMARCD3
SMPD1
SMPD2
SMPD3
SMPD4
SMS
SOAT1
SOAT2
SOCS3
SORBS1
SP1
SPHK1
SPHK2
SPTLC1

SPTLC2
SPTLC3
SPTSSA
SPTSSB
SQLE
SRD5A1
SRD5A2
SRD5A3
SREBF1
SREBF2
ST3GAL1
ST3GAL2
ST3GAL3
ST3GAL4
ST3GAL5
ST3GAL6
ST6GALNAC3
ST6GALNAC4
ST6GALNAC5
ST6GALNAC6
ST8SIA1
ST8SIA5
STAR
STARD10
STARD3
STARD3NL
STARD4
STARD5
STARD6
STARD7
STAT3
STK11
STS
SUCLA2
SUCLG1
SUCLG2
SULT1E1
SULT2A1
SULT2B1
SUMF1
SUMF2
SUMO2
SYNJ1
SYNJ2

TAZ
TBL1X
TBL1XR1
TBXAS1
TDO2
TECR
TECRL
TGS1
THEM4
THEM5
THRAP3
THRSP
TIAM2
TKFC
TM7SF2
TMEM86B
TNF
TNFAIP8
TNFAIP8L1
TNFAIP8L2
TNFAIP8L3
TNFRSF1A
TNFRSF1B
TNFRSF21
TP53INP2
TPTE
TPTE2
TRADD
TRAF2
TRIB3
TSPO
TSPOAP1
TXNRD1
UBC
UBE2I
UBE2L6
UCP1
UGCG
UGDH
UGT1A1
UGT1A10
UGT1A3
UGT1A4
UGT1A5

UGT1A6
UGT1A7
UGT1A8
UGT1A9
UGT2A1
UGT2A3
UGT2B10
UGT2B11
UGT2B15
UGT2B17
UGT2B28
UGT2B4
UGT2B7
UGT8
UROD
UROS
VAC14
VAPA
VAPB
VDR
VNN1
XIST
YWHAH
GLS
GLS2
GLUD1
GLUD2
GOT2
KYAT1
OAT
PYCR1
PYCR2
PYCR3
RIMKLA
RIMKLB
AAAS
ABCC8
AC004551.1
AC016586.1
ACTN3
ADCY5
ADCY8
ADPGK
ADRA2A

AGER
AIFM1
ALDH5A1
ALDOB
ALDOC
ANLN
ANO1
APPL1
APPL2
AQP3
ARPP19
ARRB1
ART3
ASIP
ASNS
ASPSCR1
ATF4
ATG14
B3GNT6
BAD
BAIAP3
BCL2
BECN1
BHLHA15
BPGM
BRAF
BRAT1
BRS3
BRSK2
C12orf66
C1QTNF1
C1QTNF12
C1QTNF2
C2CD2L
C2CD5
C3
CALR
CANX
CAPN10
CARTPT
CASR
CBL
CCPG1
CDK1

CDK16
CDT1
CFTR
CLGN
CLIP3
CLTCL1
CLTRN
CMA1
COX11
CPB2
CPEB4
CREBL2
CREM
CRH
CXCL2
CYBA
DCXR
DDIT4
DLAT
DNAJB11
DNAJC3
DNM1L
DPP4
DRD1
DUSP12
DYNLL1
DYRK2
EDN1
EDNRA
EFNA5
EIF2AK3
ELF3
ENDOG
ENO1
ENPP1
ENY2
EPHA5
EPM2AIP1
ERN1
ERO1B
ESRRB
FBP1
FBP2
FFAR1

FFAR4
FGF19
FGF21
FIS1
FKBP1B
FKBP2
FOXA2
FOXA3
FOXO1
FOXO3
G6PC3
G6PD
GAA
GABARAPL3
GADD45A
GALM
GAPDH
GAS6
GATA4
GCG
GCGR
GCK
GCKR
GCLC
GCLM
GDPGP1
GFAP
GFPT1
GH1
GHRHR
GHRL
GIP
GJB6
GNB3
GNPDA1
GNPDA2
GOT1
GPC3
GPER1
GPI
GPLD1
GPR119
GPR27
GPR68

GRB10
GRIK5
GSDME
GSK3A
GSK3B
GYS1
H6PD
HECTD4
HIF1A
HIGD1A
HIST1H4J
HK1
HK2
HK3
HKDC1
HMGN3
HNF1A
HNRNPA1
HSPA5
ICAM1
IFI16
IGF1
IGF1R
IGF2
IGFBP3
IGFBP4
IGFBP5
IL1B
IMPACT
INS
INSR
ISL1
ITFG2
ITLN1
JAGN1
KCNB1
KCNF1
KCNJ11
KDELR2
KHK
KIF5B
KIF5C
KLF15
KPTN

LCMT1
LIN28A
LNPEP
LRP1
LRP5
LRP5L
MAP4K4
MAPK14
MEF2A
MEN1
MIDN
MIR103A1
MIR103A2
MIR107
MIR143
MIR15A
MIR16-1
MIR16-2
MIR223
MIRLET7G
MLXIPL
MPC2
MYBBP1A
NADK
NDC1
NDUFAF2
NDUFB3
NEUROD1
NFE2L1
NFE2L2
NGFR
NIM1K
NISCH
NKX1-1
NME1
NME2
NOX4
NPY1R
NR4A3
NUAK2
NUCKS1
NUP107
NUP133
NUP153

NUP155
NUP160
NUP188
NUP205
NUP210
NUP214
NUP35
NUP37
NUP43
NUP50
NUP54
NUP58
NUP62
NUP85
NUP88
NUP93
NUP98
NUPL2
OCLN
OMA1
ONECUT1
OPA1
OPRK1
OSTN
PASK
PAX2
PAX6
PBK
PC
PCSK1
PDHA2
PDIA3
PDIA4
PDIA6
PDK1
PDK2
PDK3
PDK4
PDX1
PEA15
PFKFB1
PFKFB2
PFKFB3
PFKFB4

PFKL
PFKM
PFKP
PGAM1
PGAM2
PGK1
PGK2
PGM1
PGM2
PGM2L1
PGM5
PGP
PHKG2
PHLDA2
PHPT1
PICK1
PID1
PIGA
PIH1D1
PIM3
PKLR
PKM
PLK3
PMAIP1
POLG
POM121
POM121C
POU4F2
PPEF2
PPIB
PPP1R3A
PPP1R3B
PPP1R3D
PPP1R3E
PPP1R3F
PPP1R3G
PPP2CA
PPP2CB
PPP2R1A
PPP2R1B
PPP2R5D
PPP3CA
PPP3CB
PRAME

PREB
PRKCB
PRKCE
PRKCI
PRKCZ
PRKN
PTH
PTPRN
PTPRN2
PYGL
RAB11B
RAB11FIP2
RAB11FIP5
RAB4B
RACK1
RAE1
RAF1
RANBP2
RAP1A
RELB
RFX6
RGN
RHOQ
RNASEL
RORC
RPS6KB1
RRM2
RRP8
RSC1A1
RTN2
SARS
SEC11A
SEC11C
SEC13
SEH1L
SELENOS
SELENOT
SEMG1
SEPHS2
SERP1
SERPINF1
SESN1
SESN2
SESN3

SFN
SGK3
SH3GLB1
SIDT2
SIK1
SIK1B
SIK2
SIRT1
SLC1A2
SLC25A10
SLC25A11
SLC25A12
SLC25A13
SLC25A27
SLC29A1
SLC2A3
SLC2A8
SLC35B1
SLC37A1
SLC37A2
SLC37A4
SLC3A2
SLC45A3
SLC5A1
SLC5A10
SLC5A11
SLC5A2
SLC5A3
SLC5A4
SLC5A9
SLC9B2
SMARCA4
SMARCB1
SORD
SORT1
SOX4
SPCS1
SPCS2
SPCS3
SRC
SRF
SRI
STX4
STXBP3

STXBP4
SYBU
SZT2
TBC1D4
TBL2
TERT
TFAP2B
TFF3
TFRC
TGIF1
TH
TIAM1
TIGAR
TKTL1
TP53
TPI1
TPR
TRA2B
TRARG1
TRIP10
TRPM4
TRPS1
TSC1
TWNK
TXNIP
UCP2
UGP2
UNC13B
UPP1
USF1
USF2
VAMP2
VSNL1
WDR35
WDTC1
XBP1
YES1
YWHAB
YWHAE
YWHAG
YWHAQ
YWHAZ
ZAP70
ZBTB20

ZC3H12A
ZMPSTE24
ZNF236
ACSS2
ALDH1A3
G6PC1
LDHAL6A
LDHAL6B
LDHB
LDHC
PGAM4
ACO1
CS
DHTKD1
IDH2
IDH3A
MDH1B
MRPS36
NNT
OGDH
OGDHL
SDHAF2
SDHB
ABCC6
AK1
AK2
AK4
AK5
AK9
ANTKMT
APP
ARL2
ATP1A2
ATP1B1
ATP5F1A
ATP5F1B
ATP5F1C
ATP5F1D
ATP5F1E
ATP5F1EP2
ATP5IF1
ATP5MC1
ATP5MC2
ATP5MC3

ATP5ME
ATP5MF
ATP5MG
ATP5MGL
ATP5MJ
ATP5MK
ATP5PB
ATP5PD
ATP5PF
ATP5PO
ATP6V0C
ATP6V1A
ATP6V1B1
ATP6V1B2
ATPSCKMT
BEND3
CBFA2T3
CLPX
CTNS
DGUOK
DMAC2L
DNAJC30
EIF6
ENO4
ENPP3
FIGNL1
FLCN
FOXK1
FOXK2
GALK1
GIT1
H19
HDAC4
HSPA1A
HSPA1B
HSPA8
HTR2A
IFNG
IL4
JMJD8
MIR675
MLST8
MT-ATP6
MT-ATP8

MT-CO2
MT-ND1
MT-ND2
MT-ND3
MT-ND4
MT-ND4L
MT-ND5
MT-ND6
MTCO2P12
MYH3
MYH4
MYH6
MYH7
MYH8
NDUFA1
NDUFA10
NDUFA11
NDUFA12
NDUFA13
NDUFA2
NDUFA3
NDUFA5
NDUFA6
NDUFA7
NDUFA8
NDUFA9
NDUFB1
NDUFB10
NDUFB11
NDUFB2
NDUFB4
NDUFB5
NDUFB6
NDUFB7
NDUFB8
NDUFB9
NDUFC1
NDUFC2
NDUFS1
NDUFS2
NDUFS3
NDUFS4
NDUFS5
NDUFS6

NDUFS7
NDUFS8
NDUFV1
NDUFV2
NDUFV3
NMNAT1
NT5E
NUDT2
NUDT5
NUPR1
OGT
OLA1
P2RX7
PARG
PARP1
PINK1
PRXL2C
PSEN1
RPTOR
SIRT6
SLC25A25
SLC2A6
SLC4A1
SLC4A4
STOML2
TAFAZZIN
TGFB1
TMSB4X
TREM2
TREX1
UQCC3
VCP
VPS9D1
ZBTB7A
AANAT
AASS
ACAD8
ACMSD
ADI1
ADO
AFMID
AGMAT
AGXT
AGXT2

AHCY
AIMP1
AIMP2
ALDH18A1
ALDH4A1
ALDH6A1
AMD1
AMDHD1
AMT
APIP
ARG1
ARG2
ASL
ASMT
ASPA
ASPG
ASRGL1
ASS1
AZIN1
AZIN2
BBOX1
BCAT1
BCAT2
BCKDHA
BCKDK
BHMT
BHMT2
CARNMT1
CARNS1
CBS
CDO1
CHDH
CKB
CKM
CKMT1A
CKMT1B
CKMT2
CPS1
CRYM
CSAD
CTH
DAO
DARS1
DBH

DBT
DCT
DDC
DDO
DIO1
DIO2
DIO3
DMGDH
DUOX1
DUOX2
EEF1E1
EEFSEC
ENOPH1
EPRS1
ETHE1
FAH
FAU
FOLH1
FTCD
GADL1
GAMT
GATM
GCAT
GCSH
GLDC
GNMT
GPT
GPT2
GSR
HAAO
HAL
HAO1
HDC
HGD
HIBADH
HNMT
HOGA1
HPD
HYKK
IARS1
IDO1
IDO2
IVD
IYD

KARS1
KMO
KYAT3
KYNU
LARS1
LIAS
LIPT1
LIPT2
MARS1
MAT1A
MCCC1
MCCC2
MPST
MRI1
MTAP
MTR
MTRR
NAALAD2
NAGS
NAT8L
NMRAL1
NNMT
NQO1
OAZ1
OAZ2
OAZ3
OCA2
OTC
PAH
PAOX
PAPSS1
PAPSS2
PDHX
PHGDH
PHYKPL
PIPOX
PNMT
PPM1K
PRODH
PRODH2
PSAT1
PSMA1
PSMA2
PSMA3

PSMA4
PSMA5
PSMA6
PSMA7
PSMA8
PSMB1
PSMB10
PSMB11
PSMB2
PSMB3
PSMB4
PSMB5
PSMB6
PSMB7
PSMB8
PSMB9
PSMC1
PSMC2
PSMC3
PSMC4
PSMC5
PSMC6
PSMD1
PSMD10
PSMD11
PSMD12
PSMD13
PSMD14
PSMD2
PSMD3
PSMD4
PSMD5
PSMD6
PSMD7
PSMD8
PSMD9
PSME2
PSME3
PSME4
PSMF1
PSPH
PSTK
PXMP2
QARS1

QDPR
RARS1
RIDA
RPL10
RPL10A
RPL10L
RPL11
RPL12
RPL13
RPL13A
RPL14
RPL15
RPL17
RPL18
RPL18A
RPL19
RPL21
RPL22
RPL22L1
RPL23
RPL23A
RPL24
RPL26
RPL26L1
RPL27
RPL27A
RPL28
RPL29
RPL3
RPL30
RPL31
RPL32
RPL34
RPL35
RPL35A
RPL36
RPL36A
RPL36AL
RPL37
RPL37A
RPL38
RPL39
RPL39L
RPL3L

RPL4
RPL41
RPL5
RPL6
RPL7
RPL7A
RPL8
RPL9
RPLP0
RPLP1
RPLP2
RPS10
RPS11
RPS12
RPS13
RPS14
RPS15
RPS15A
RPS16
RPS17
RPS18
RPS19
RPS2
RPS20
RPS21
RPS23
RPS24
RPS25
RPS26
RPS27
RPS27A
RPS27L
RPS28
RPS29
RPS3
RPS3A
RPS4X
RPS4Y1
RPS4Y2
RPS5
RPS6
RPS7
RPS8
RPS9

RPSA
SARDH
SARS1
SAT1
SCLY
SDS
SDSL
SECISBP2
SEM1
SEPSECS
SERINC2
SERINC3
SERINC4
SERINC5
SHMT1
SLC25A15
SLC25A2
SLC25A21
SLC25A44
SLC36A4
SLC45A2
SLC5A5
SLC6A11
SLC6A12
SLC6A7
SLC6A8
SLC7A5
SMOX
SQOR
SRM
SRR
SUOX
TAT
TMLHE
TPH1
TPH2
TPO
TSHB
TST
TSTD1
TXN2
TYR
TYRP1
UBA52

UROC1
