# Supplementary material for: Identification and Validation of a Metabolism-Related Prognostic Signature Associated with M2 Macrophage Infiltration in Gastric Cancer
Source: Int J Mol Sci. 2023 Jun 25;24(13):10625. doi: 10.3390/ijms241310625 (PMC10342140; doi:10.3390/ijms241310625)
Supplement: Supplementary file 1 [file ijms-24-10625-s001.zip › Supplementary file S2.pdf]

**Supplementary file 2**

**Primers for qRT-PCR**

| **Transcript** | **Forward** | **Reverse** |
|---|---|---|
| SRI | ATCAAGAGGAAGCTGCATGA | ATTACCGAAGGCAAAGAGGA |
| TNF-α | CAGACTTC CTTGAGACACGG | CAAGGCAGCTCCTACATTGG |
| CD86 | CTGCTCATCTATACACGGTTACC | GGAAACGTCGTACAGTTCTGTG |
| CD163 | GCGGGAGAGTGGAAGTGAAAG | GTTACAAATCACAGAGACCGCT |
| CD206 | CTACAAGGGATCGGGTTTATGGA | TTGGCATTGCCTAGTAGCGTA |
| GAPDH | GACAGTCAGCCGCATCTTCT | TTAAAAGCAGCCCTGGTGAG |
