# Supplementary material for: Identification and Validation of a Metabolism-Related Prognostic Signature Associated with M2 Macrophage Infiltration in Gastric Cancer
Source: Int J Mol Sci. 2023 Jun 25;24(13):10625. doi: 10.3390/ijms241310625 (PMC10342140; doi:10.3390/ijms241310625)
Supplement: Supplementary file 1 [file ijms-24-10625-s001.zip › Supplementary file S3.pdf]

Supplementary file 3

Cell	Gene	Cor(TCGA)	pvalue
Macrophages M2	TREM2	0.531001	1.05E-14
Macrophages M2	OLR1	0.430199	1.22E-09
Macrophages M2	ASAH1	0.425259	1.96E-09
Macrophages M2	LIPA	0.416665	4.44E-09
Macrophages M2	NOX4	0.39662	2.72E-08
Macrophages M2	STARD3NL	0.392071	4.05E-08
Macrophages M2	LGMN	0.390561	4.61E-08
Macrophages M2	SERPINF1	0.382444	9.16E-08
Macrophages M2	PEA15	0.377772	1.35E-07
Macrophages M2	HSD17B14	0.36922	2.70E-07
Macrophages M2	CPT1C	0.357939	6.53E-07
Macrophages M2	HSD11B1	0.355005	8.18E-07
Macrophages M2	LGALS1	0.34859	1.33E-06
Macrophages M2	PLD3	0.34772	1.41E-06
Macrophages M2	ARSB	0.347638	1.42E-06
Macrophages M2	ABCA1	0.344451	1.80E-06
Macrophages M2	PPT1	0.342478	2.08E-06
Macrophages M2	CYP1B1	0.32905	5.41E-06
Macrophages M2	RGL1	0.328938	5.46E-06
Macrophages M2	FMO1	0.327853	5.88E-06
Macrophages M2	LPL	0.326919	6.27E-06

Macrophages M2	BCAT1	0.325226	7.05E-06
Macrophages M2	ZC3H12A	-0.32374	7.80E-06
Macrophages M2	DYNLL1	0.321408	9.15E-06
Macrophages M2	ARSK	0.320528	9.71E-06
Macrophages M2	NDUFS4	0.320458	9.75E-06
Macrophages M2	P2RX7	0.317789	1.17E-05
Macrophages M2	ALDH3A1	-0.31681	1.25E-05
Macrophages M2	ATP6V1B2	0.315466	1.36E-05
Macrophages M2	RAB4A	0.315025	1.40E-05
Macrophages M2	HEXB	0.311816	1.73E-05
Macrophages M2	PTGIS	0.308593	2.14E-05
Macrophages M2	HEXA	0.30788	2.24E-05
Macrophages M2	EDNRA	0.306703	2.41E-05
Macrophages M2	BCHE	0.304666	2.75E-05
Macrophages M2	OSTC	0.303385	2.98E-05
Macrophages M2	SLC5A5	-0.30088	3.49E-05
Macrophages M2	ADO	0.299017	3.93E-05
Macrophages M2	SDS	0.294922	5.06E-05
Macrophages M2	GNPDA2	0.288514	7.47E-05
Macrophages M2	PRKD1	0.286449	8.45E-05
Macrophages M2	NDUFA8	0.282293	0.000108
Macrophages M2	DUOX2	-0.28114	0.000116

Macrophages M2	GABARAPL1	0.279862	0.000125
Macrophages M2	H19	0.279811	0.000125
Macrophages M2	ADH5	0.272575	0.000189
Macrophages M2	GLIPR1	0.266191	0.00027
Macrophages M2	GCKR	-0.26534	0.000283
Macrophages M2	TST	-0.26524	0.000285
Macrophages M2	APP	0.264964	0.000289
Macrophages M2	PPP3CB	0.263648	0.000311
Macrophages M2	SH3GLB1	0.262322	0.000334
Macrophages M2	IGF1	0.26047	0.000369
Macrophages M2	HNMT	0.258647	0.000407
Macrophages M2	IGFBP4	0.25644	0.000458
Macrophages M2	HACD1	0.255572	0.00048
Macrophages M2	BPGM	0.255485	0.000482
Macrophages M2	GPX7	0.254975	0.000495
Macrophages M2	GGT5	0.254672	0.000503
Macrophages M2	CYP2U1	0.254308	0.000513
Macrophages M2	NUPR1	0.253352	0.000539
Macrophages M2	GDPD3	-0.25272	0.000557
Macrophages M2	PRKAG1	0.252694	0.000558
Macrophages M2	APOD	0.251189	0.000604
Macrophages M2	PYGL	0.249031	0.000675

Macrophages M2	SLC25A10	-0.24841	0.000697
Macrophages M2	GPX1	0.248366	0.000699
Macrophages M2	PITPNB	0.244007	0.000872
Macrophages M2	SRR	0.242349	0.000948
Macrophages M2	MDH1	0.241543	0.000988
Macrophages M2	IGFBP3	0.240737	0.001028
Macrophages M2	GNPDA1	0.240322	0.00105
Macrophages M2	GHRL	-0.24024	0.001054
Macrophages M2	GPX2	-0.23889	0.001127
Macrophages M2	CA2	-0.23819	0.001167
Macrophages M2	RAB4B	-0.23738	0.001214
Macrophages M2	LRP1	0.236421	0.001272
Macrophages M2	GGT1	-0.2345	0.001397
Macrophages M2	FA2H	-0.23327	0.001483
Macrophages M2	CXCL2	-0.23279	0.001518
Macrophages M2	ELOVL4	0.23255	0.001536
Macrophages M2	CYP4F12	-0.23199	0.001577
Macrophages M2	SEC23A	0.23186	0.001587
Macrophages M2	NDUFB2	0.231852	0.001588
Macrophages M2	INMT	0.231024	0.001652
Macrophages M2	CKMT1B	-0.22993	0.001741
Macrophages M2	CDO1	0.227811	0.001925

Macrophages M2	ELOVL5	0.227375	0.001965
Macrophages M2	NDUFB5	0.226695	0.002029
Macrophages M2	NDUFAF2	0.225738	0.002122
Macrophages M2	GM2A	0.225497	0.002146
Macrophages M2	CDK8	0.224927	0.002204
Macrophages M2	PI4K2A	0.224345	0.002265
Macrophages M2	SRI	0.224277	0.002272
Macrophages M2	CBR3	0.22376	0.002327
Macrophages M2	PSMD10	0.223505	0.002355
Macrophages M2	ALDH1A3	0.222963	0.002415
Macrophages M2	PCCA	0.222817	0.002431
Macrophages M2	GPT	-0.22266	0.002449
Macrophages M2	CYP27A1	0.221815	0.002546
Macrophages M2	CDIPT	0.221318	0.002605
Macrophages M2	CLIP3	0.220371	0.002721
Macrophages M2	ALDH1B1	0.219926	0.002777
Macrophages M2	CYP2C9	-0.21859	0.002951
Macrophages M2	PPP2CB	0.217299	0.003129
Macrophages M2	LIPF	-0.21559	0.003379
Macrophages M2	PIM3	-0.21337	0.003731
Macrophages M2	ATP1A2	0.211541	0.004045
Macrophages M2	CYP3A5	-0.21114	0.004117

Macrophages M2	CKMT1A	-0.21024	0.004282
Macrophages M2	RPL27	0.204106	0.005582
Macrophages M2	PRKAB2	0.201946	0.006117
Macrophages M2	PGM5	0.201784	0.006159
Macrophages M2	SFN	-0.20155	0.006219
Macrophages M2	C1QTNF1	0.200281	0.00656
Macrophages M2	DGKD	-0.19974	0.006711
Macrophages M2	RPS27L	0.199324	0.006828
Macrophages M2	DHCR24	-0.19893	0.00694
Macrophages M2	NUCKS1	0.198583	0.007042
Macrophages M2	ESRRA	-0.19741	0.007393
Macrophages M2	CYP2C18	-0.19629	0.007741
Macrophages M2	ADH7	-0.19272	0.008956
Macrophages M2	GLB1L	0.191583	0.009376
Macrophages M2	PINK1	0.190232	0.009898
Macrophages M2	CAV1	0.189239	0.010298
Macrophages M2	SCD5	0.188752	0.010499
Macrophages M2	RPS23	0.186882	0.011305
Macrophages M2	FUT9	-0.18295	0.013179
Macrophages M2	SLC44A4	-0.18241	0.013454
Macrophages M2	SLC45A3	-0.17995	0.014789
Macrophages M2	ALDH2	-0.17774	0.016081

Macrophages M2	FUT2	-0.17769	0.016106
Macrophages M2	PFKM	0.177673	0.016119
Macrophages M2	CA4	-0.17724	0.016386
Macrophages M2	RORC	-0.17652	0.016831
Macrophages M2	MVD	-0.17464	0.018055
Macrophages M2	COMT	0.172225	0.019737
Macrophages M2	PHLDA2	-0.17213	0.019809
Macrophages M2	TMEM86B	-0.17208	0.019843
Macrophages M2	ILK	0.171164	0.020519
Macrophages M2	RPL35A	0.170864	0.020744
Macrophages M2	D2HGDH	-0.17078	0.020806
Macrophages M2	TBXAS1	0.169765	0.02159
Macrophages M2	ABCC3	-0.16921	0.022027
Macrophages M2	STARD10	-0.16756	0.02338
Macrophages M2	RPS24	0.167442	0.023476
Macrophages M2	ADH1B	0.165496	0.025162
Macrophages M2	PASK	-0.16532	0.025321
Macrophages M2	ELOVL6	-0.16461	0.025962
Macrophages M2	SMPD3	-0.164	0.026529
Macrophages M2	DIO2	0.163456	0.027041
Macrophages M2	FIS1	0.163264	0.027224
Macrophages M2	GLYCTK	-0.16319	0.027298

Macrophages M2	FAAH	-0.16317	0.027313
Macrophages M2	NQO1	-0.16199	0.028467
Macrophages M2	ASL	-0.16165	0.028806
Macrophages M2	ZAP70	-0.16163	0.028827
Macrophages M2	NDUFB11	0.160422	0.030057
Macrophages M2	GPT2	-0.1597	0.030812
Macrophages M2	LEPR	0.158925	0.031647
Macrophages M2	GLDC	-0.15736	0.033389
Macrophages M2	FOXA3	-0.15148	0.040659
Macrophages M2	MPST	-0.15077	0.041621
Macrophages M2	ALDH1A1	-0.15042	0.042106
Macrophages M2	PIK3C2G	-0.15024	0.042351
Macrophages M2	A4GALT	0.148898	0.044254
Macrophages M2	GPD1	-0.14877	0.044439
Macrophages M2	MAPK10	0.148149	0.045345
Macrophages M2	PNLIPRP2	-0.14773	0.045961
Macrophages M2	KCNB1	0.146997	0.047065
Macrophages M2	CYP2C8	-0.14671	0.047503
Macrophages M2	NDUFB6	0.145467	0.049434
Macrophages M2	PICK1	-0.14537	0.049583
