# Supplementary material for: Identification and Validation of a Metabolism-Related Prognostic Signature Associated with M2 Macrophage Infiltration in Gastric Cancer
Source: Int J Mol Sci. 2023 Jun 25;24(13):10625. doi: 10.3390/ijms241310625 (PMC10342140; doi:10.3390/ijms241310625)
Supplement: Supplementary file 1 [file ijms-24-10625-s001.zip › Supplementary file S4.pdf]

Supplementary file 4

Gene	Coef
NOX4	0.100844621411362
STARD3NL	0.0237286722230089
ABCA1	0.0457897125263643
LPL	0.0493429851736185
DYNLL1	0.242087266953199
SLC5A5	0.119571124384521
APOD	0.0535727331078263
ELOVL4	0.00218250803600714
SRI	0.242905051486043
FUT2	-0.0646240860226172
SMPD3	-0.0182249057638638
FAAH	-0.197949344375956
