# Supplementary material for: Identification and Validation of a Metabolism-Related Prognostic Signature Associated with M2 Macrophage Infiltration in Gastric Cancer
Source: Int J Mol Sci. 2023 Jun 25;24(13):10625. doi: 10.3390/ijms241310625 (PMC10342140; doi:10.3390/ijms241310625)
Supplement: Supplementary file 1 [file ijms-24-10625-s001.zip › Supplementary file S5 .pdf]

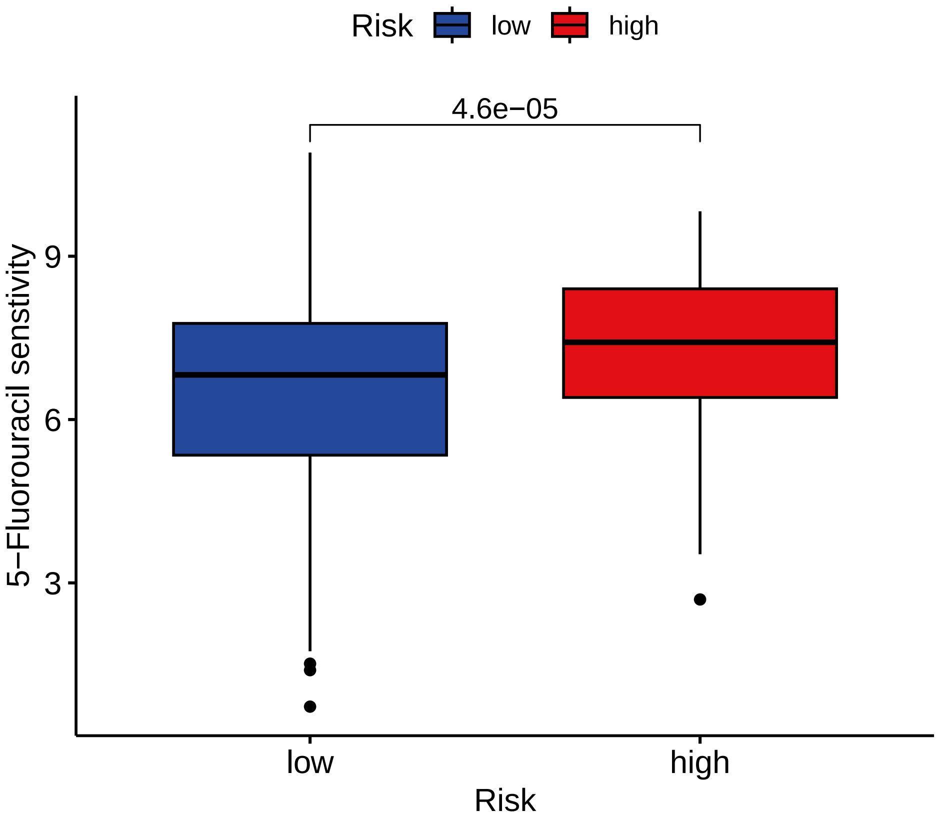
Risk
low
high
4.6e−05
5−Fluorouracil senstivity
9
6
3
low
high
Risk

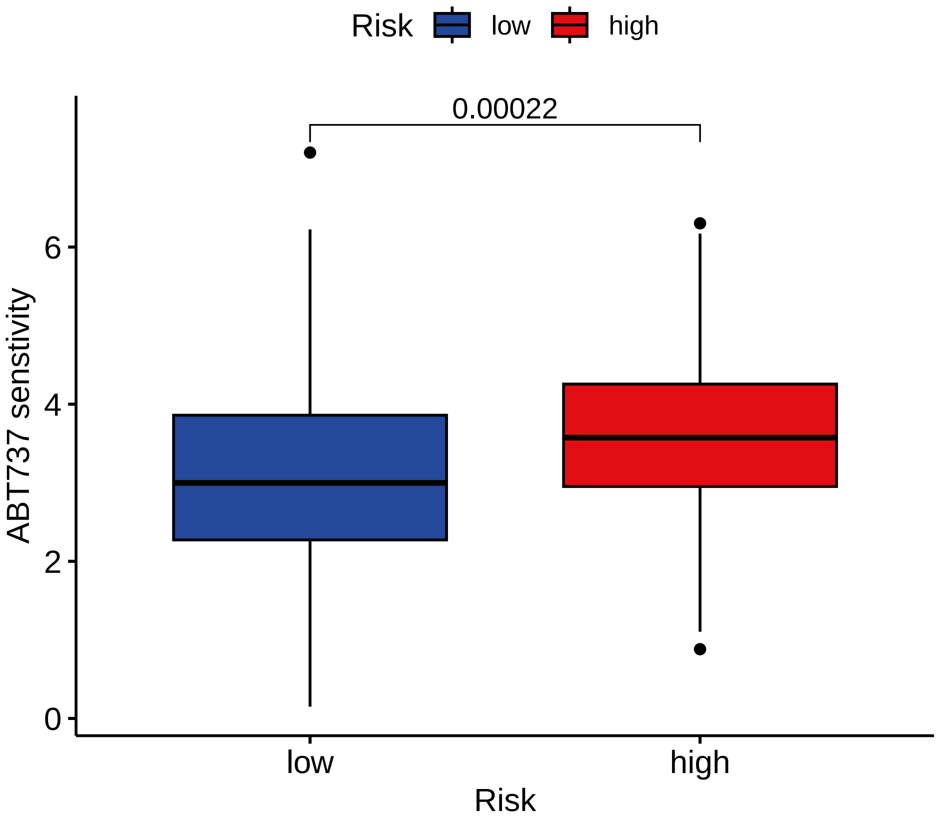

Risk
low
high
0.00022
ABT737 senstivity
6
4
2
0
low
high
Risk

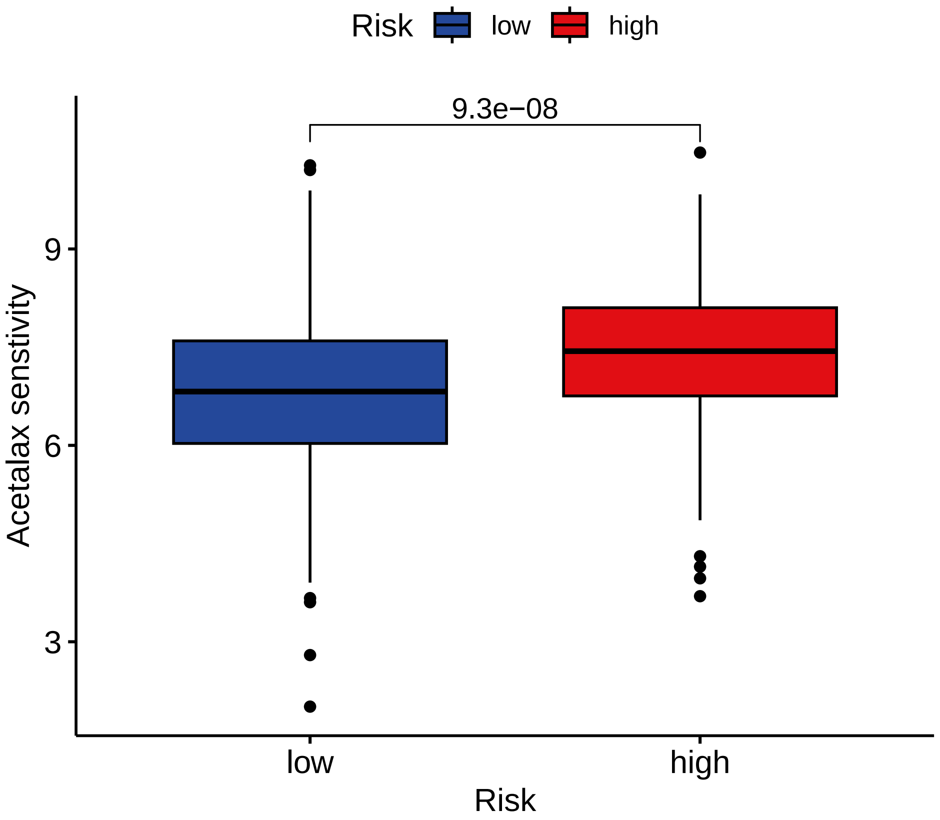

Risk
low
high
9.3e−08
Acetalax senstivity
9
6
3
low
high
Risk

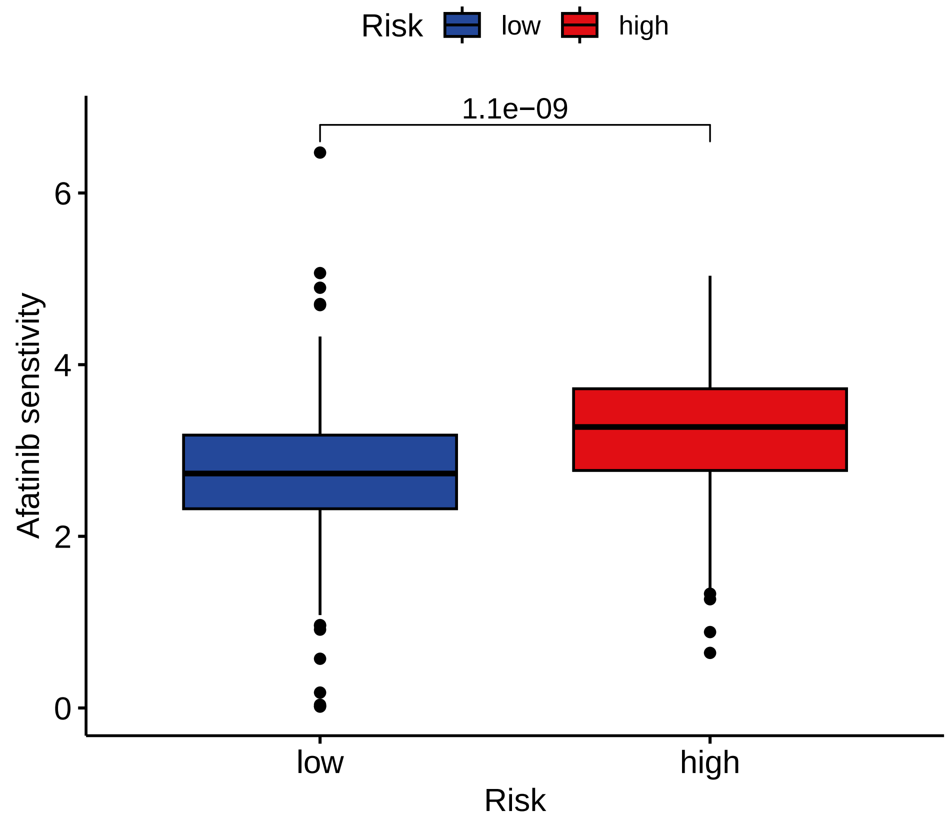
Risk
low
high
1.1e−09
Afatinib senstivity
0
2
4
6
low
high
Risk

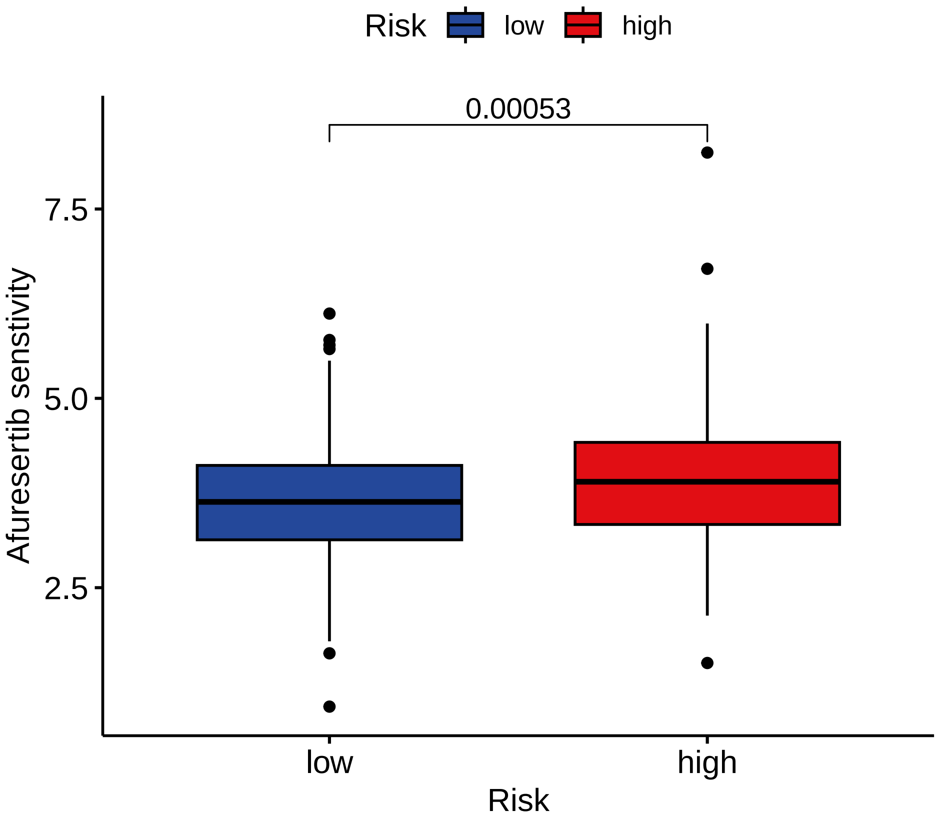
Risk
low
high
0.00053
7.5
5.0
2.5
Afuresertib senstivity
low
high
Risk

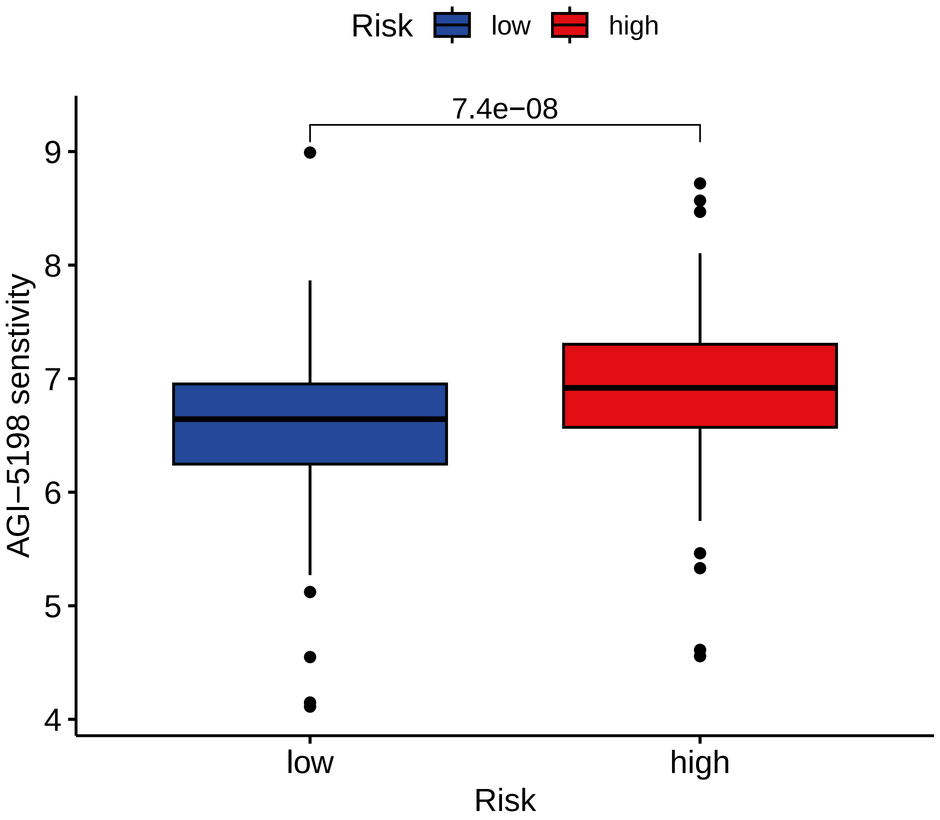

Risk
low
high
7.4e−08
9
8
7
6
5
4
AGI−5198 senstivity
low
high
Risk

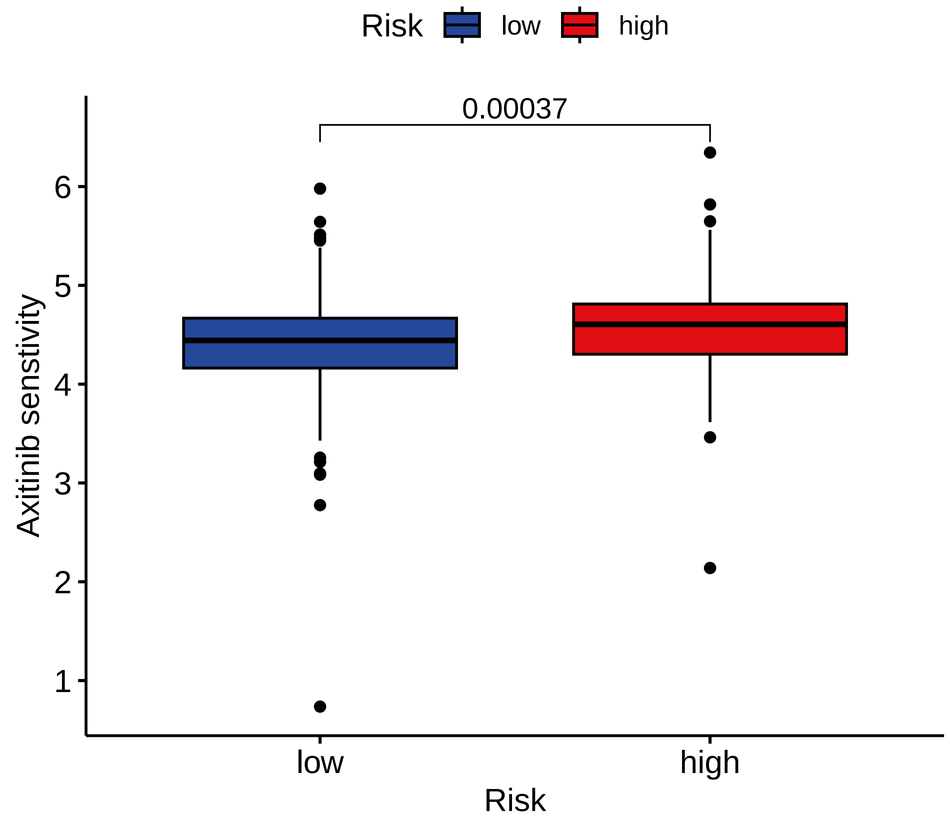

Risk
low
high
0.00037
Axitinib senstivity
6
5
4
3
2
1
low
high
Risk

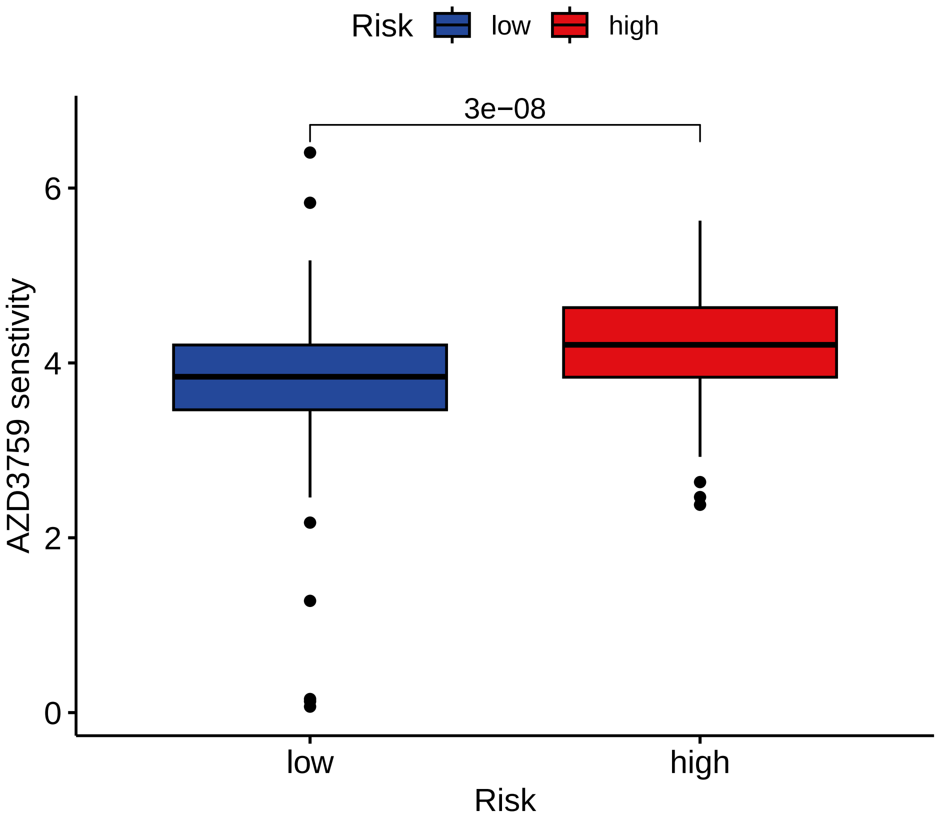
Risk
low
high
3e−08
AZD3759 senstivity
6
4
2
0
low
high
Risk

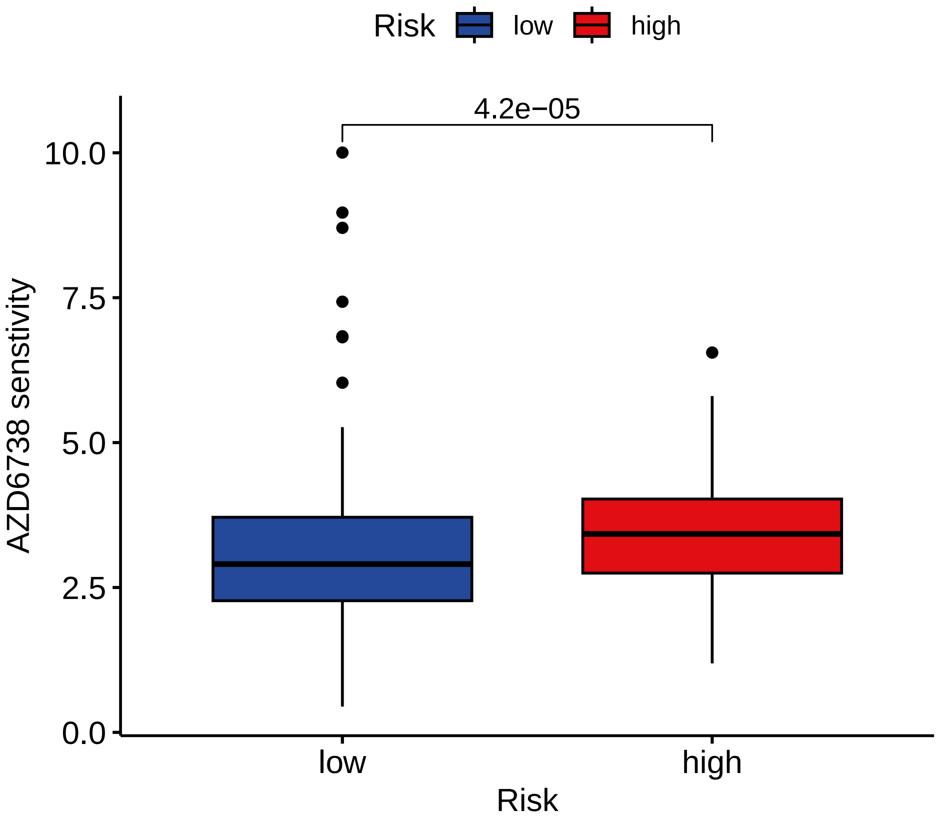

Risk
low
high
4.2e−05
AZD6738 senstivity
10.0
7.5
5.0
2.5
0.0
low
high
Risk

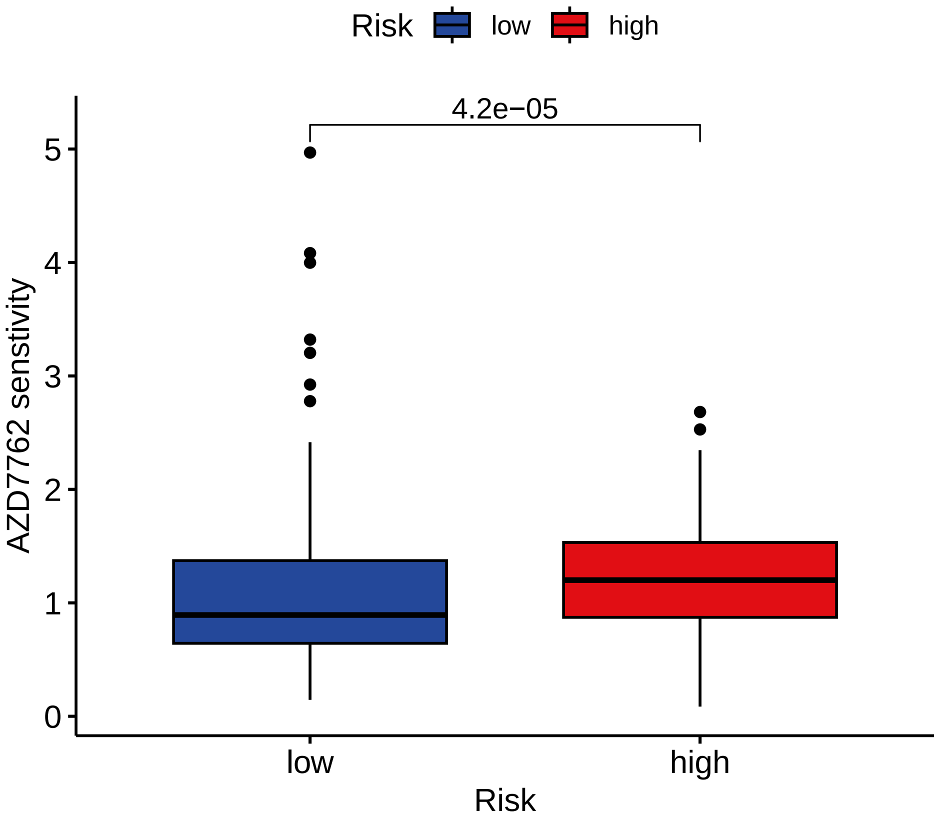

Risk
low
high
4.2e−05
AZD7762 senstivity
5
4
3
2
1
0
low
high
Risk

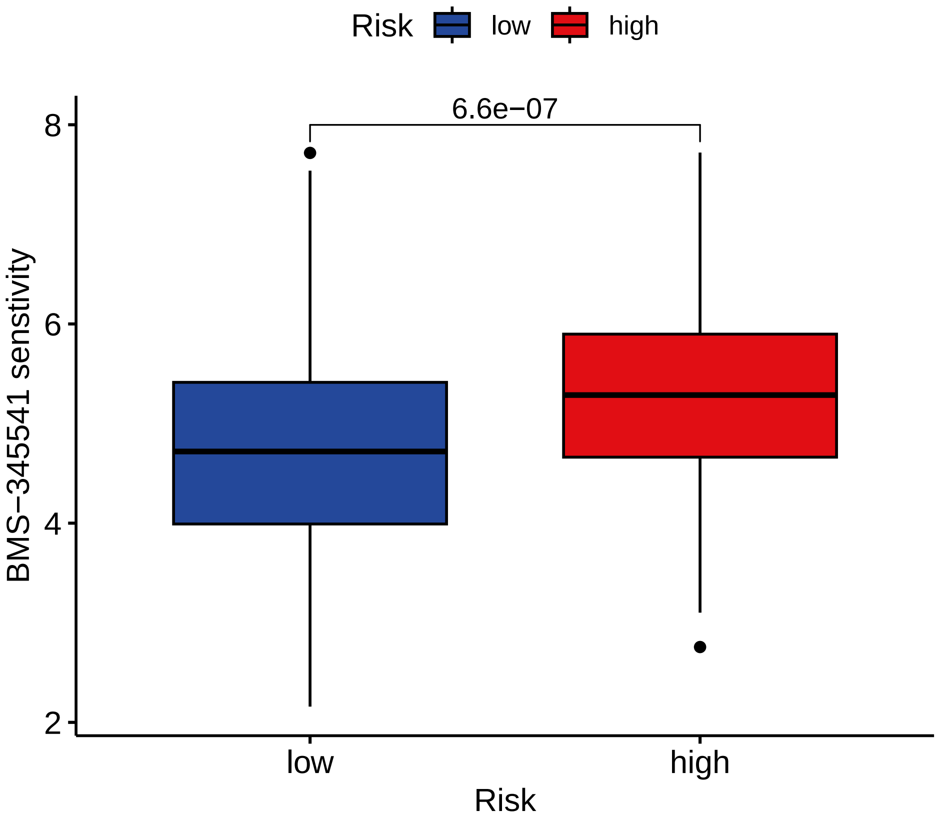
Risk
low
high
6.6e−07
8
6
4
2
BMS−345541 senstivity
low
high
Risk

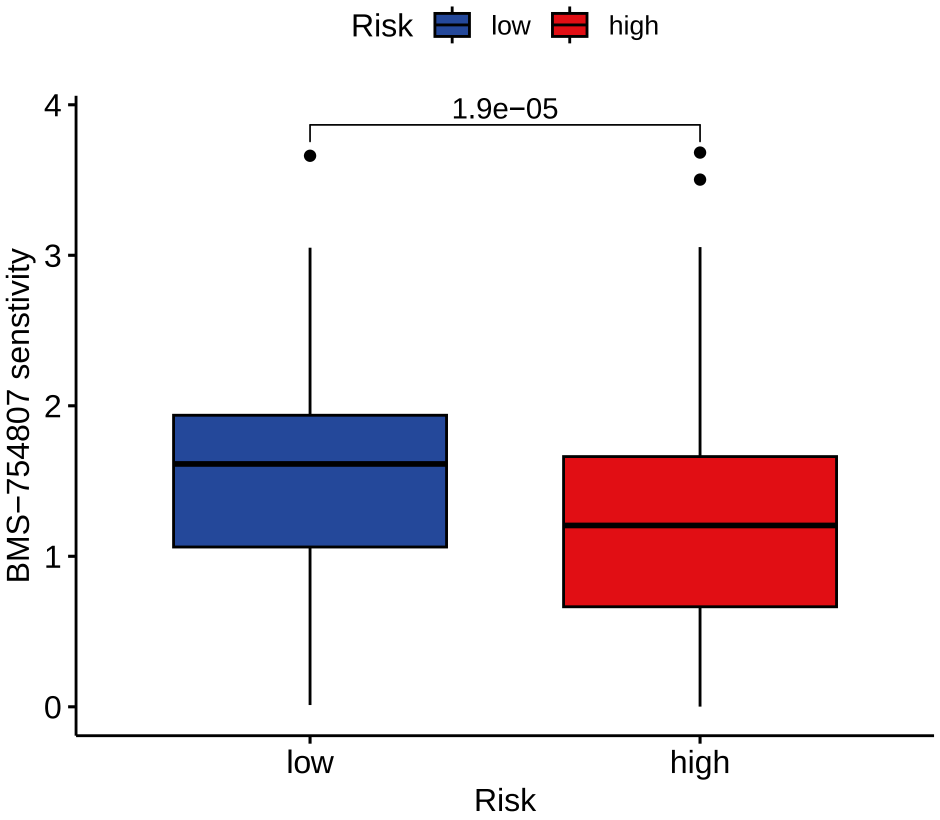
Risk
low
high
1.9e−05
BMS−754807 senstivity
0
1
2
3
4
low
high
Risk

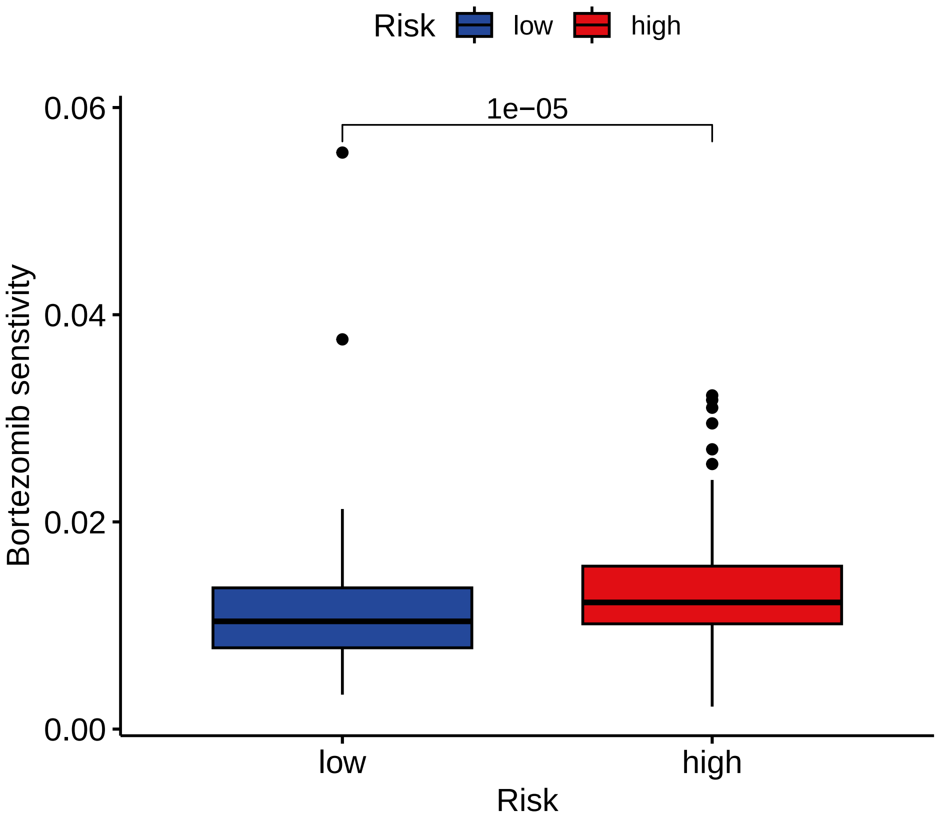

Risk
low
high
1e−05
0.06
0.04
0.02
0.00
Bortezomib senstivity
low
high
Risk

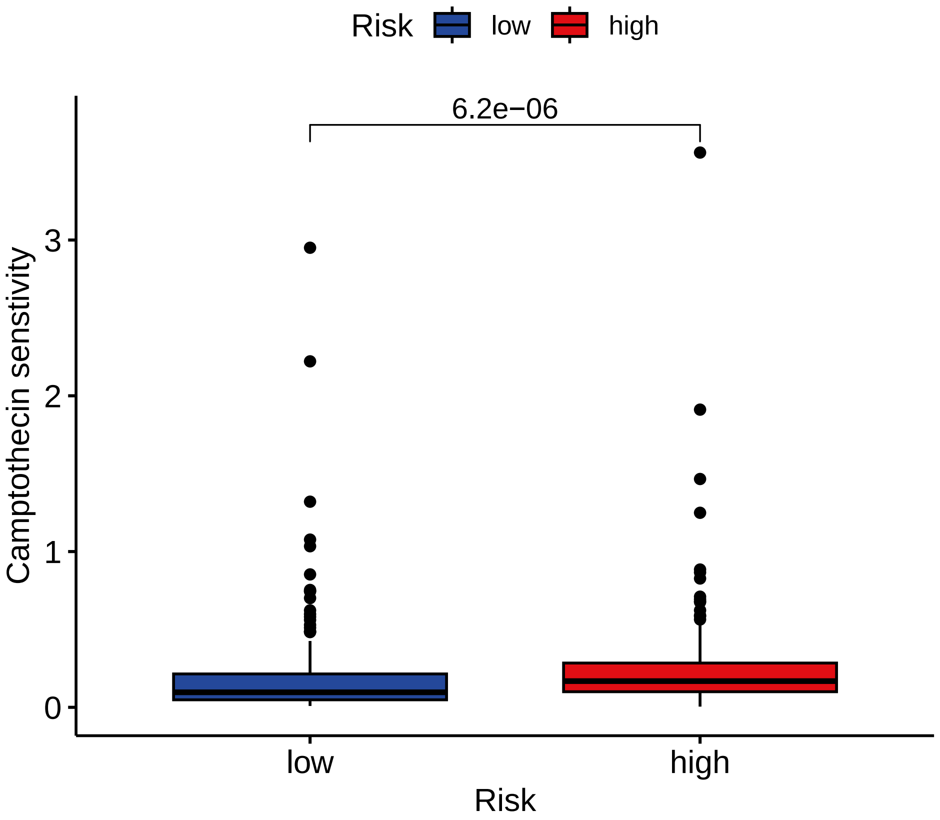
Risk
low
high
6.2e−06
Camptothecin senstivity
0
1
2
3
low
high
Risk

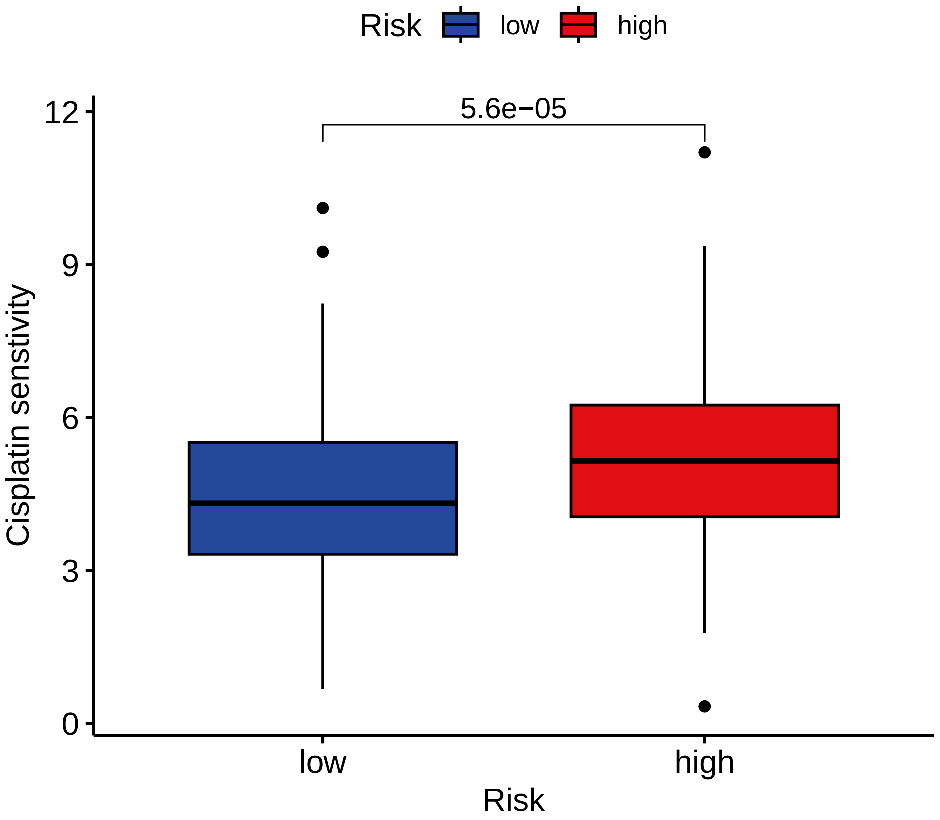
Risk
low
high
5.6e−05
12
9
6
3
0
Cisplatin senstivity
low
high
Risk

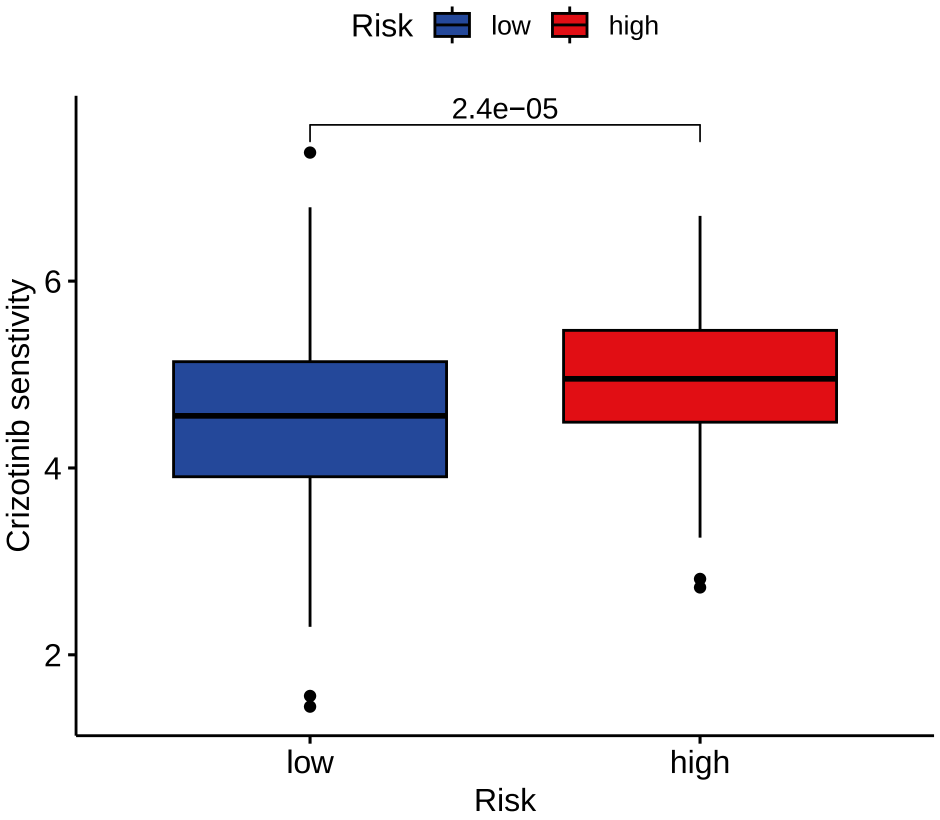
Risk
low
high
2.4e−05
Crizotinib senstivity
6
4
2
low
high
Risk

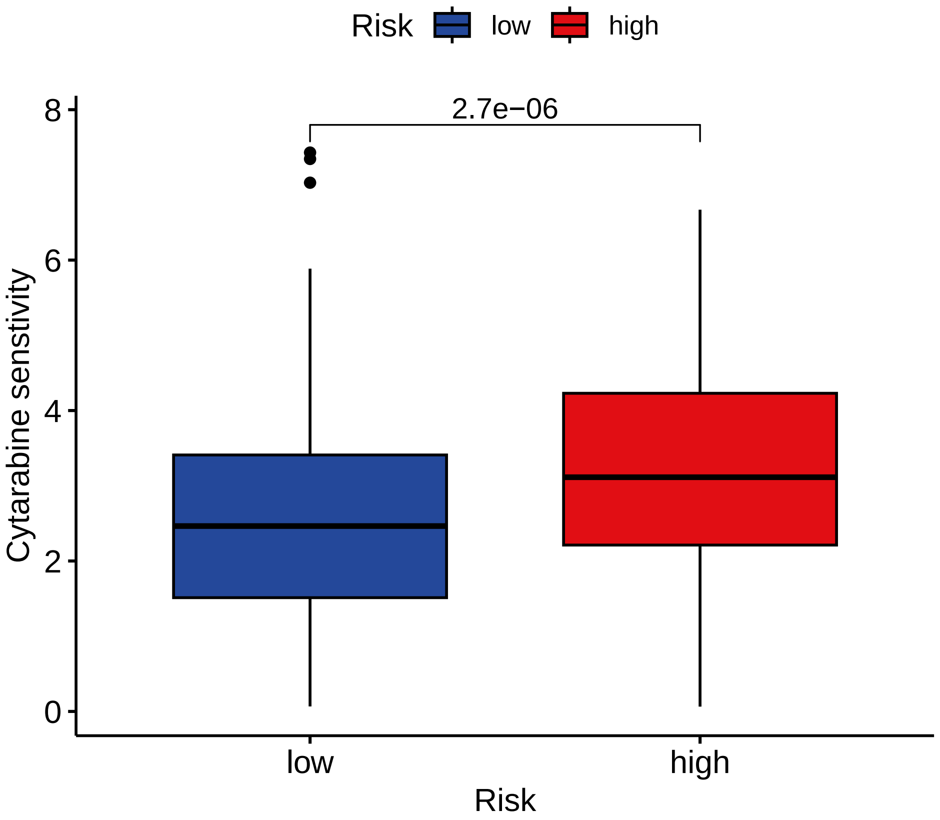

Risk
low
high
2.7e−06
8
6
4
2
0
Cytarabine senstivity
low
high
Risk

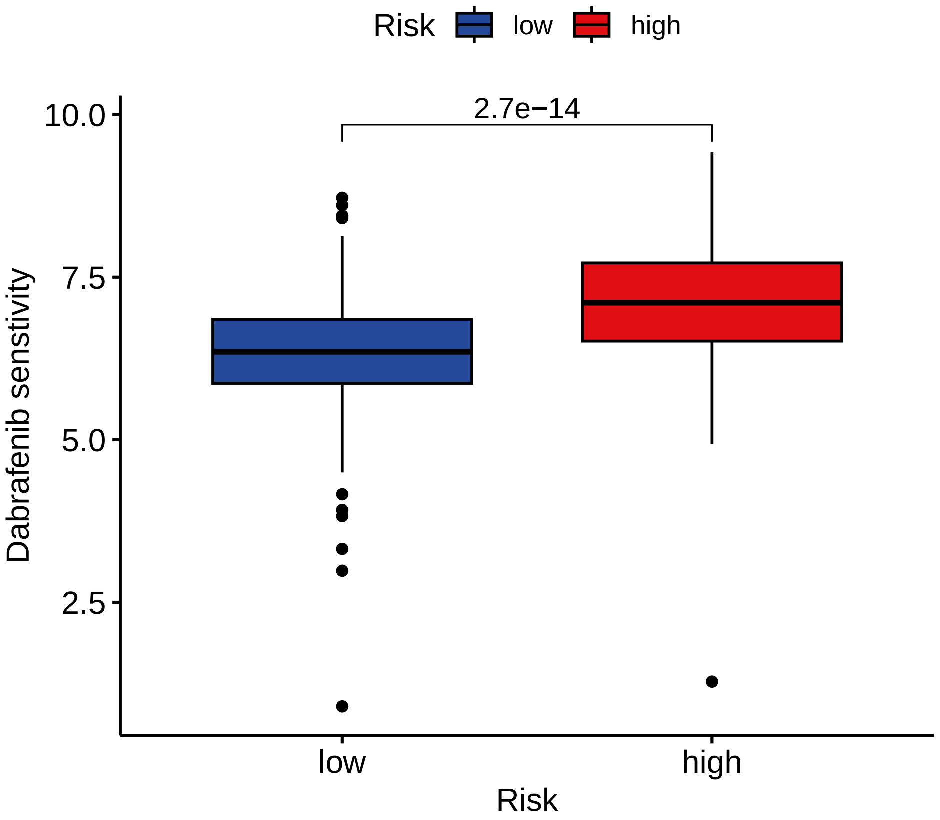

Risk
low
high
2.7e−14
10.0
7.5
5.0
2.5
Dabrafenib senstivity
low
high
Risk

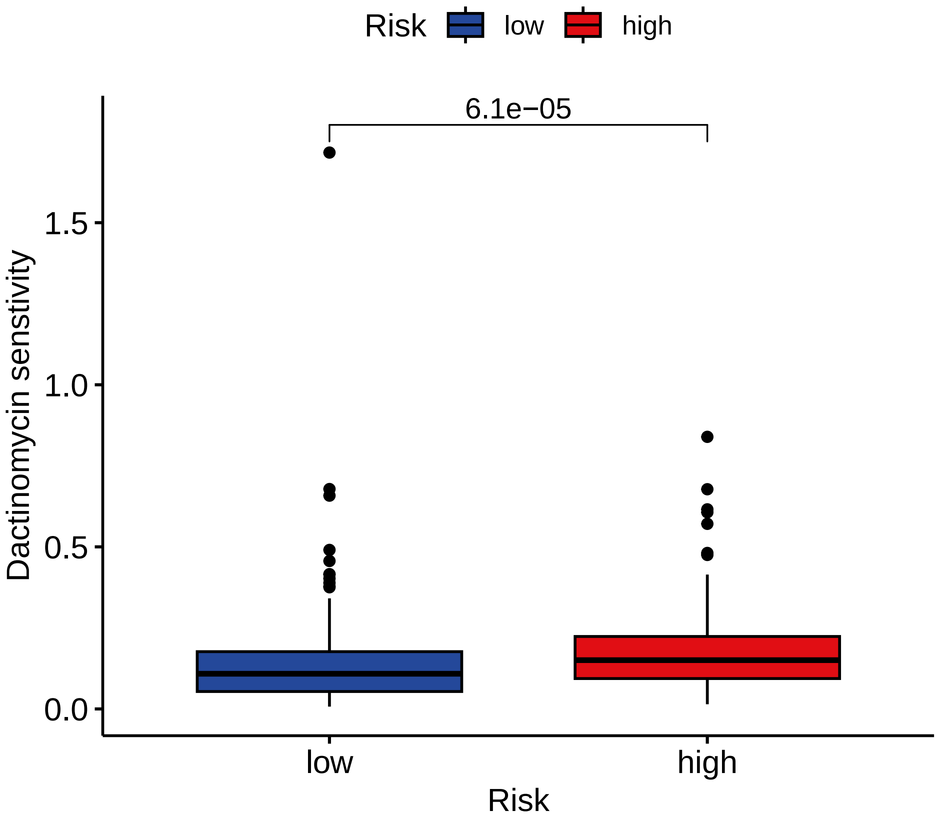

Risk
low
high
6.1e−05
Dactinomycin senstivity
1.5
1.0
0.5
0.0
low
high
Risk

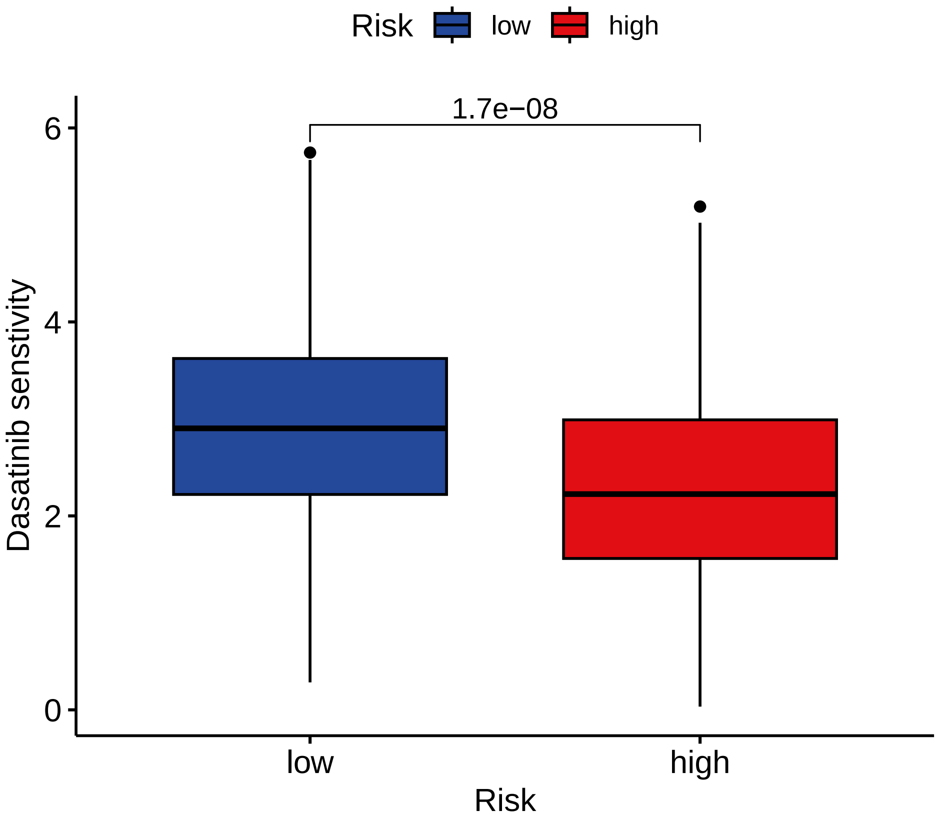
Risk
low
high
1.7e−08
6
4
2
0
Dasatinib senstivity
low
high
Risk

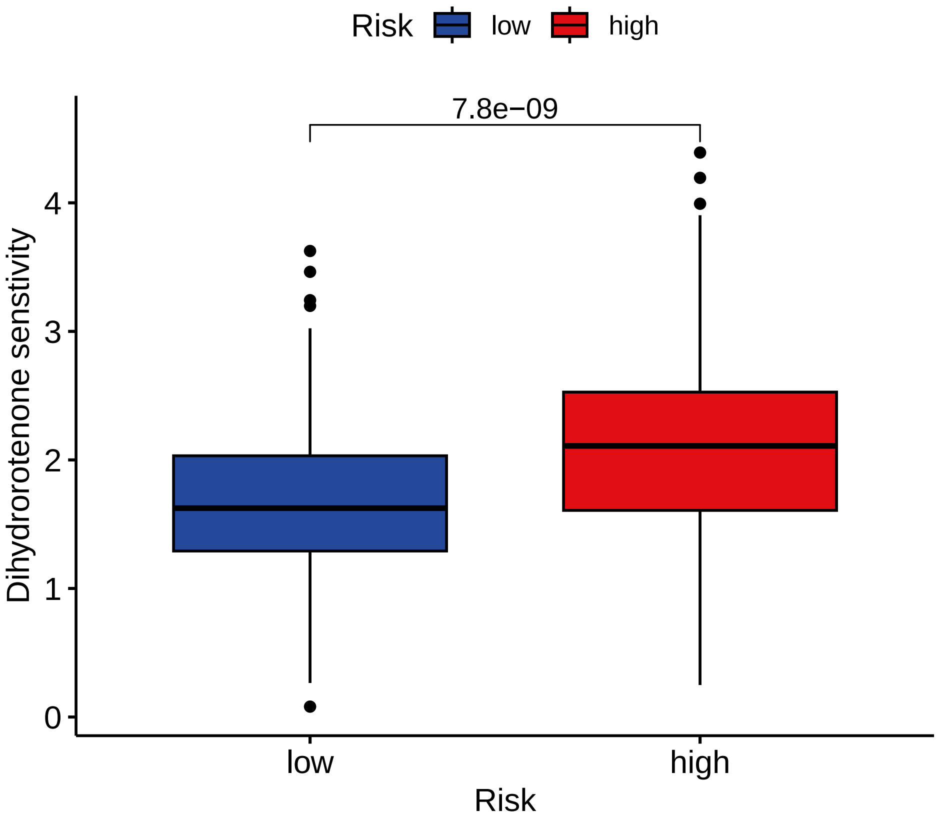

Risk
low
high
7.8e−09
Dihydrorotenone senstivity
0
1
2
3
4
low
high
Risk

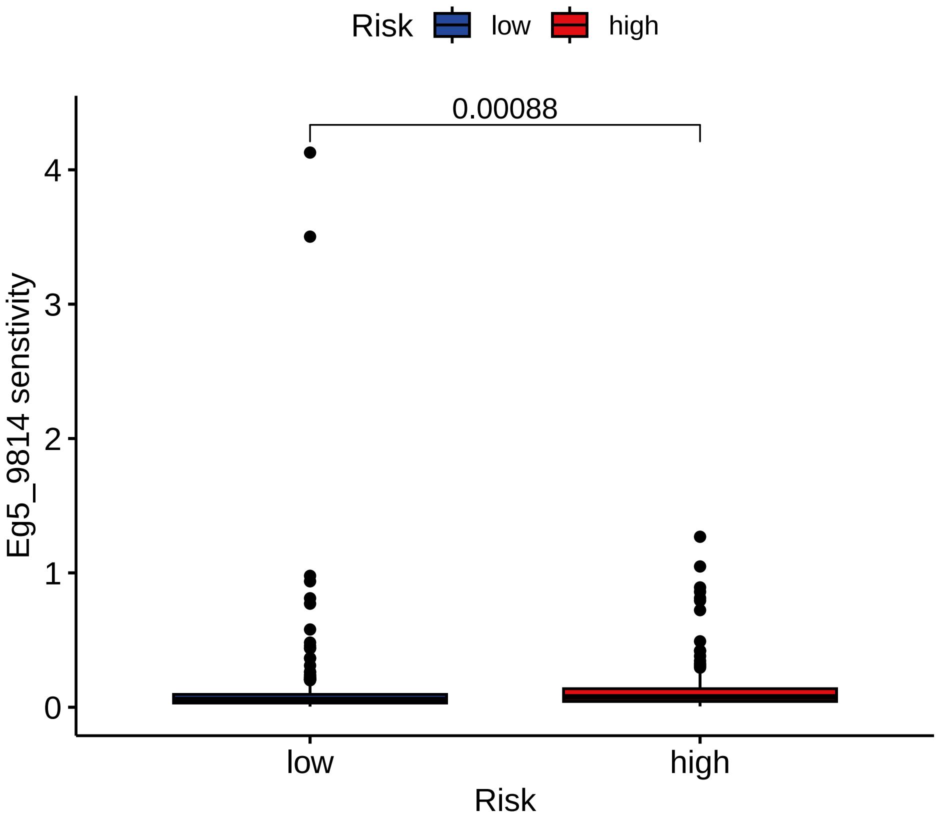

Risk
low
high
0.00088
Eg5_9814 senstivity
0
1
2
3
4
low
high
Risk

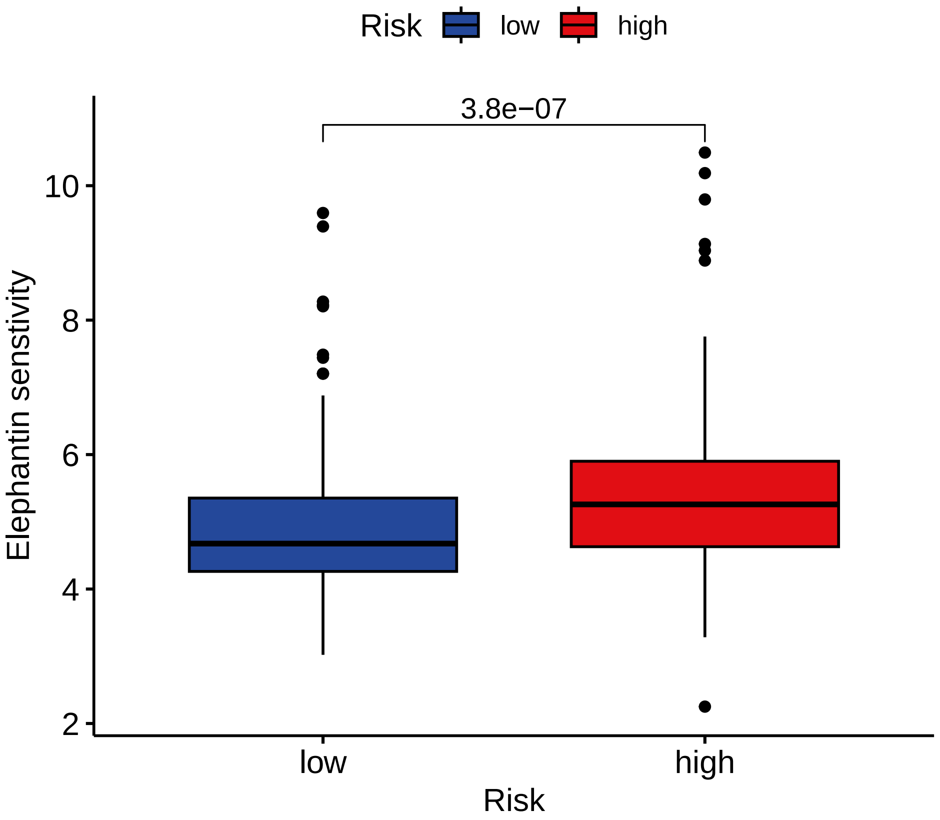
Risk
low
high
3.8e−07
Elephantin senstivity
10
8
6
4
2
low
high
Risk

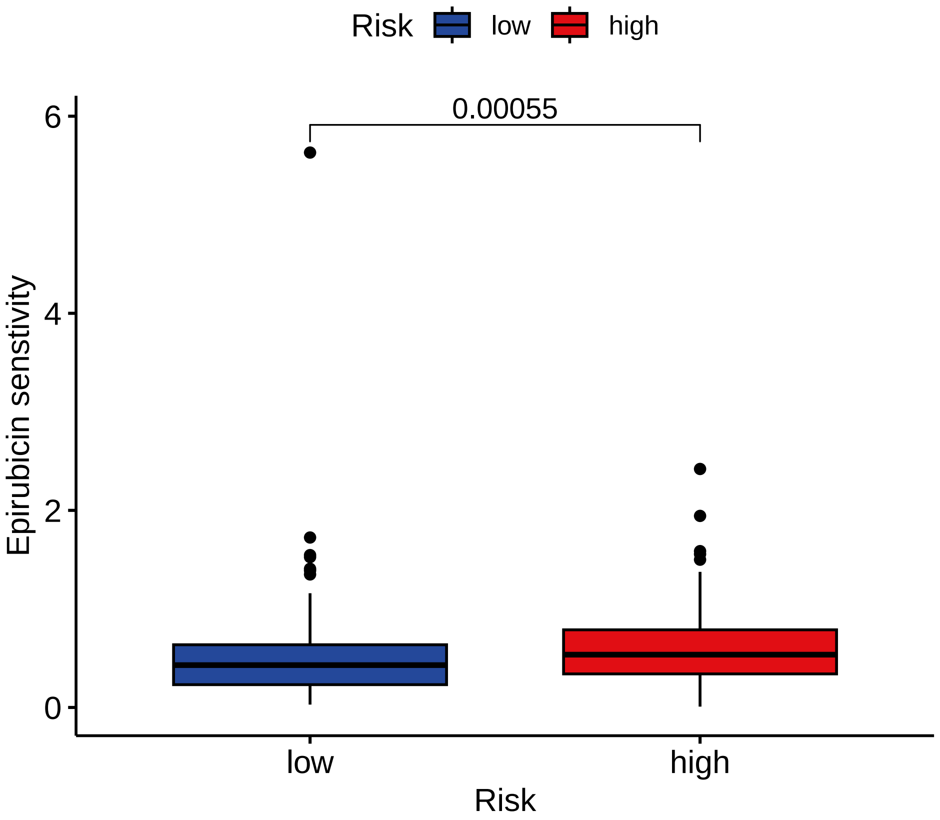
Risk
low
high
0.00055
6
4
2
0
Epirubicin senstivity
low
high
Risk

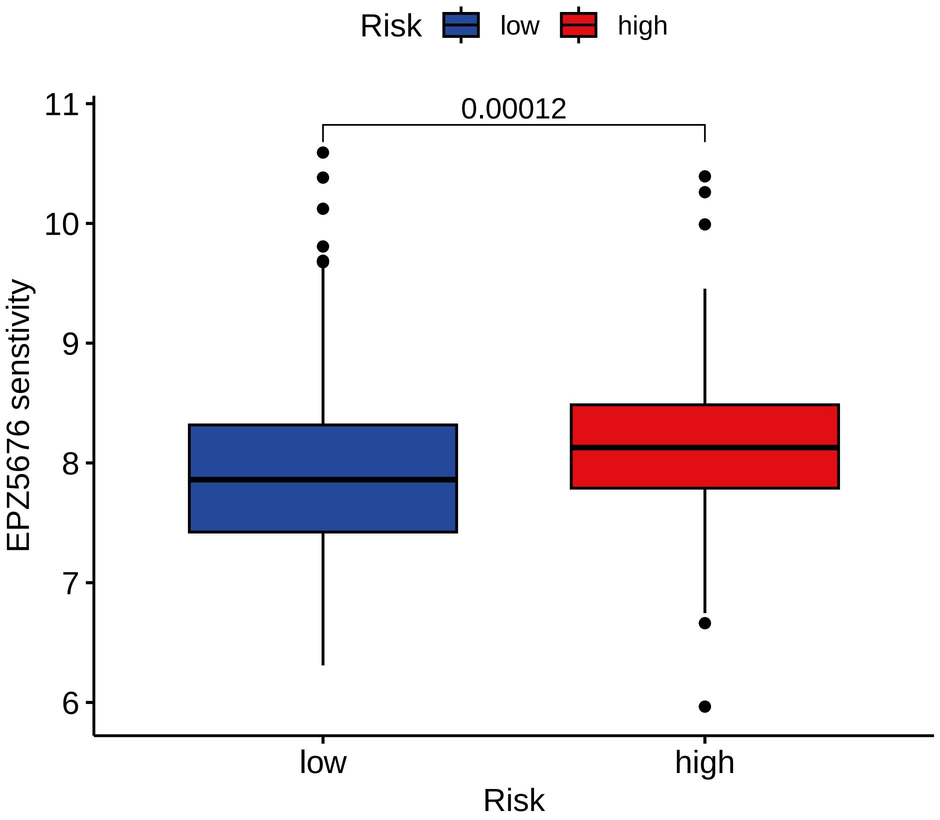

Risk
low
high
0.00012
11
10
9
8
7
6
EPZ5676 senstivity
low
high
Risk

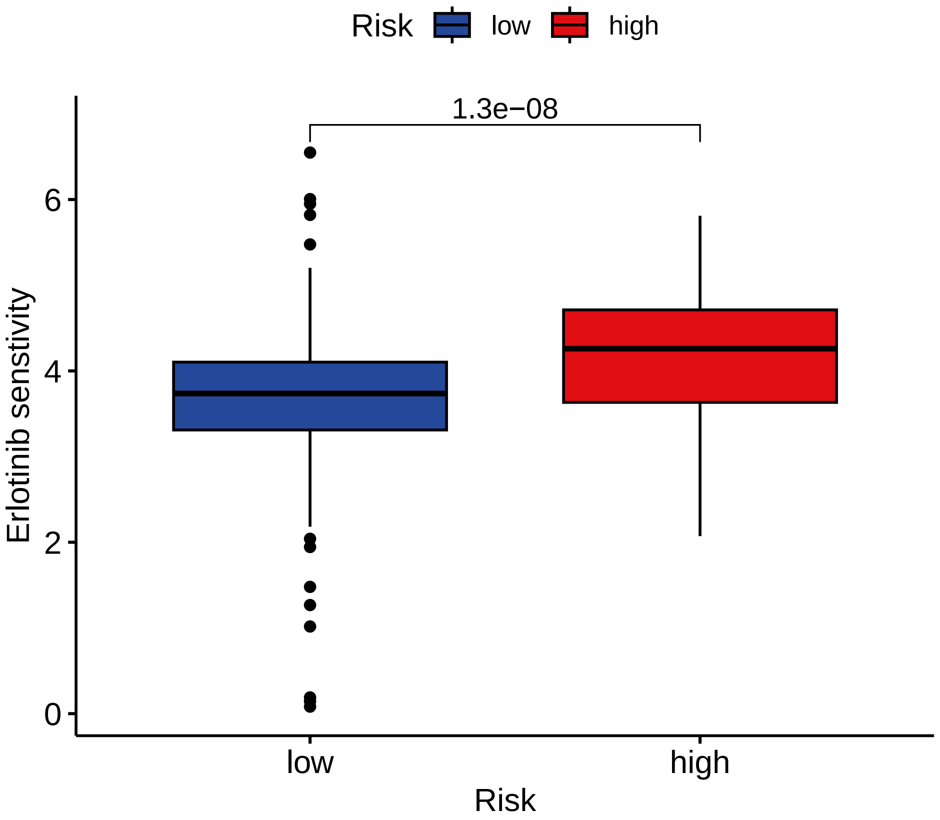
Risk
low
high
1.3e−08
Erlotinib senstivity
6
4
2
0
low
high
Risk

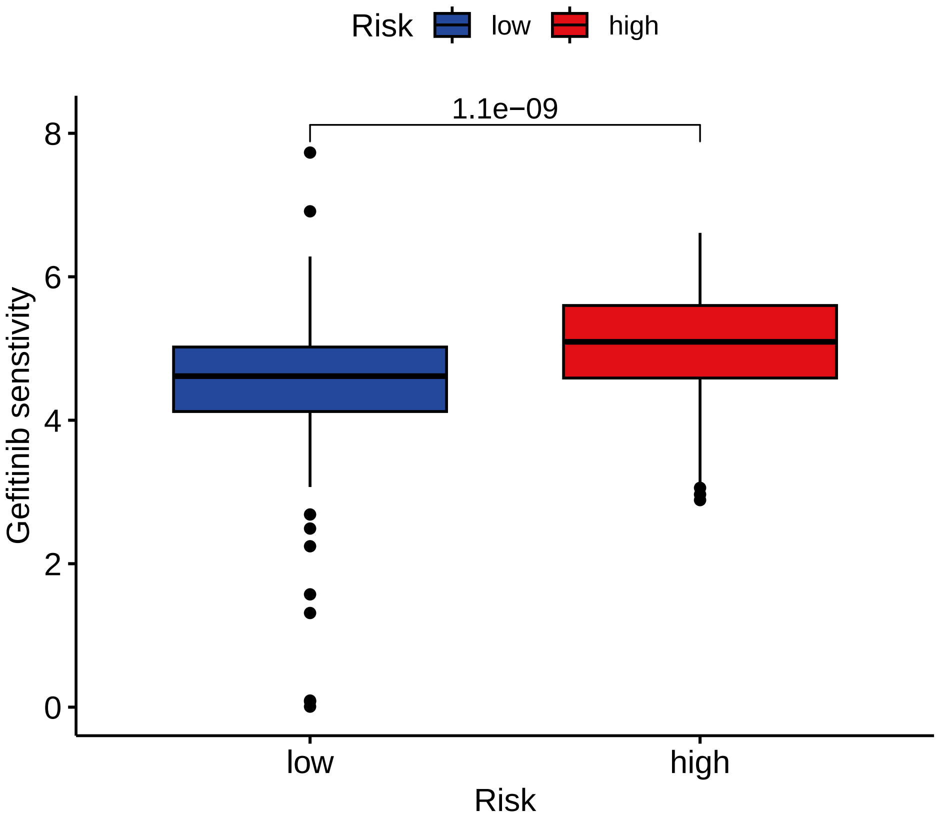
Risk
low
high
1.1e−09
Gefitinib senstivity
8
6
4
2
0
low
high
Risk

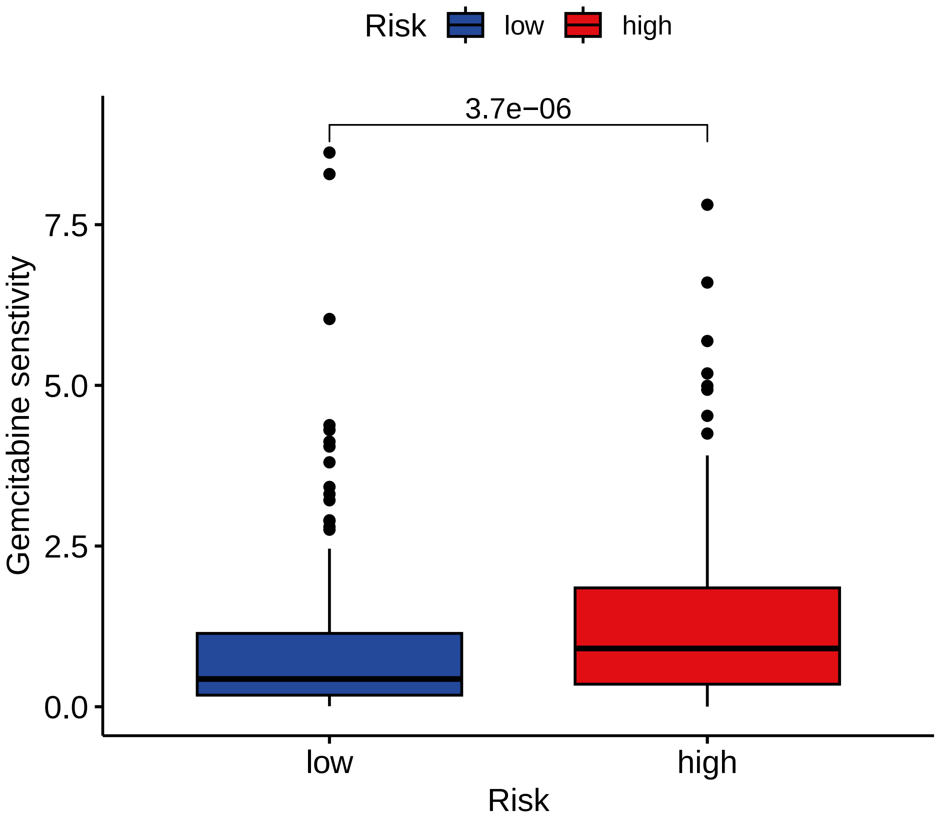
Risk
low
high
3.7e−06
Gemcitabine senstivity
7.5
5.0
2.5
0.0
low
high
Risk

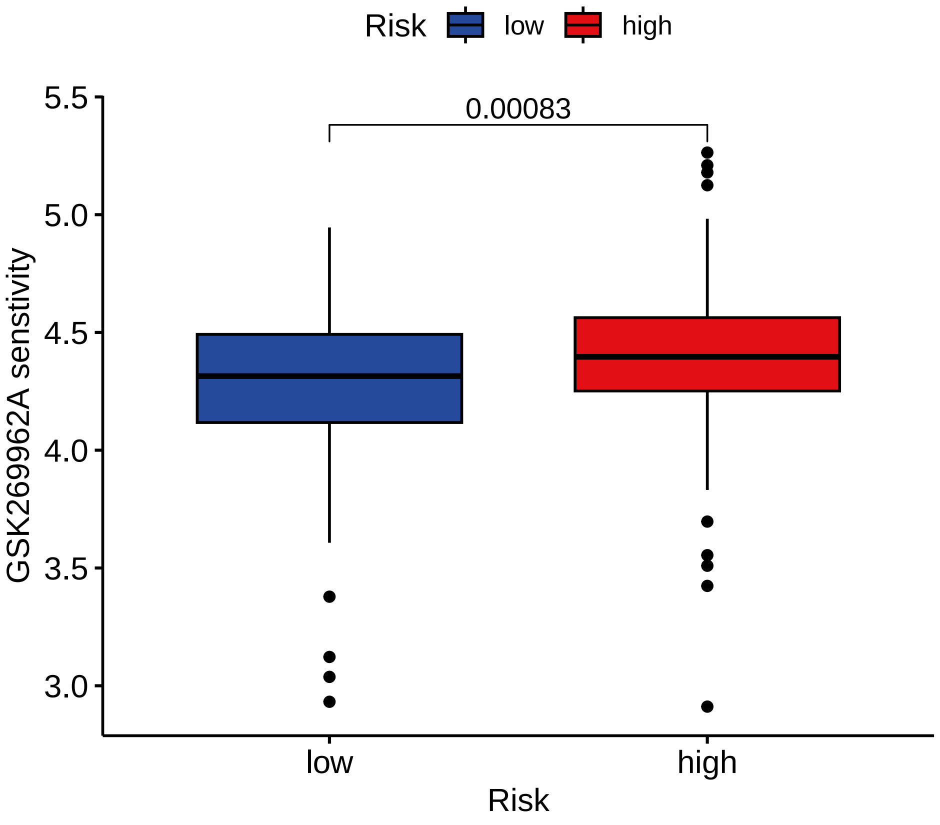

Risk
low
high
0.00083
5.5
5.0
4.5
4.0
3.5
3.0
GSK269962A senstivity
low
high
Risk

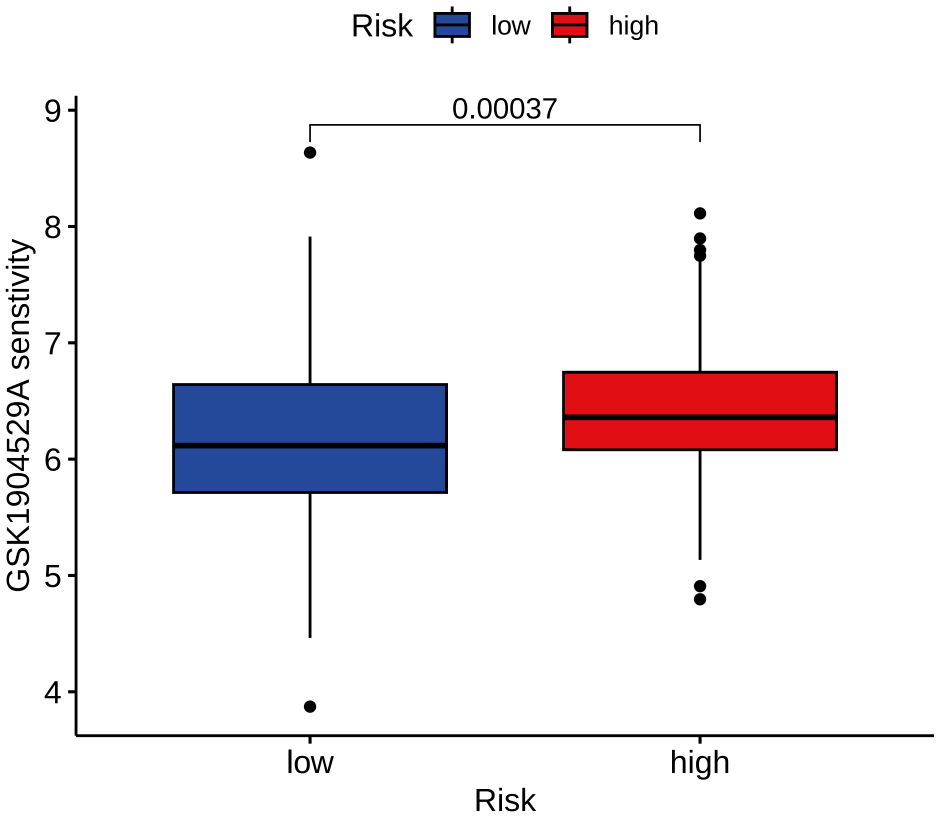

Risk
low
high
0.00037
GSK1904529A senstivity
9
8
7
6
5
4
low
high
Risk

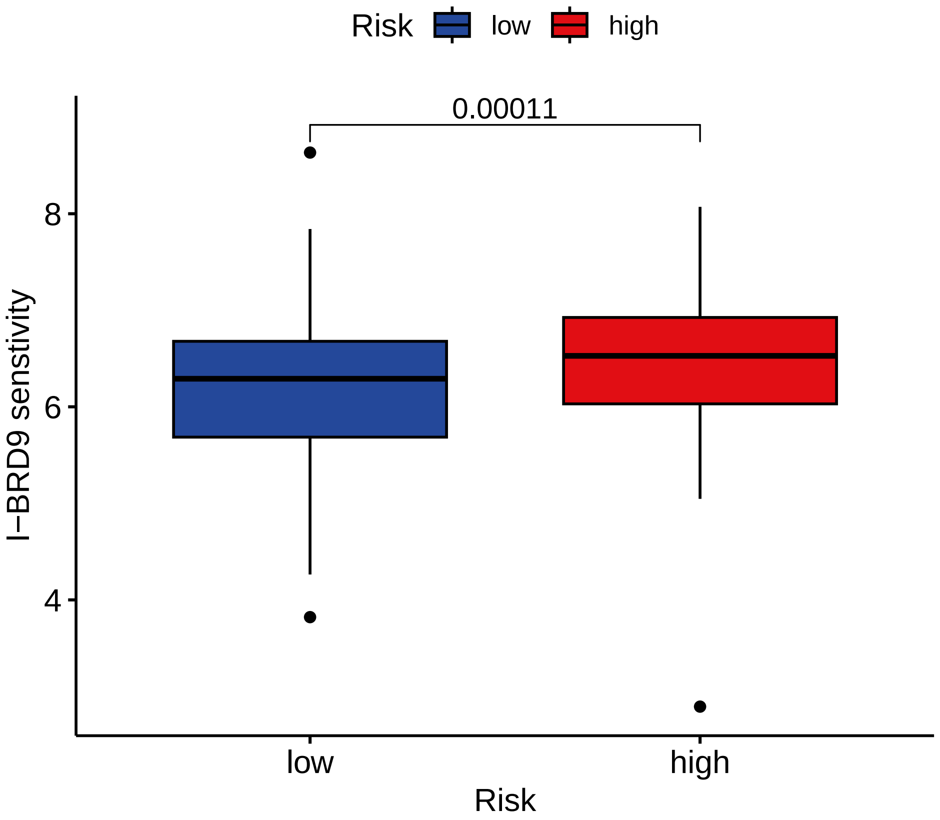

Risk
low
high
0.00011
8
6
4
I−BRD9 senstivity
low
high
Risk

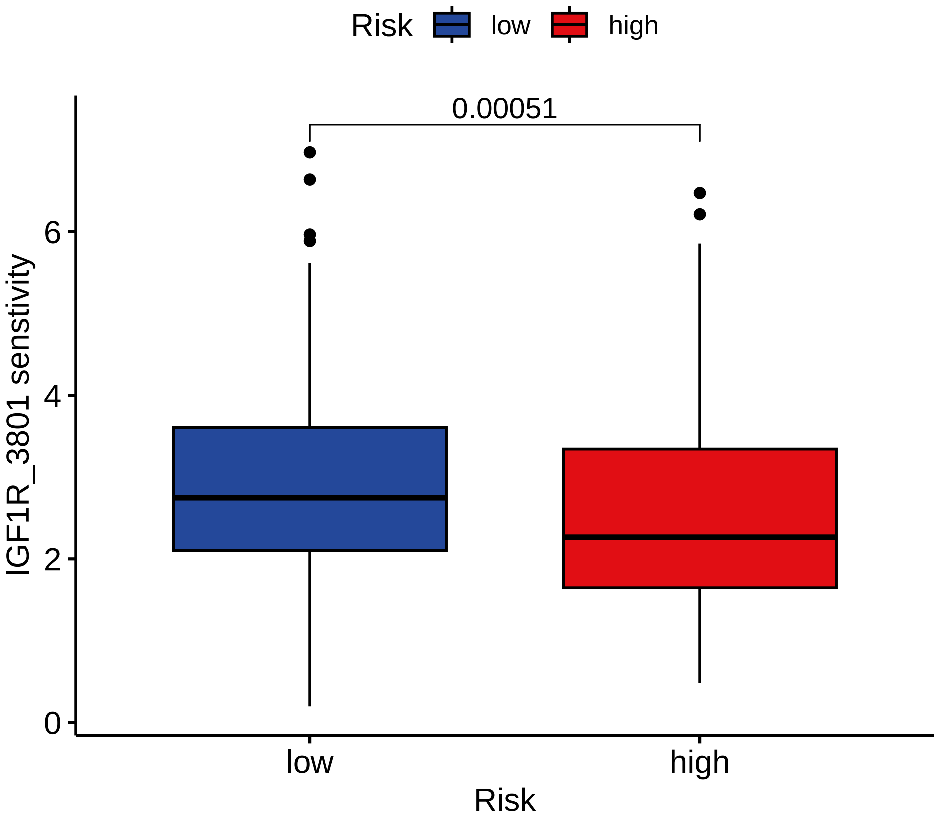

Risk
low
high
0.00051
IGF1R_3801 senstivity
6
4
2
0
low
high
Risk

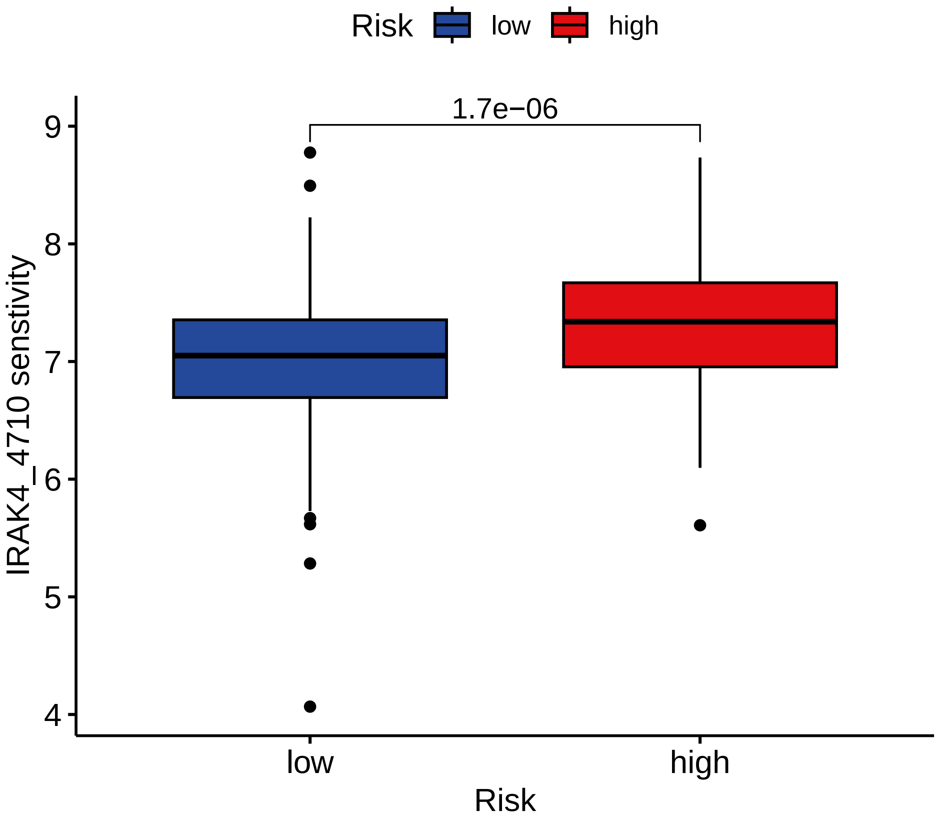
Risk
low
high
1.7e−06
IRAK4_4710 senstivity
9
8
7
6
5
4
low
high
Risk

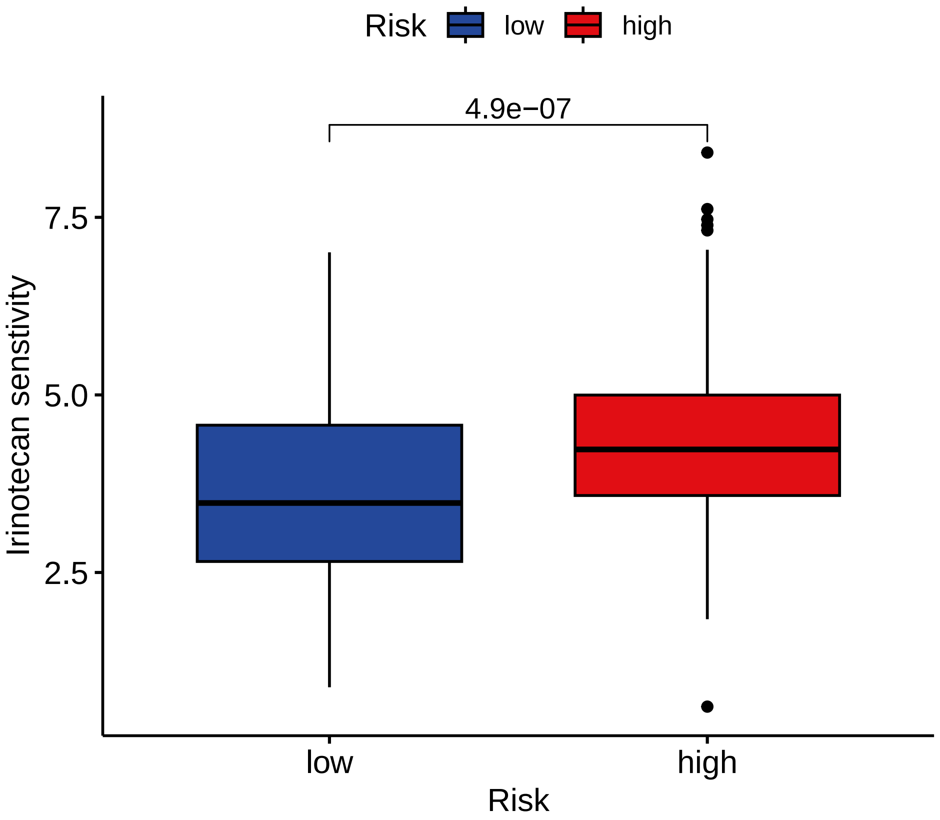

Risk
low
high
4.9e−07
7.5
5.0
2.5
Irinotecan senstivity
low
high
Risk

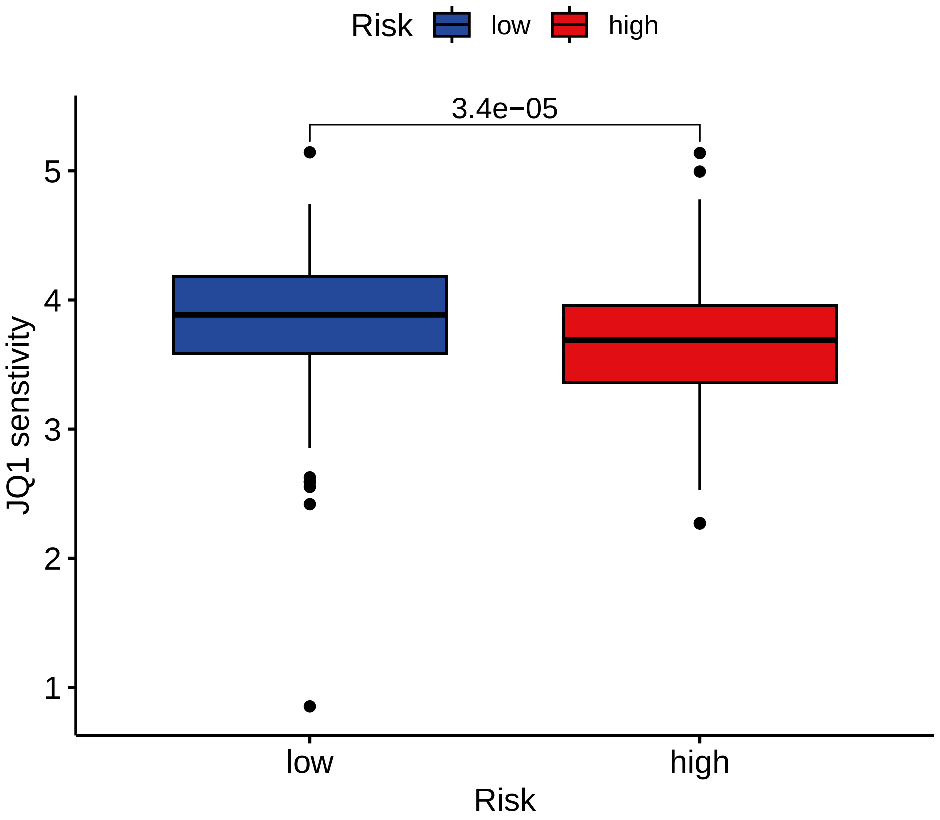
Risk
low
high
3.4e−05
JQ1 senstivity
5
4
3
2
1
low
high
Risk

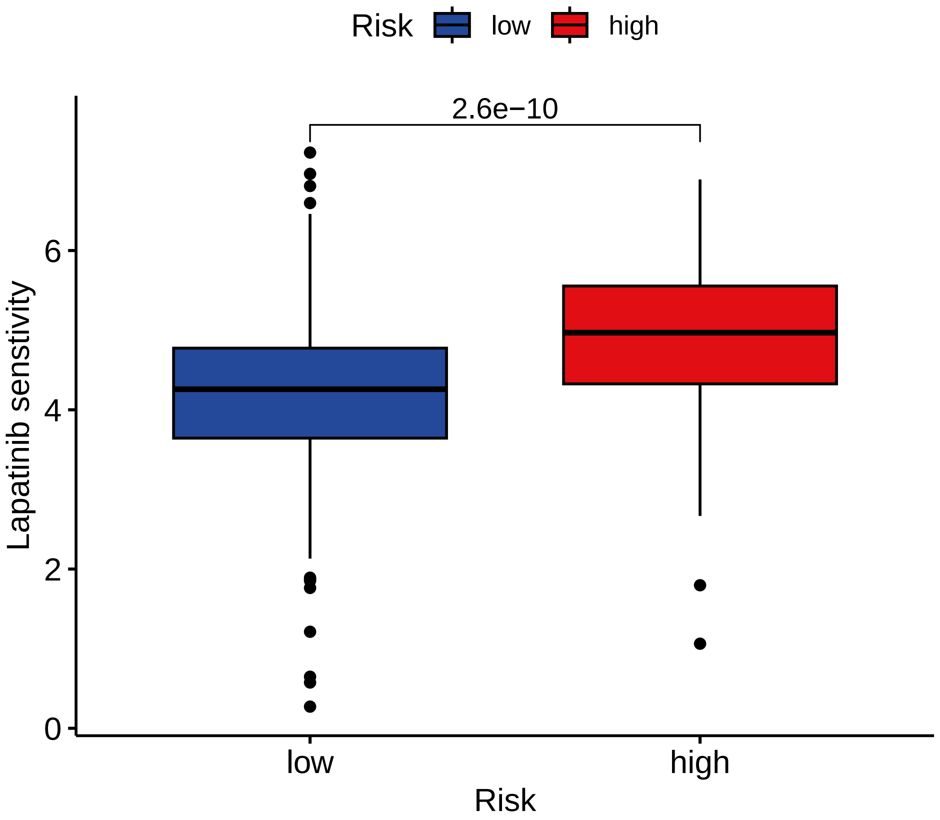

Risk
low
high
2.6e−10
Lapatinib senstivity
0
2
4
6
low
high
Risk

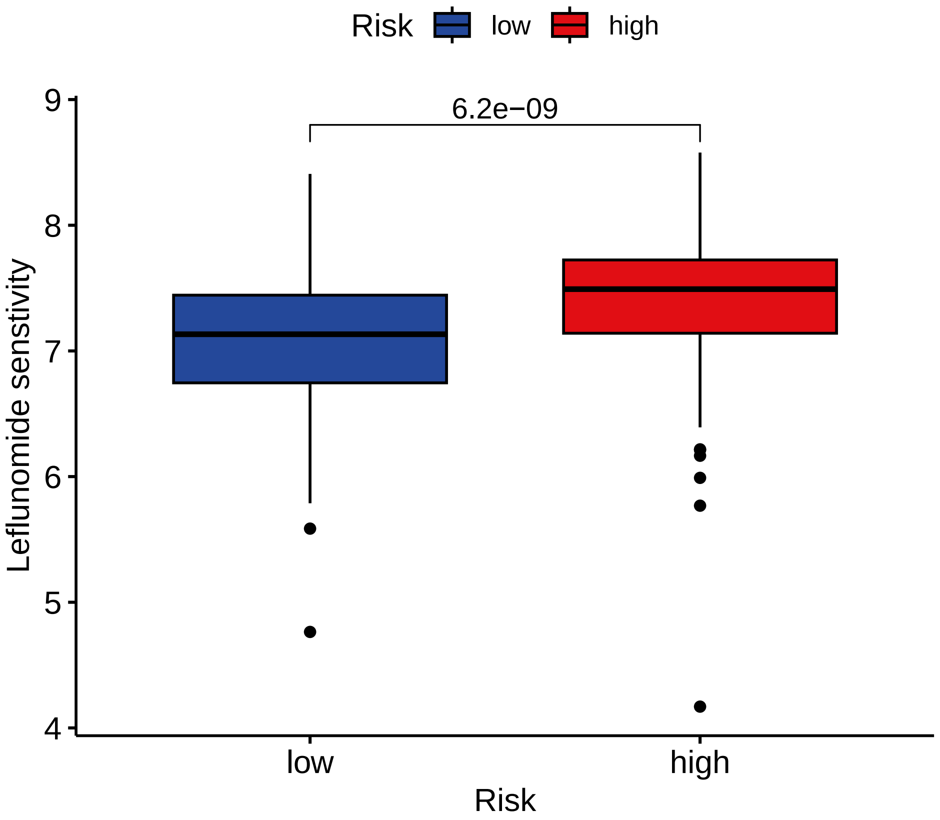

Risk
low
high
6.2e−09
9
8
7
6
5
4
Leflunomide senstivity
low
high
Risk

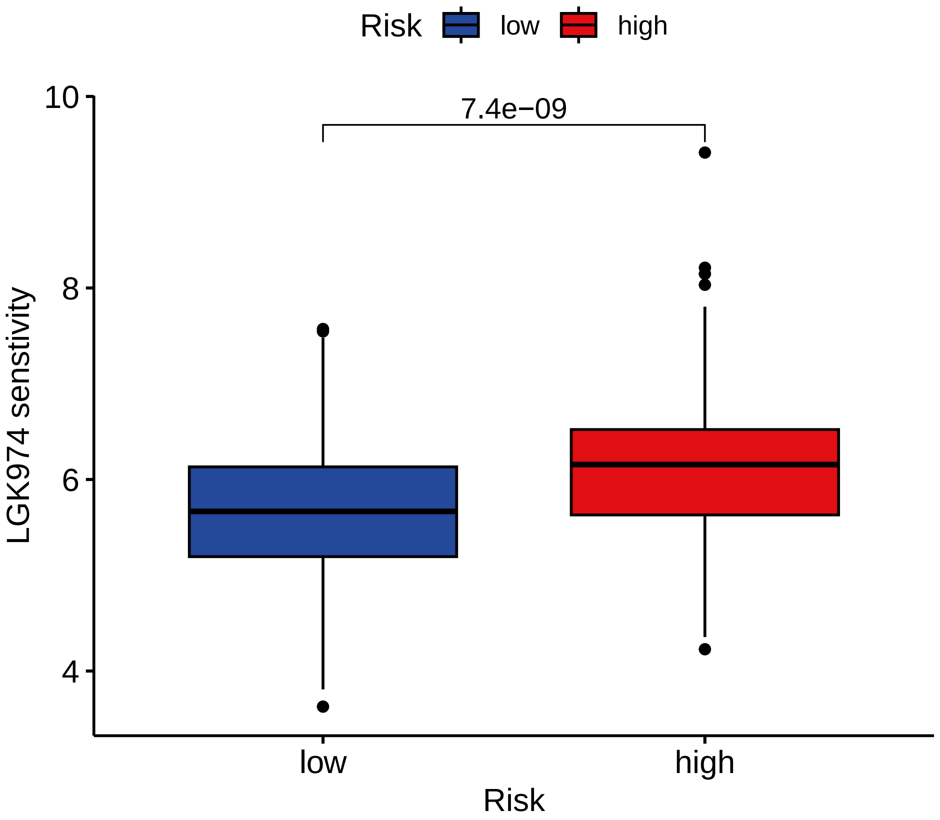

Risk
low
high
7.4e−09
10
8
6
4
LGK974 senstivity
low
high
Risk

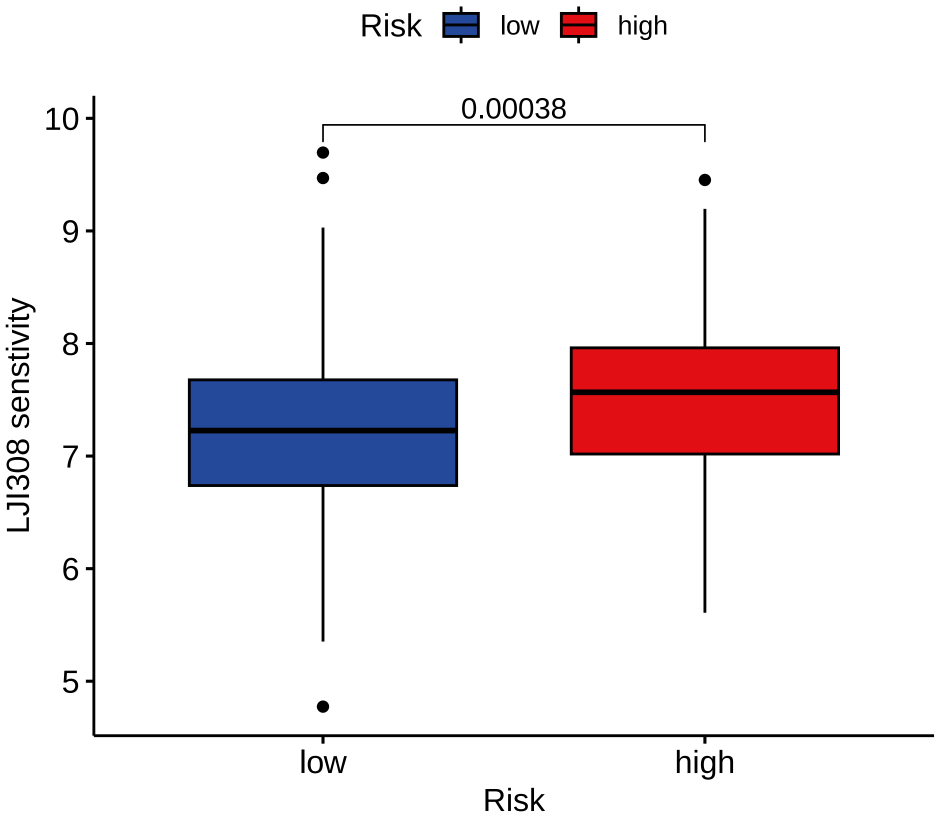

Risk
low
high
0.00038
10
9
8
7
6
5
LJI308 senstivity
low
high
Risk

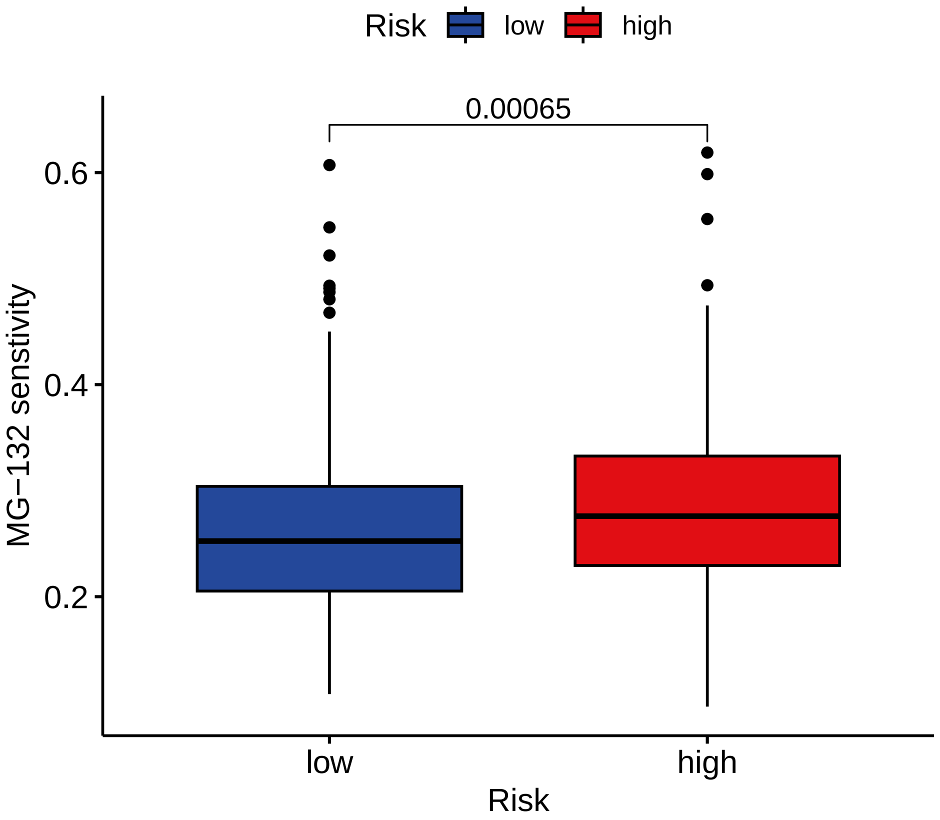
Risk
low
high
0.00065
0.6
0.4
0.2
MG−132 senstivity
low
high
Risk

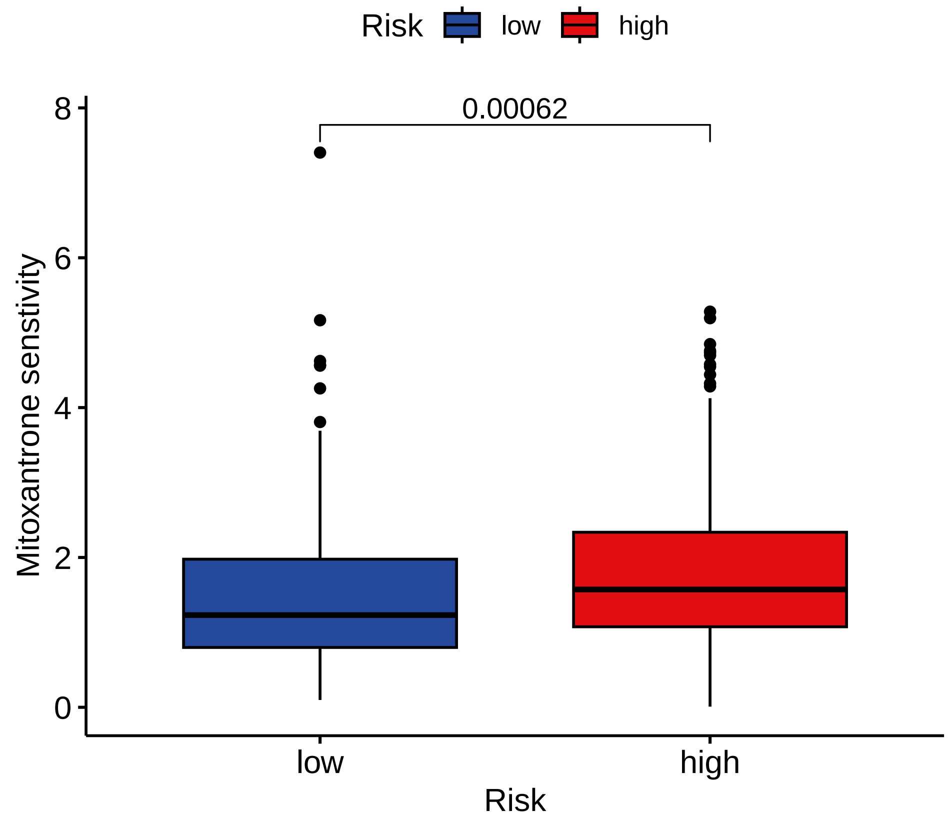
Risk
low
high
0.00062
Mitoxantrone senstivity
8
6
4
2
0
low
high
Risk

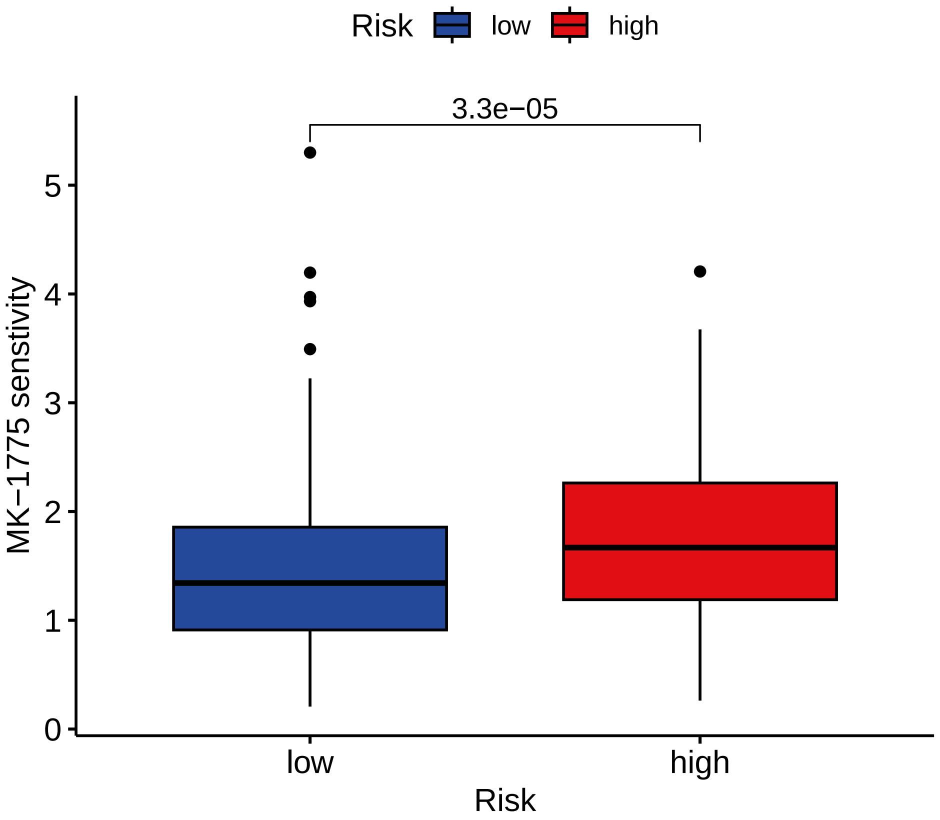

Risk
low
high
3.3e−05
MK−1775 senstivity
0
1
2
3
4
5
low
high
Risk

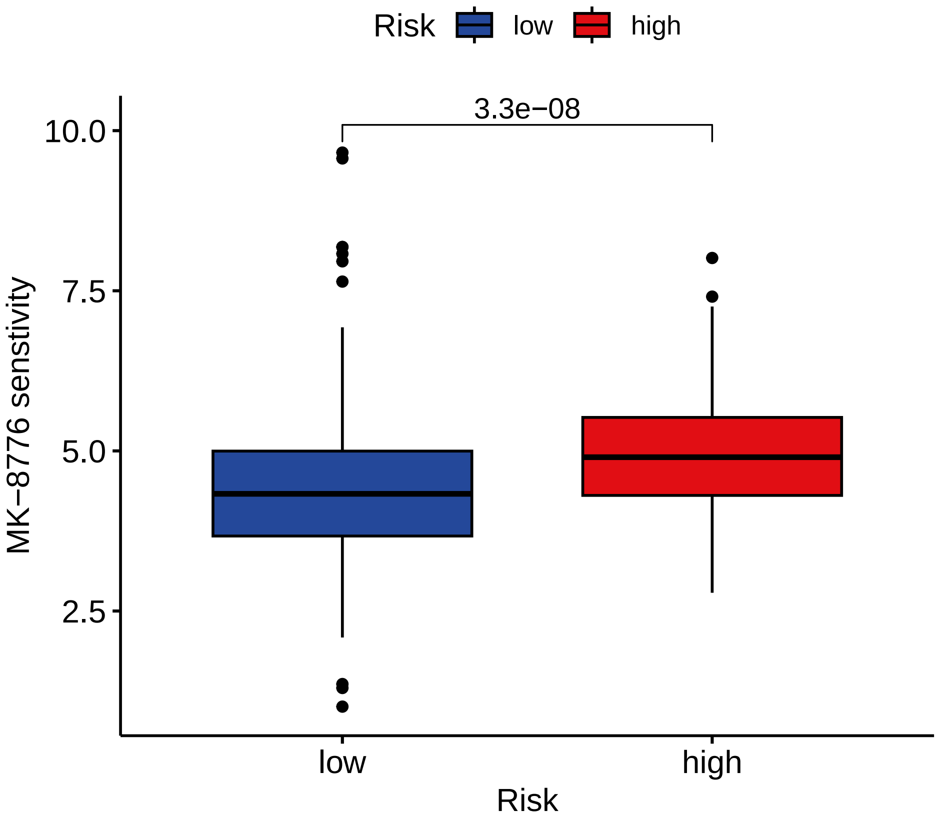
Risk
low
high
3.3e−08
10.0
7.5
5.0
2.5
MK−8776 senstivity
low
high
Risk

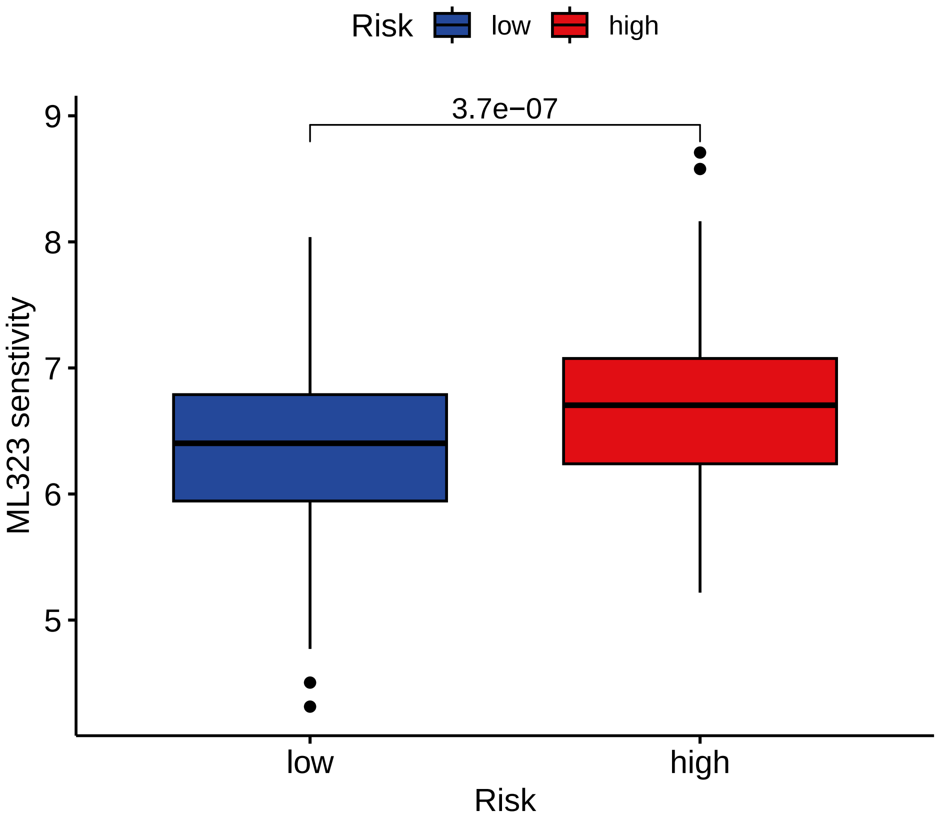
Risk
low
high
3.7e−07
9
8
7
6
5
ML323 senstivity
low
high
Risk

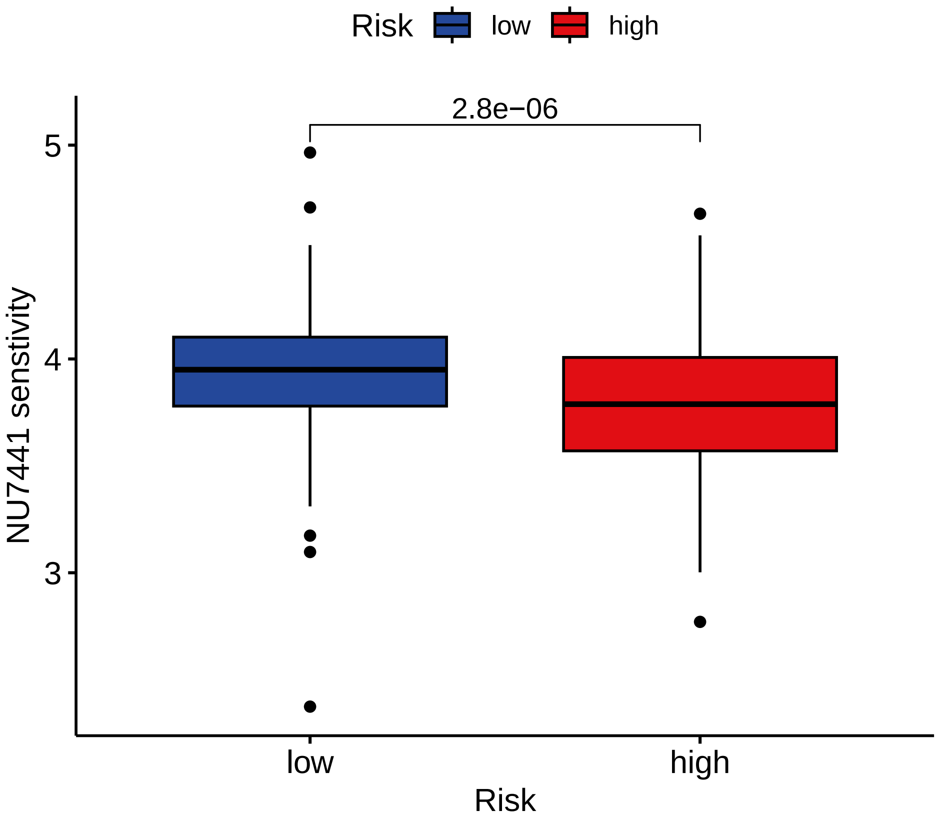

Risk
low
high
2.8e−06
5
4
3
NU7441 senstivity
low
high
Risk

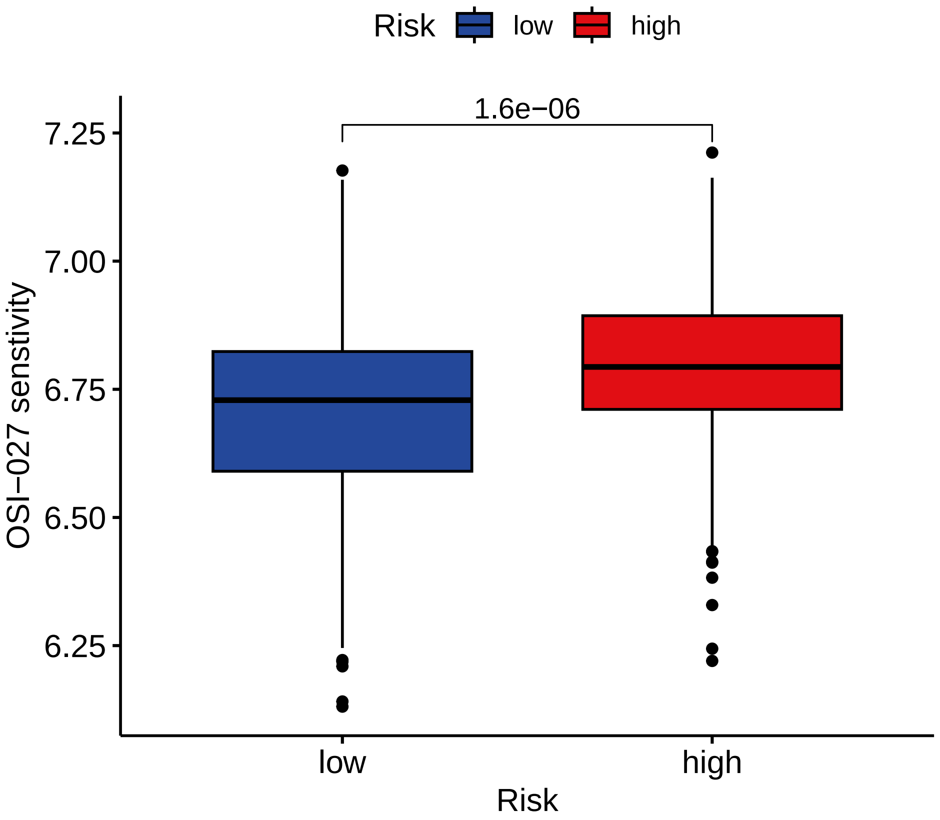
Risk
low
high
1.6e−06
7.25
7.00
6.75
6.50
6.25
OSI−027 senstivity
low
high
Risk

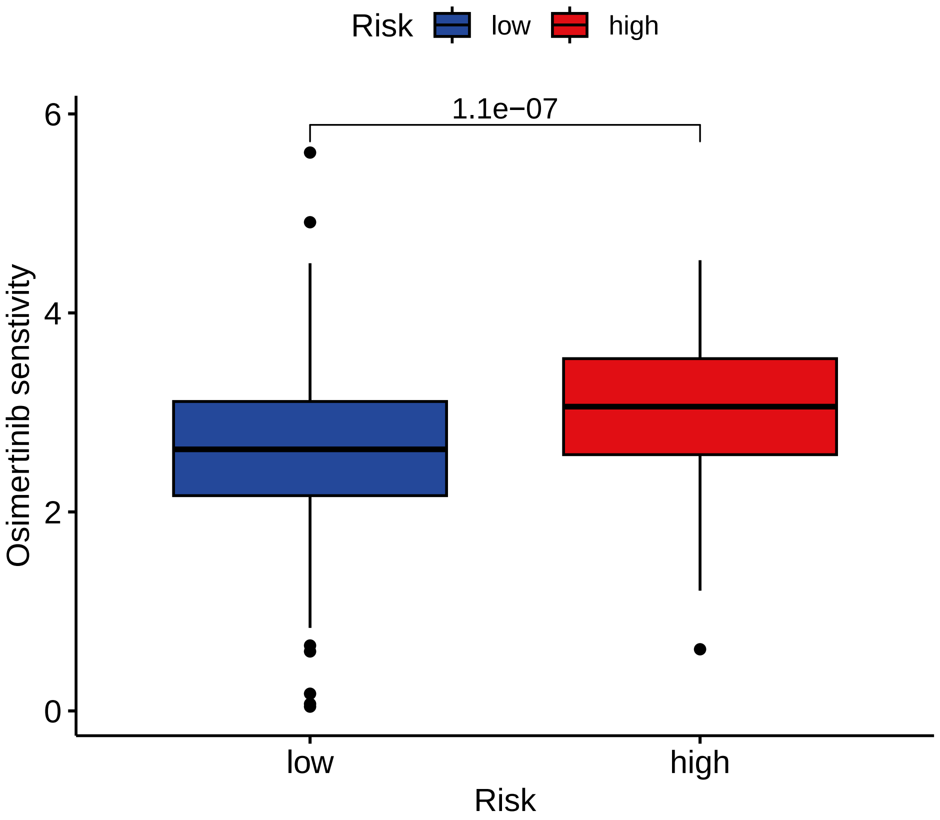
Risk
low
high
1.1e−07
Osimertinib senstivity
6
4
2
0
low
high
Risk

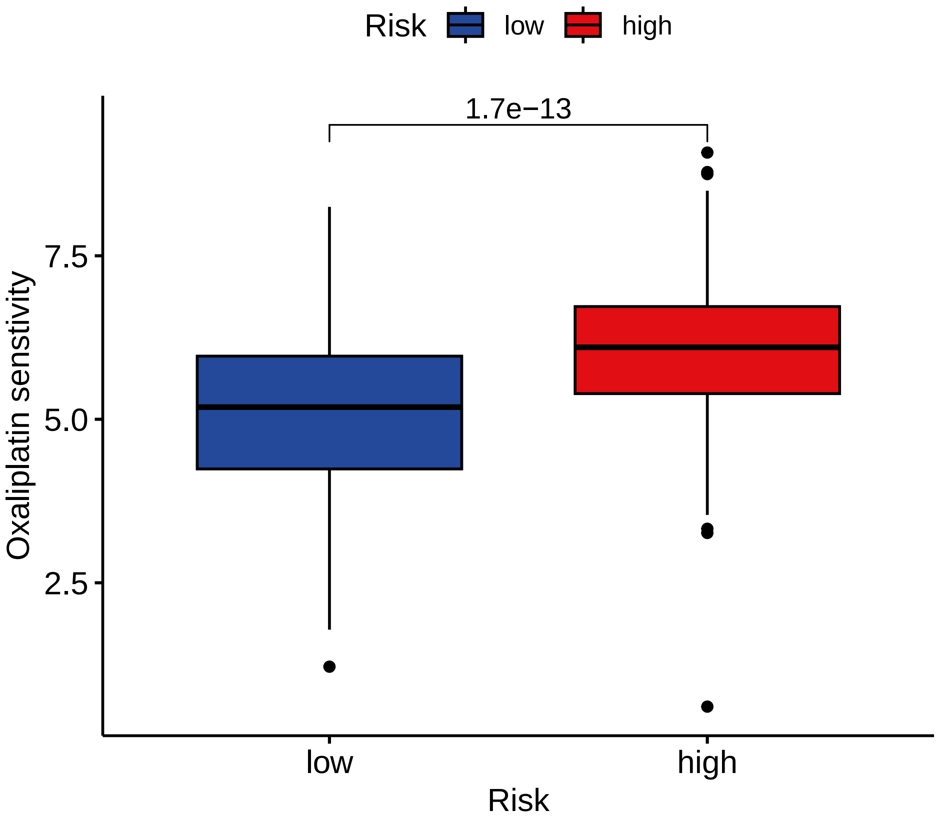

Risk
low
high
1.7e−13
Oxaliplatin senstivity
7.5
5.0
2.5
low
high
Risk

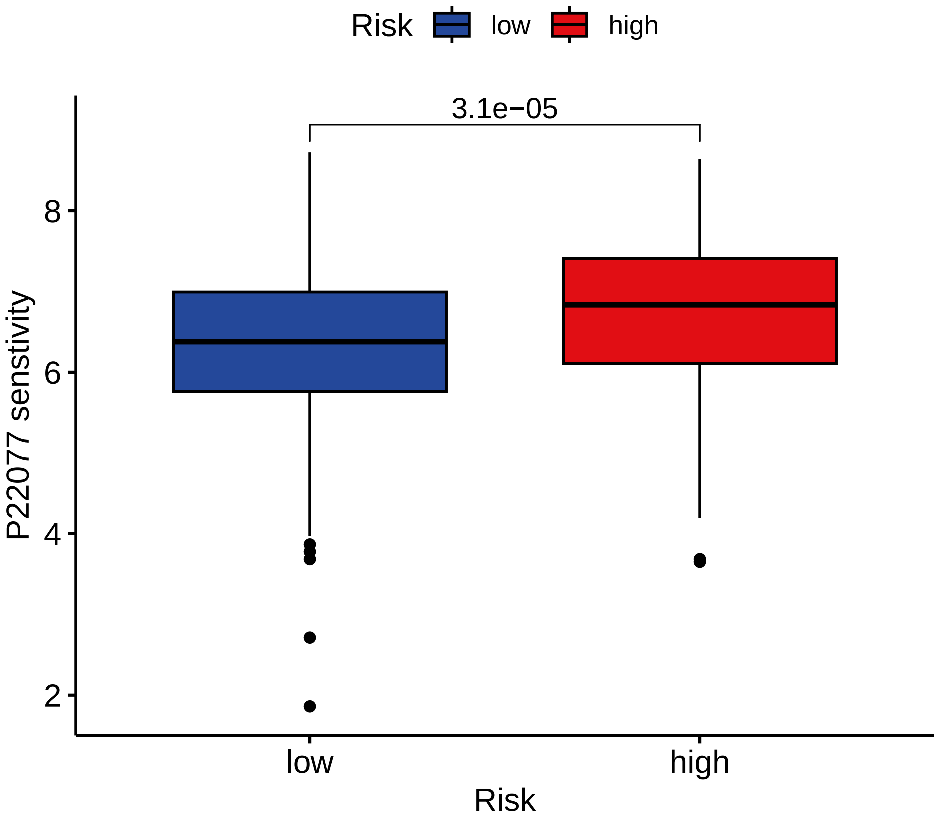

Risk
low
high
3.1e−05
P22077 senstivity
8
6
4
2
low
high
Risk

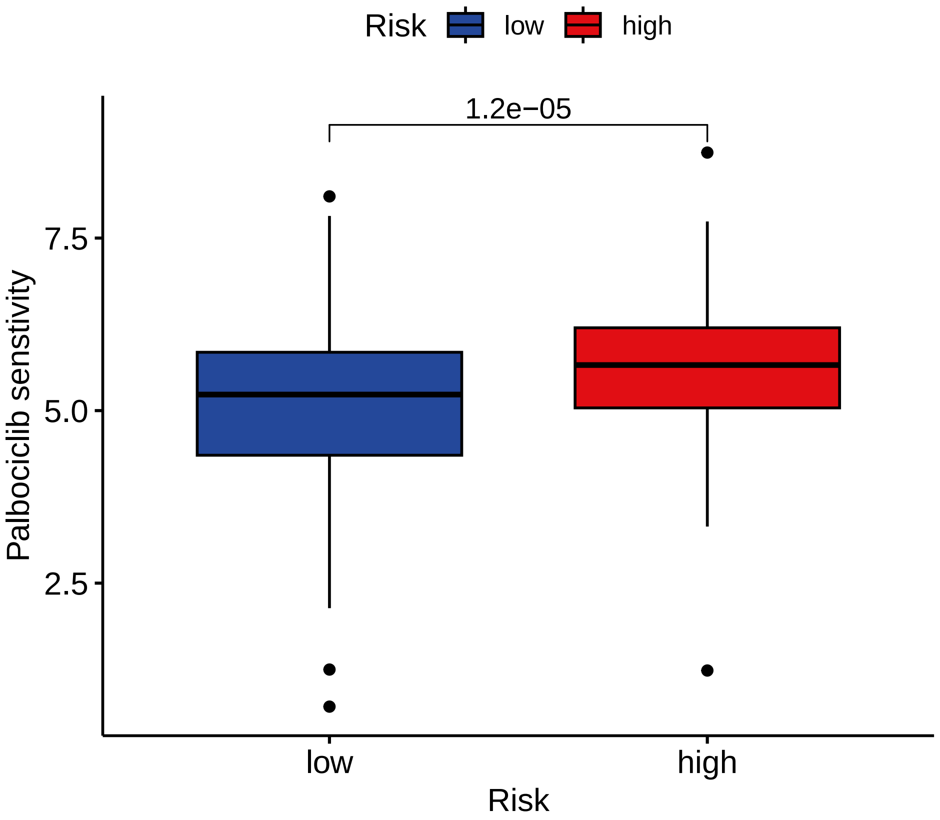

Risk
low
high
1.2e−05
Palbociclib senstivity
7.5
5.0
2.5
low
high
Risk

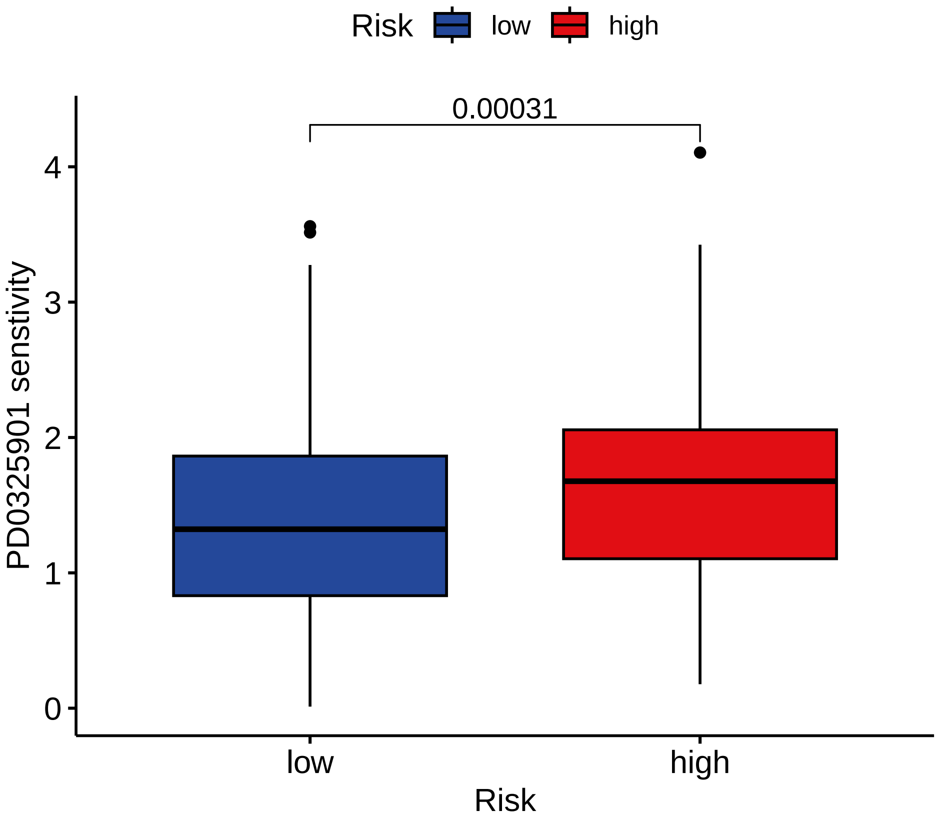

Risk
low
high
0.00031
PD0325901 senstivity
0
1
2
3
4
low
high
Risk

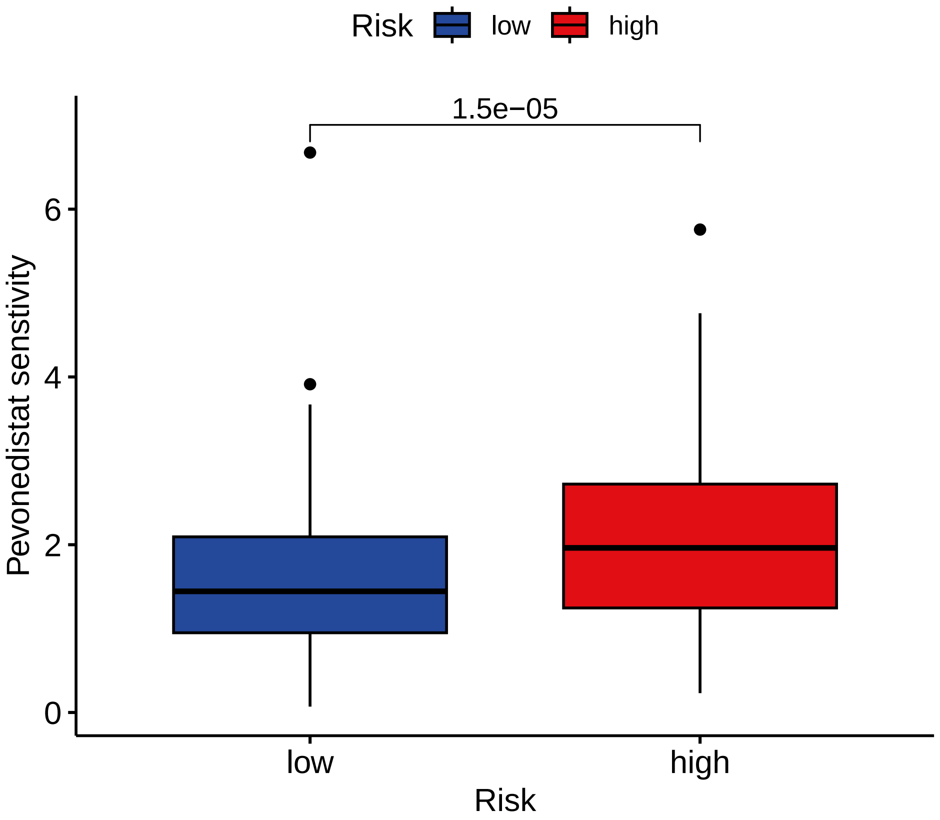
Risk
low
high
1.5e−05
Pevonedistat senstivity
6
4
2
0
low
high
Risk

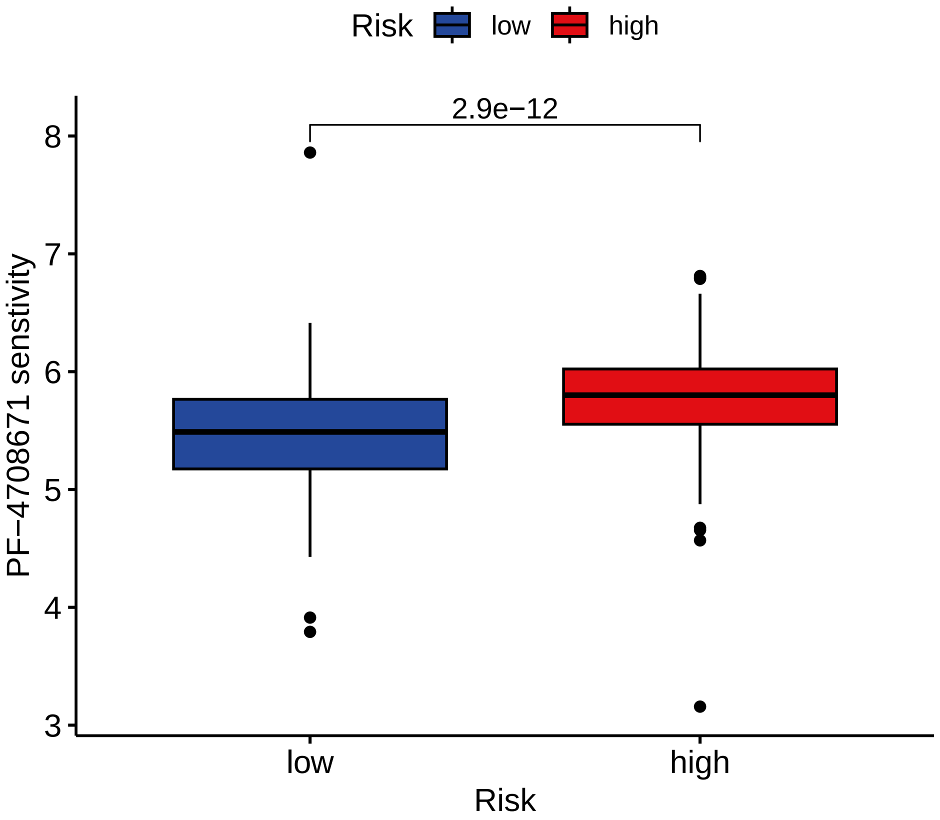

Risk
low
high
2.9e−12
PF−4708671 senstivity
8
7
6
5
4
3
low
high
Risk

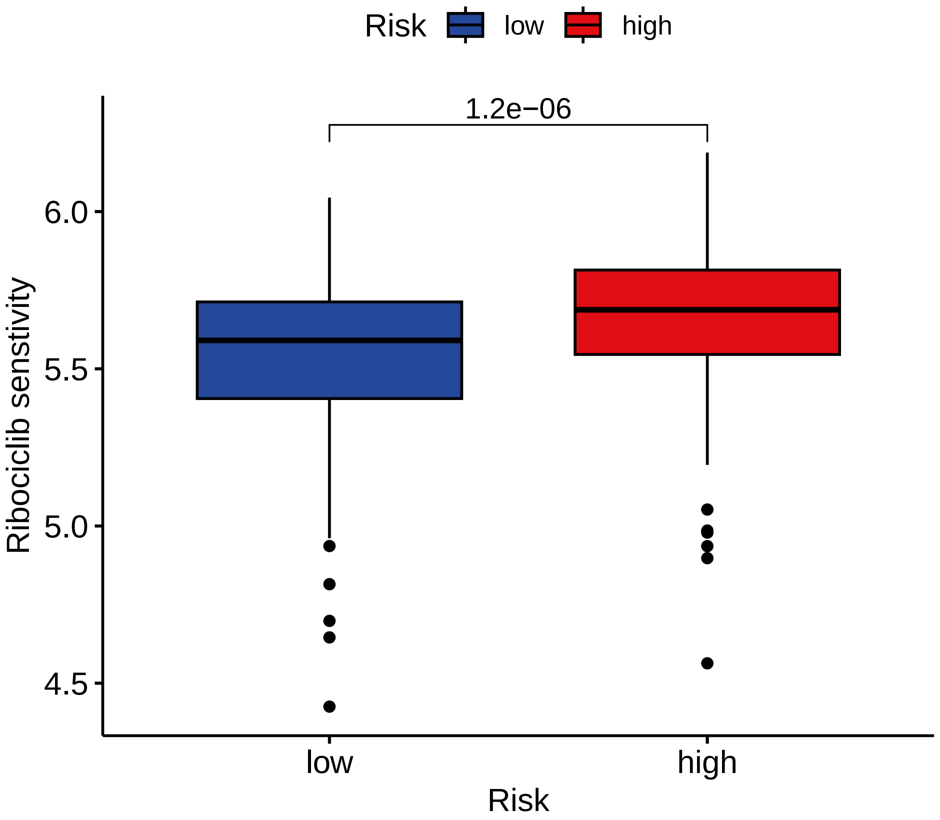

Risk
low
high
1.2e−06
6.0
5.5
5.0
4.5
Ribociclib senstivity
low
high
Risk

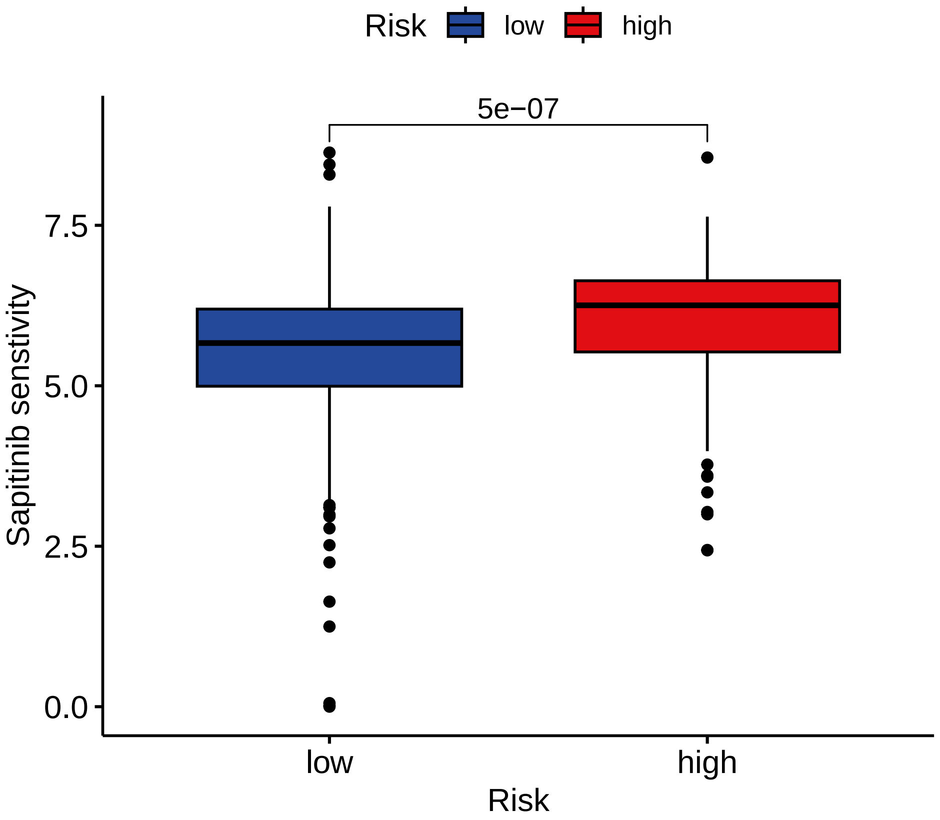

Risk
low
high
5e−07
7.5
5.0
2.5
0.0
Sapitinib senstivity
low
high
Risk

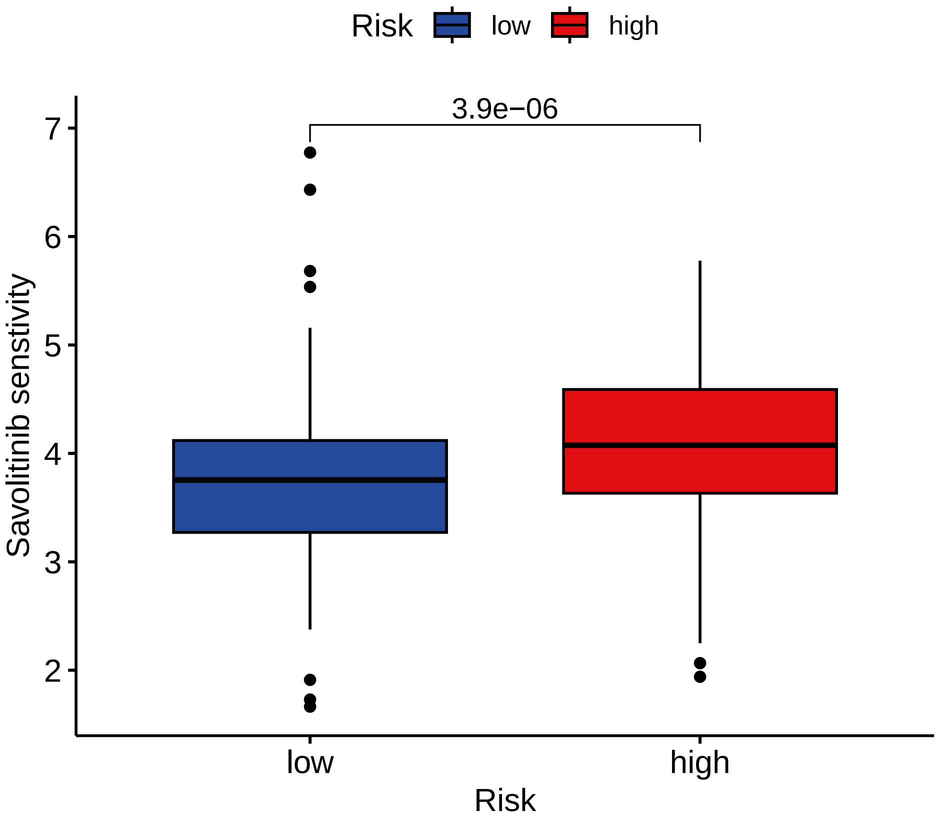

Risk
low
high
3.9e−06
Savolitinib senstivity
7
6
5
4
3
2
low
high
Risk

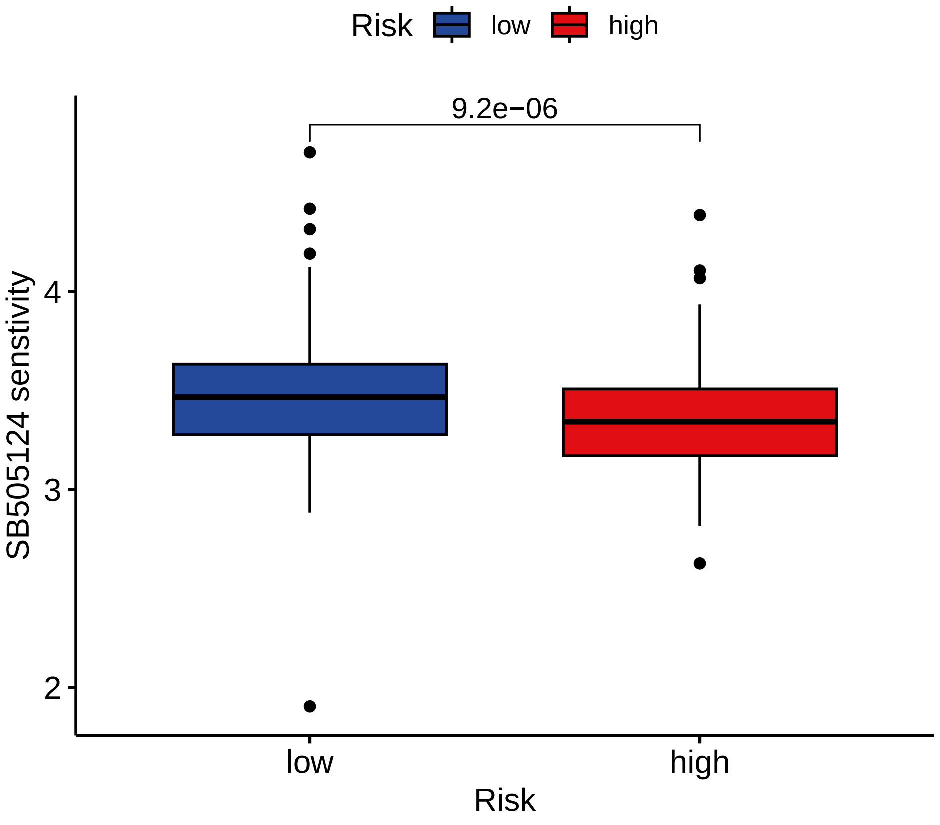

Risk
low
high
9.2e−06
SB505124 senstivity
4
3
2
low
high
Risk

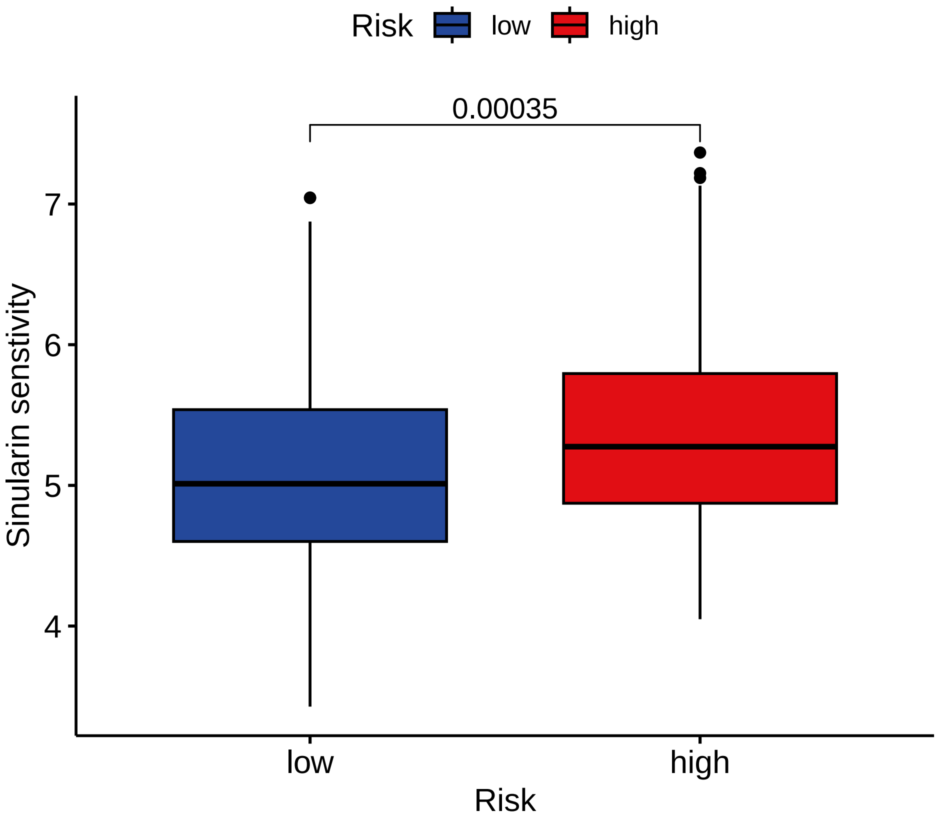
Risk
low
high
0.00035
Sinularin senstivity
7
6
5
4
low
high
Risk

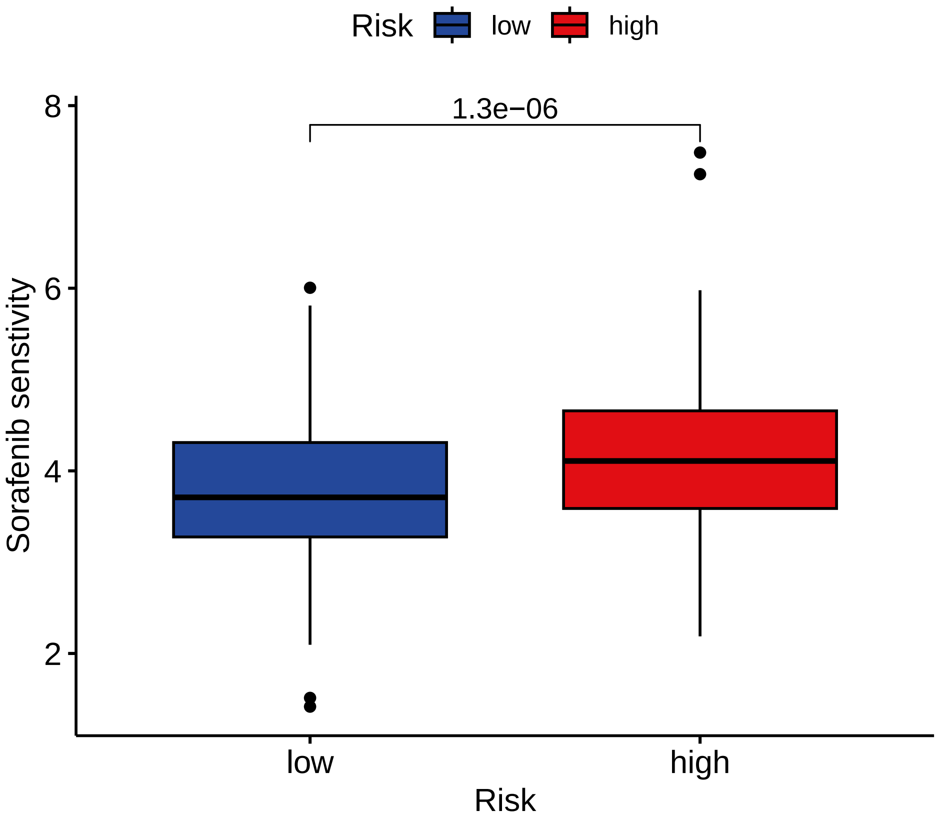
Risk
low
high
1.3e−06
8
6
4
2
Sorafenib senstivity
low
high
Risk

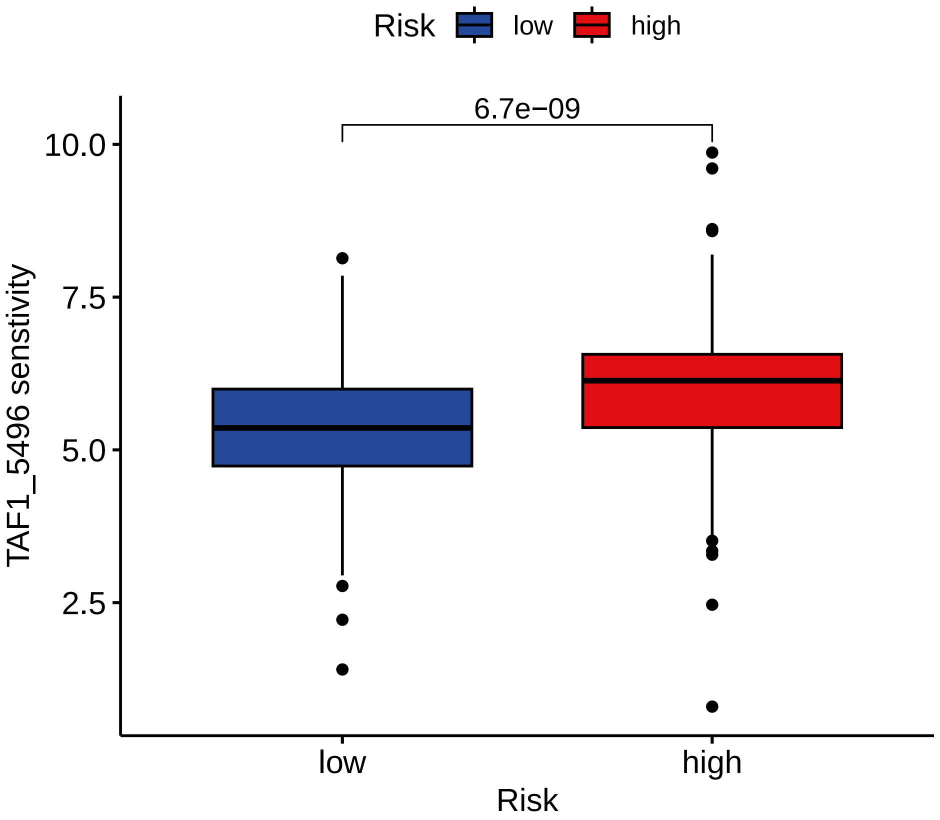
Risk
low
high
6.7e−09
10.0
7.5
5.0
2.5
TAF1_5496 senstivity
low
high
Risk

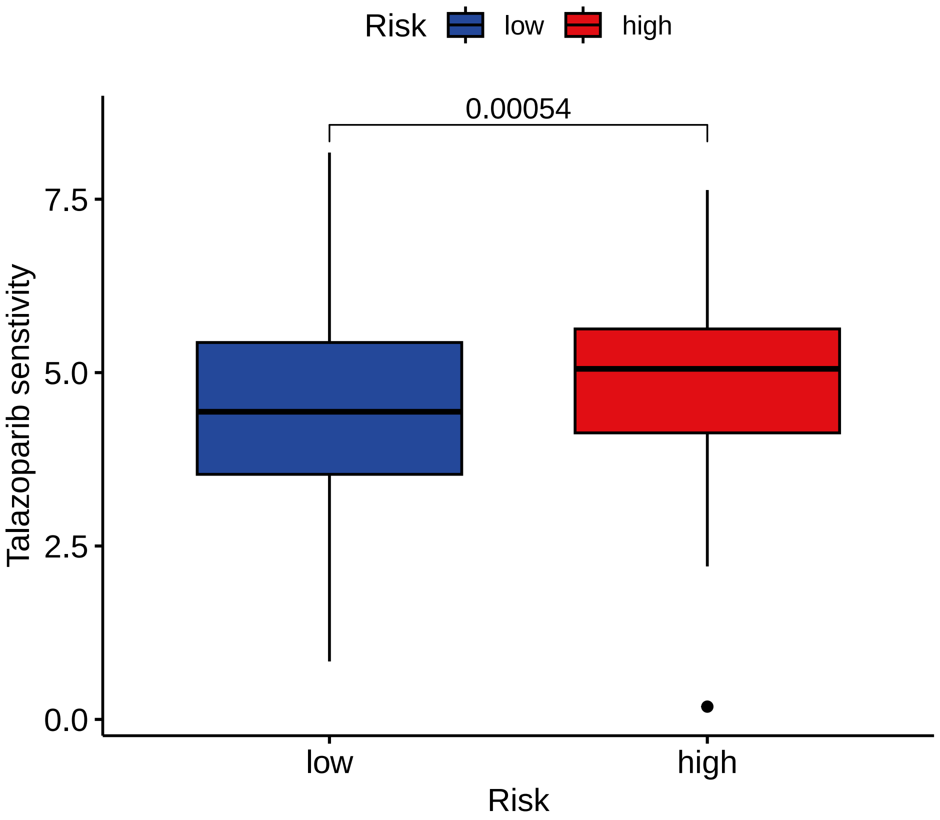
Risk
low
high
0.00054
7.5
5.0
2.5
0.0
Talazoparib senstivity
low
high
Risk

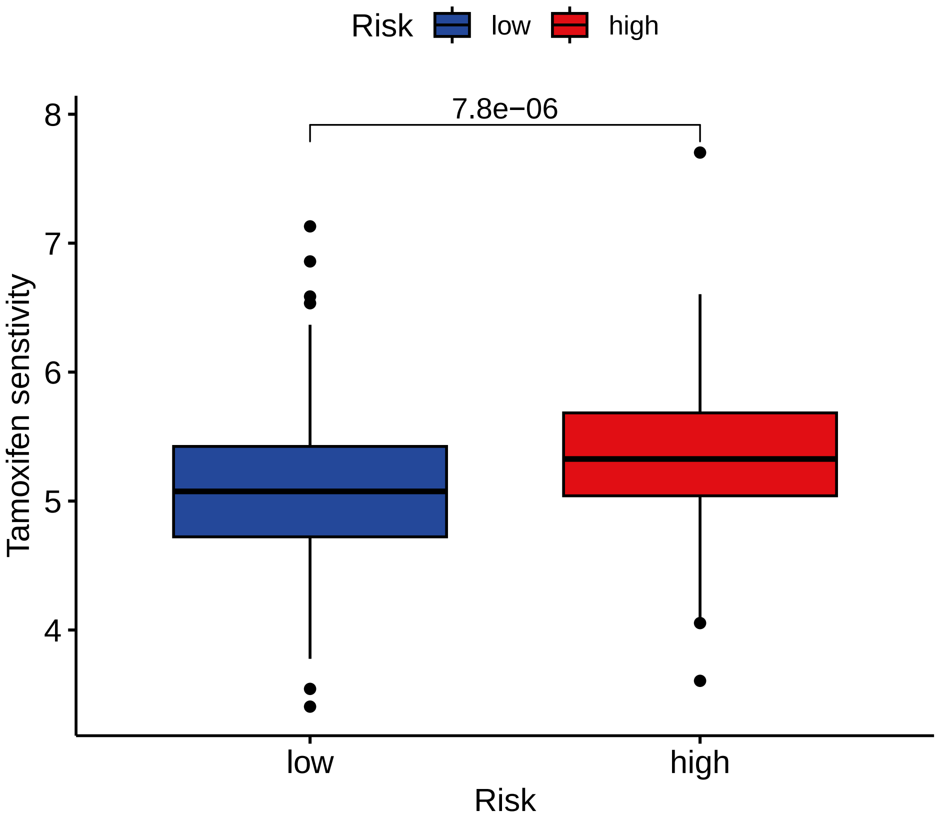

Risk
low
high
7.8e−06
8
7
6
5
4
Tamoxifen senstivity
low
high
Risk

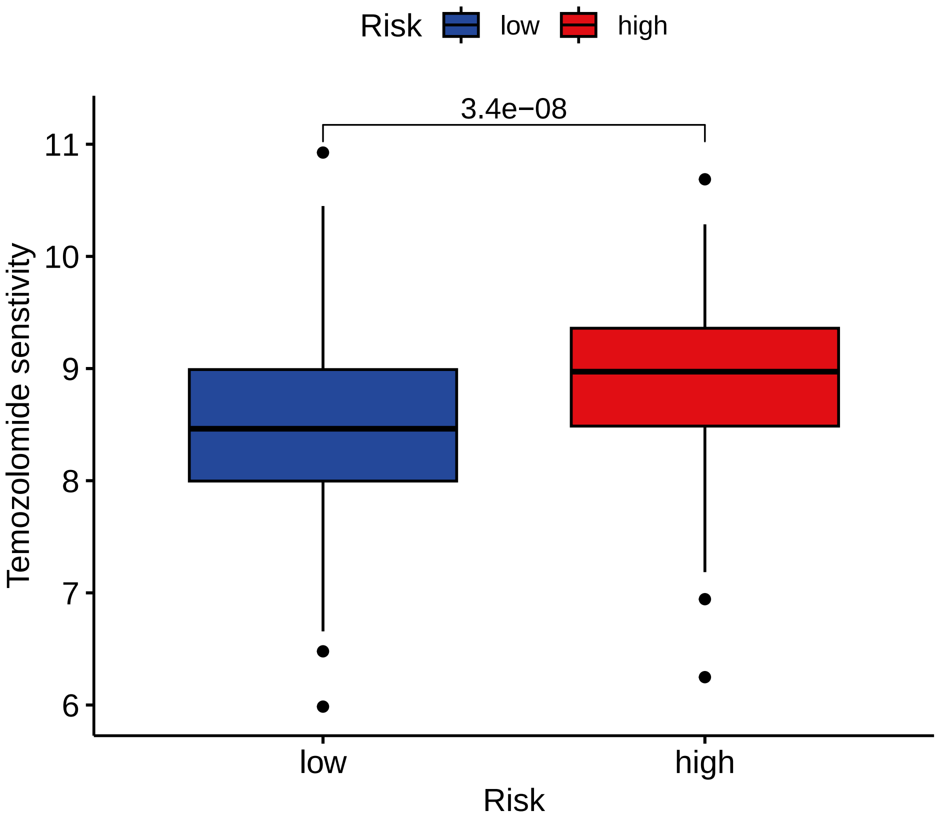

Risk
low
high
3.4e−08
Temozolomide senstivity
11
10
9
8
7
6
low
high
Risk

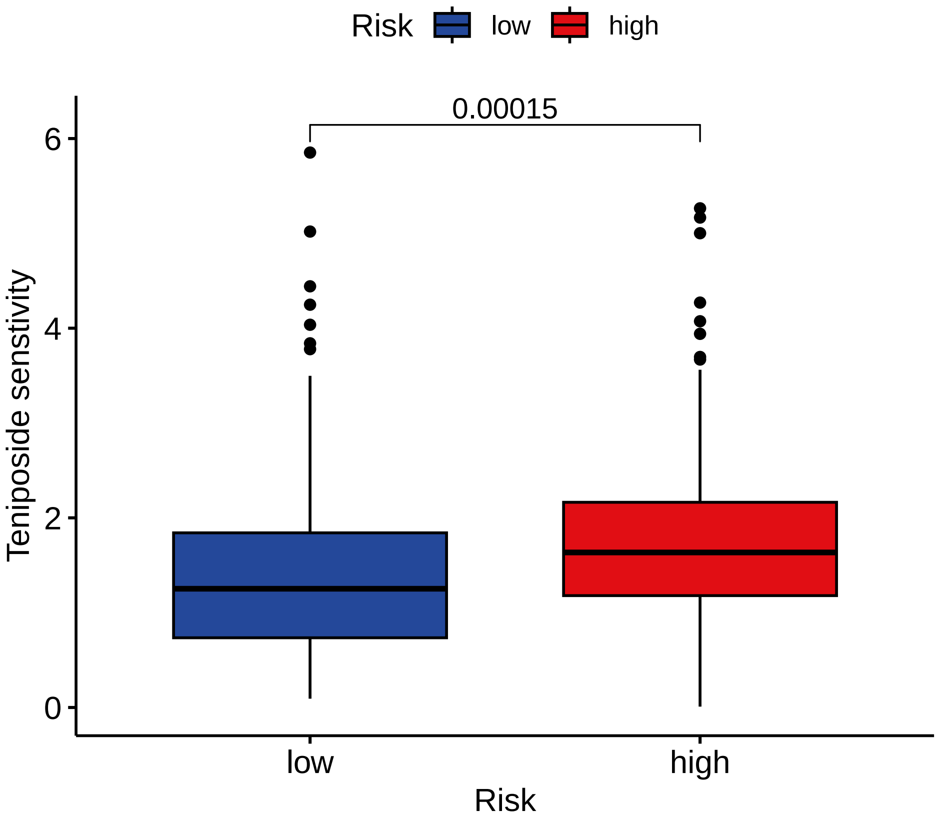

Risk
low
high
0.00015
Teniposide senstivity
6
4
2
0
low
high
Risk

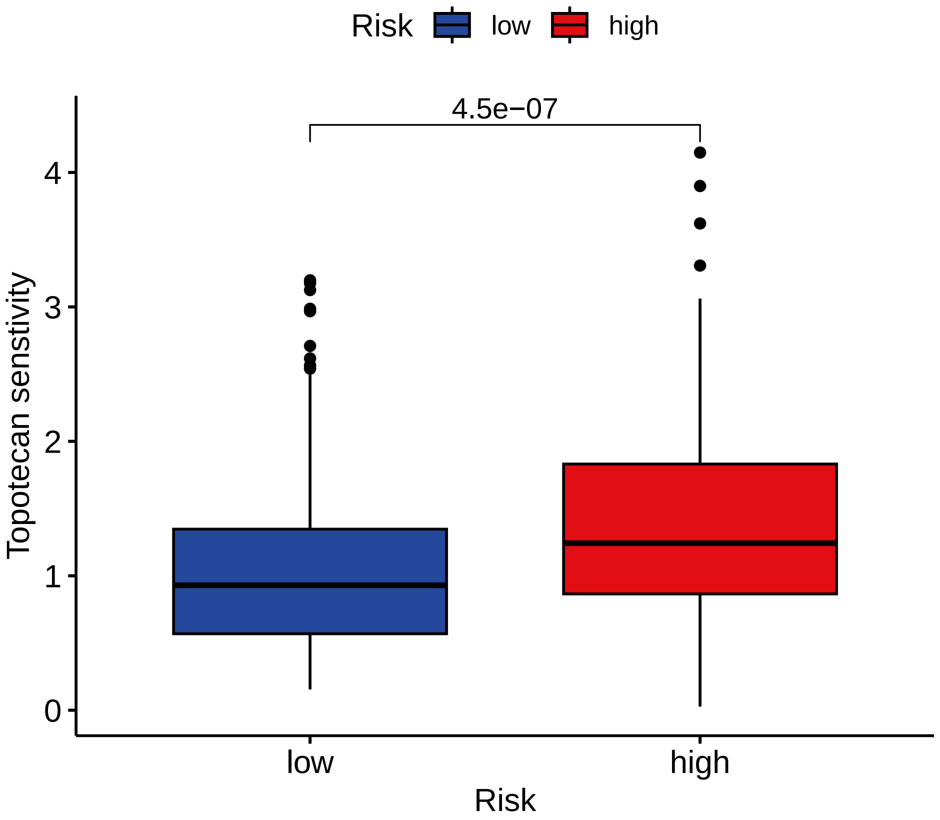
Risk
low
high
4.5e−07
Topotecan senstivity
0
1
2
3
4
low
high
Risk

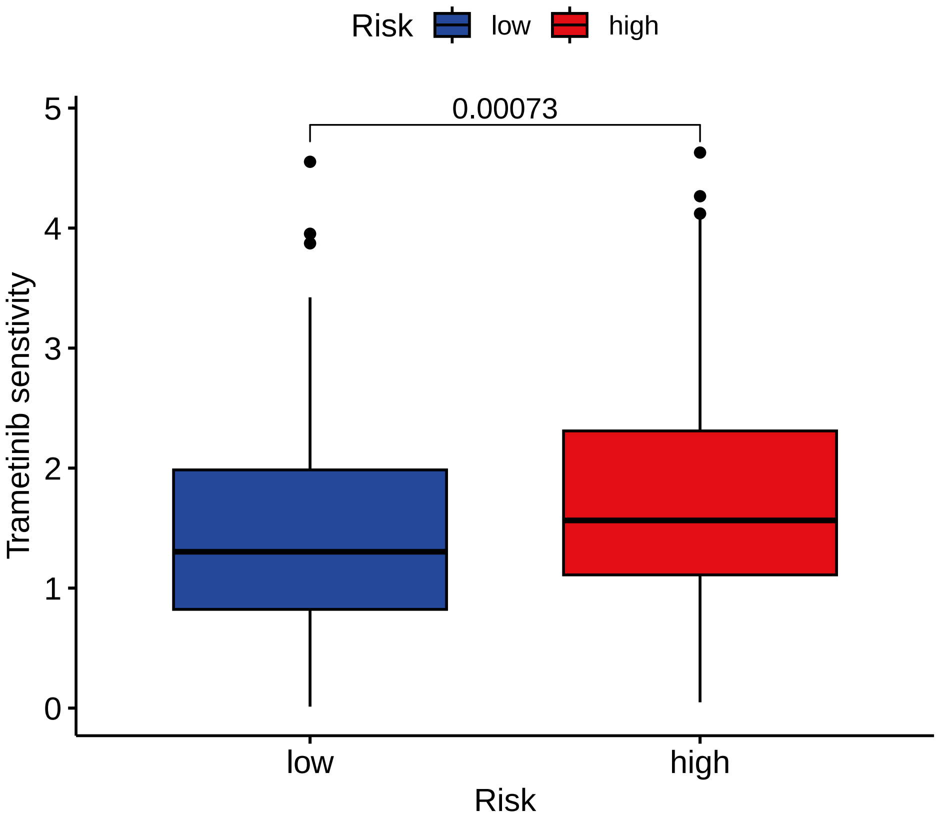

Risk
low
high
0.00073
Trametinib senstivity
5
4
3
2
1
0
low
high
Risk

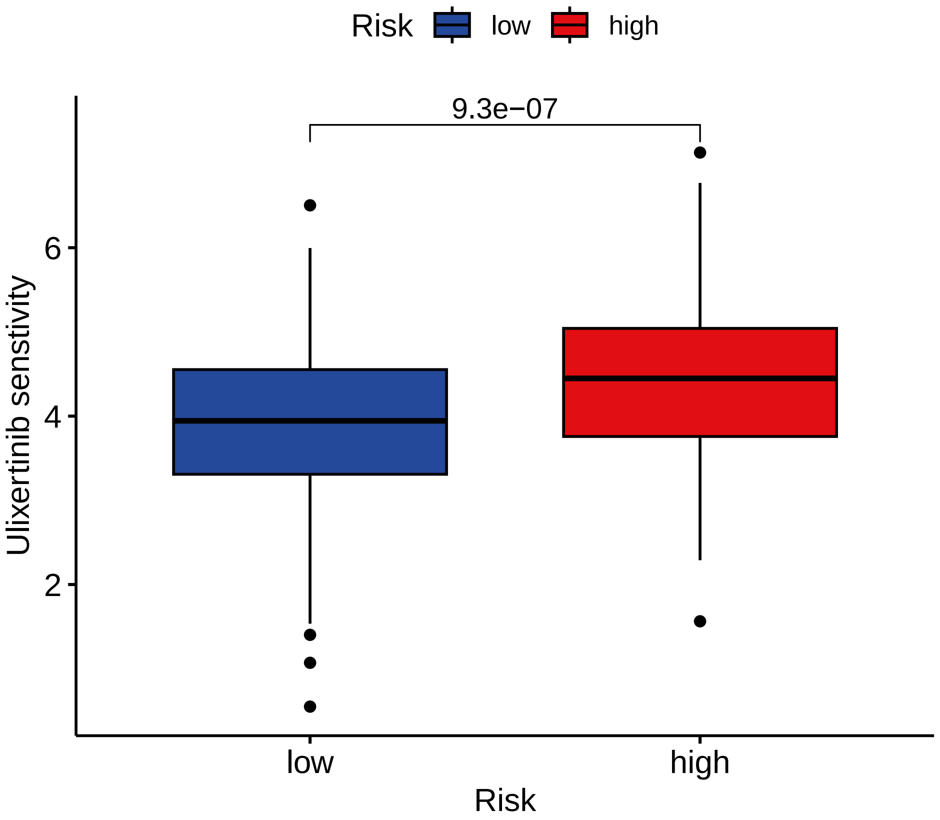

Risk
low
high
9.3e−07
Ulixertinib senstivity
6
4
2
low
high
Risk

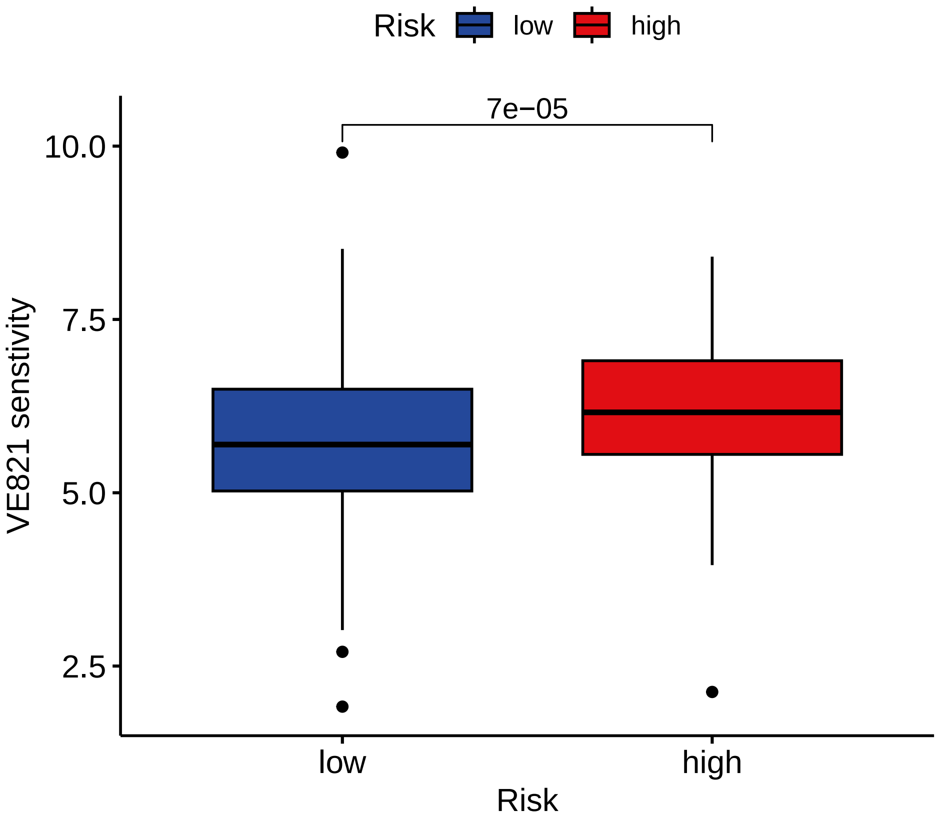

Risk
low
high
7e−05
10.0
7.5
5.0
2.5
VE821 senstivity
low
high
Risk

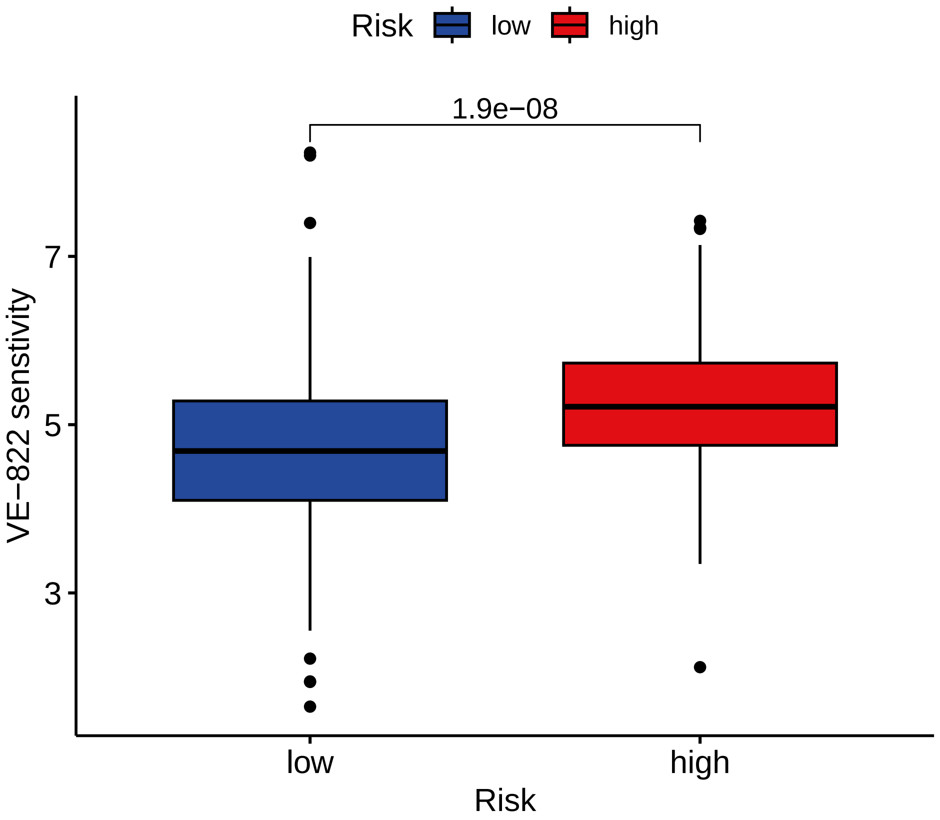

Risk
low
high
1.9e−08
VE−822 senstivity
7
5
3
low
high
Risk

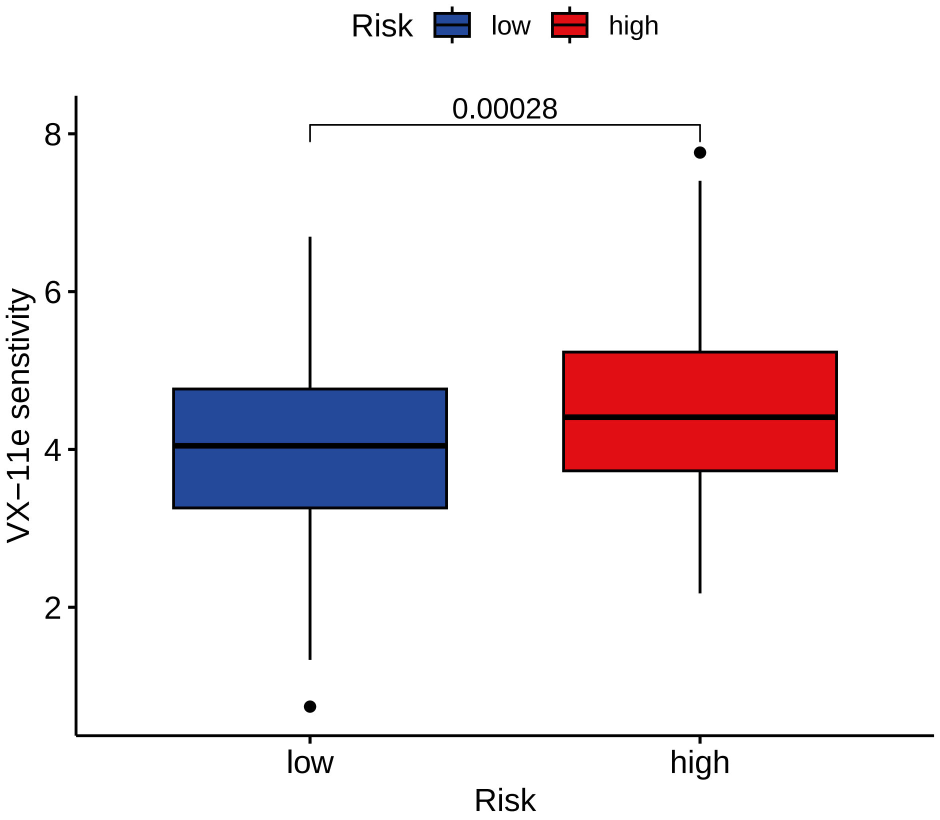
Risk
low
high
0.00028
8
6
4
2
VX−11e senstivity
low
high
Risk

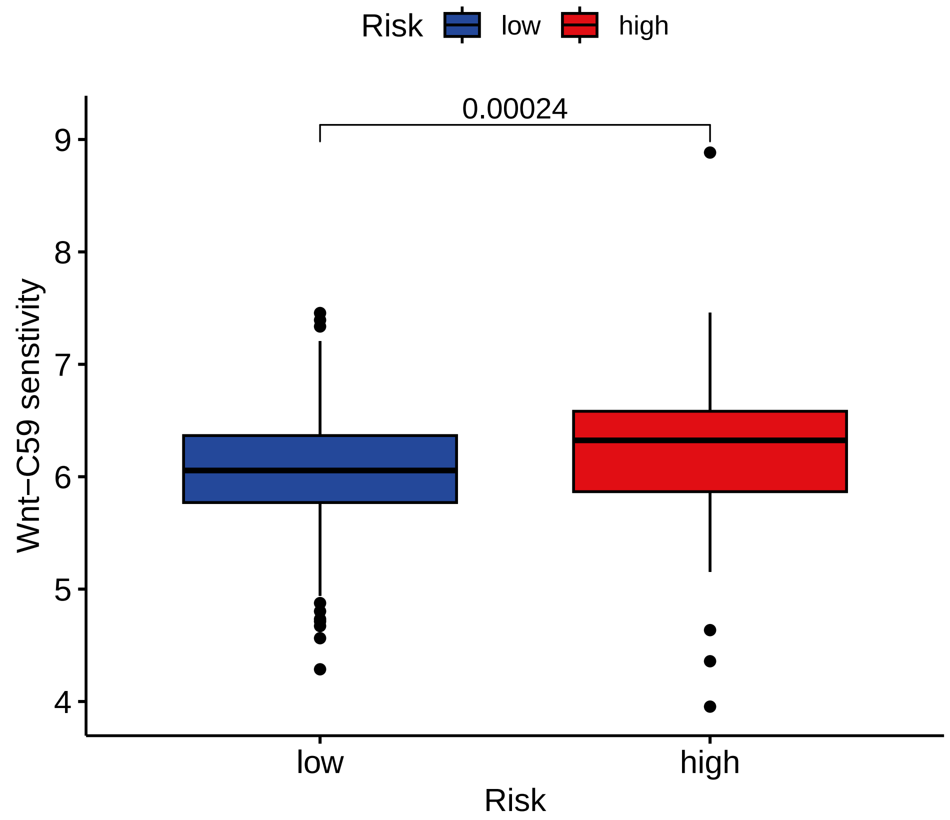
Risk
low
high
0.00024
Wnt−C59 senstivity
9
8
7
6
5
4
low
high
Risk
